# High-throughput comparison of gene fitness among related bacteria

**DOI:** 10.1186/1471-2164-13-212

**Published:** 2012-05-30

**Authors:** Rocio Canals, Xiao-Qin Xia, Catrina Fronick, Sandra W Clifton, Brian MM Ahmer, Helene L Andrews-Polymenis, Steffen Porwollik, Michael McClelland

**Affiliations:** 1 University of California, Irvine, CA, USA; 2 Institute of Hydrobiology, Chinese Academy of Sciences, Wuhan, China; 3 Washington University, Saint Louis, MO, USA; 4 Ohio State University, Columbus, OH, USA; 5 Texas A&M University System Health Science Center, College Station, TX, USA; 6 Vaccine Research Institute of San Diego, San Diego, CA, USA

## Abstract

**Background:**

The contribution of a gene to the fitness of a bacterium can be assayed by whether and to what degree the bacterium tolerates transposon insertions in that gene. We use this fact to compare the fitness of syntenic homologous genes among related *Salmonella* strains and thereby reveal differences not apparent at the gene sequence level.

**Results:**

A transposon Tn5 derivative was used to construct mutants in *Salmonella* Typhimurium ATCC14028 (STM1) and *Salmonella* Typhi Ty2 (STY1), which were then grown in rich media. The locations of 234,152 and 53,556 integration sites, respectively, were mapped by sequencing. These data were compared to similar data available for a different Ty2 isolate (STY2) and essential genes identified in *E. coli* K-12 (ECO). Of 277 genes considered essential in ECO, all had syntenic homologs in STM1, STY1, and STY2, and all but nine genes were either devoid of transposon insertions or had very few. For three of these nine genes, part of the annotated gene lacked transposon integrations (*yejM*, *ftsN* and *murB)*. At least one of the other six genes, *trpS*, had a potentially functionally redundant gene encoded elsewhere in *Salmonella* but not in ECO. An additional 165 genes were almost entirely devoid of transposon integrations in all three *Salmonella* strains examined, including many genes associated with protein and DNA synthesis. Four of these genes (*STM14_1498*, *STM14_2872*, *STM14_3360*, and *STM14_5442*) are not found in *E. coli*. Notable differences in the extent of gene selection were also observed among the three different *Salmonella* isolates. Mutations in *hns*, for example, were selected against in STM1 but not in the two STY strains, which have a defect in *rpoS* rendering *hns* nonessential.

**Conclusions:**

Comparisons among transposon integration profiles from different members of a species and among related species, all grown in similar conditions, identify differences in gene contributions to fitness among syntenic homologs. Further differences in fitness profiles among shared genes can be expected in other selective environments, with potential relevance for comparative systems biology.

## Background

When a library of transposon (Tn) integrations is created in a bacterial genome, some insertions are not recovered in the resulting pool of mutants, either because the insertion is in an essential gene or because the gene is required in the media used to grow the bacterium. This fact has been exploited extensively to identify genes under selection when growth conditions are changed [1-3].

Another potential utility of such data, that is explored here, is to compare different strains, serovars, and species to reveal apparent orthologs that have very different levels of fitness in different strains. We perform the first experiments to quantitate this phenomenon in *Salmonella*.

We used high-throughput sequencing to determine the location of tens of thousands of integration sites of a Tn5 derivative in the genome of *Salmonella enterica* serovar Typhimurium strain ATCC 14028 (STM1) and in *Salmonella enterica* serovar Typhi Ty2 (STY1) after growth in rich media (Luria Broth). Our datasets were compared to each other and to a series of other published data on the fitness of mutations in *Salmonella *[4-6] and *Escherichia coli* (ECO) [7-10], including a previously obtained transposon profile in a separate Ty2 isolate, STY2, which differs from STY1 by having mutations in *htrAaroC* and *aroD *[3].

Differences in the selective pressure on apparent orthologs in the related genomes (STM1, STY1, STY2 and ECO) are of interest because they likely reflect differences in the systems that interact with these otherwise functionally similar genes or their products.

## Results and discussion

### Profiling of a library of transposon insertions in *Salmonella* Typhimurium and Typhi

Five independent transposon libraries were constructed in *S*. Typhimurium ATCC 14028 (STM1) and two in *S.* Typhi Ty2 (STY1), using the EZ-Tn5 < KAN-2 > Promoter Insertion Kit (Epicentre Biotechnologies) (see methods), and grown in Luria broth (Additional file 1: Table S1). The genomic DNA directly adjacent to each transposon was obtained using a procedure similar to that described in Santiviago *et al. *[4] and sequenced as described in Additional file 2 and in Additional file 3: Figure S1.

We obtained a total of 16,642,379 first-strand Illumina sequencing reads of 100 bases in length. Sequences were subsequently filtered to reveal those reads that contained a complete primer including each unique barcode, followed by two bases of transposon (Tn) beyond the primer. The remainder of the sequence was mapped to the genome to determine the transposon integration site.

Each Tn integration site was generally represented by multiple reads, and these reads usually varied in the length of the *Salmonella* sequence due to the random DNA shearing used in the sequencing protocol. To reduce bias due to preferential PCR of some fragments, duplicate identical shear events were removed. The remaining reads for each transposon integration site were used to determine the number of different “shear events” for that transposon. This filtering resulted in the mapping of 234,152 and 53,556 Tn integration locations, with 2,827,876 and 313,585 unique shear events in STM1 and STY1, respectively. The average density of integrations into the genome was one every 20 bases with an average of about 12 shear events per site in STM1, and one every 90 bases with an average of about 6 shear events per site in STY1.

### A genome-wide survey of permitted transposon integrations

**Figure 1:**
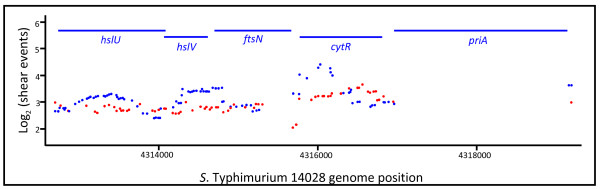
**Visualization of transposon integrations into an *S*. Typhimurium 14028 genome region.** The number of different sequencing reads originating from transposons (shear events) is plotted, averaged across 500 bases. Red, positive strand; blue, negative strand. The displayed region contains one gene necessary for growth in LB (*priA*) and one gene where only a segment shows selection (*ftsN*).

The transposon libraries used in these experiments were constructed and grown in rich media. Those regions of the genome with rare or absent transposon integrations include regions that are essential or under strong selection in rich media. A sizeable subset of these regions should also be essential under all growth conditions. An example of a profile of transposon integrations is shown in Figure 1, which displays a region in the STM1 genome that includes a known essential gene, *priA* (primosome assembly), and a gene with an essential region, *ftsN* (involved in cell division). Regions that were essential in Luria broth are identifiable in this plot as having no transposon integrations. Near-essential regions have a lower than average number of integration sites, usually accompanied by a lower number of shear events.

Our data also provide information on the orientation of each transposon, which can be informative. For example, in Figure 1, at the beginning of *cytR*, adjacent to *ftsN*, the negative strand contains far more transposons; this is the strand in which the heavily expressed antibiotic resistance marker of the transposon is oriented away from *ftsN*, likely making these integrations less disruptive. Many other examples of general selection and strand-specific selection are seen in Additional file 4: Figure S2, which shows a plot of the frequency of transposon insertions across the entire STM1 genome. A dramatic example of strand-specific selection is seen in the ribosomal RNA operons, for example at position 290,000 in the genome (Additional file 4: Figure S2). In this operon transposons are only permitted in the antisense strand, perhaps because truncated sense strand transcripts produced by the strong antibiotic resistance promoter in the transposon would disrupt ribosome assembly.

The transposon frequency analyses for all genes in STM1 (compared with known essentiality information for STY2 and ECO) and STY1 are presented in Additional file 5: Table S2 and Additional file 6: Table S3, respectively. Data for the *htrA*^*-*^*aroC*^-^ and *aroD*^*-*^ mutant STY2 were derived from two selections: a single passage on a solid medium (i) and six passages in Luria broth (ii).

### Essential genes in *E. coli*

Between *E. coli, S.* Typhimurium and *S*. Typhi, over 60% of protein coding genes are syntenic and have over 95% amino acid sequence identity [11]. There are 339 of the approximately 4,000 genes in ECO that have been reported to be essential in at least one of two comprehensive studies, the PEC (Profiling of E. coli Chromosome) database and the Keio collection [7-10] (sources are summarized in Table 1). Of these potentially essential genes, 277 genes are considered essential in both databases, and all 277 genes have syntenic homologs in STM1. These genes are listed in Additional file 5: Table S2.

**Table 1:** **Numbers of essential genes under laboratory conditions in relevant *E. coli*, *S. *Typhimurium and *S. *Typhi isolates**

**Species/serovar**	**Strain**	**Essential genes/non- essential**	**Method (type of mutagenesis, medium)**	**Reference**
*E. coli*	K-12 MG1655	302/4477	Published literature and MD (medium-scale) and LD (large-scale) deletion mutants (targeted mutagenesis, antibiotic medium 3)	Profiling of *E. coli* chromosome (PEC) database ( http://shigen.lab.nig.ac.jp/ECOli/pec/) [9,10]
*E. coli*	K-12 BW25113	303/3985	Single-gene deletion mutants (targeted mutagenesis, LB)	Keio collection [7]
*E. coli*	K-12 BW25113	299/3864	Single-gene deletion mutants (targeted mutagenesis, LB)	Update on the Keio collection [8]
*E. coli*	K-12 W3110	299/4109	Published literature	PEC database ( http://shigen.lab.nig.ac.jp/ECOli/pec/) [9,10]
*S.* Typhimurium	ATCC 14028	NA/1,023	Single-gene deletion mutants (targeted mutagenesis, LB)	[4]
*S.* Typhimurium	ATCC 14028	257/NA	Insertion-duplication mutagenesis (IDM) sequencing (random mutagenesis, LB)	[5]
*S.* Typhimurium	LT2	144 (LB and/or M9/glc)/NA	Metabolic reconstruction (*in silico* approach, M9/glc and LB)	[6]
*S.* Typhi	Ty2 (STY2)	356/4162	Random transposon mutagenesis and two types of growth^a^	[3]

^a^ Plating on an “aro mix” agar containing L-phe, L-trp, *p*-aminobenzoic acid and 2,3-dihydroxybenzoic acid (condition 1), six passages of growth in Luria broth (condition 2).

We ranked all *Salmonella* genes based on their density of transposons and the total number of shear events, and set a threshold of the 15^th^ percentile for “highly selected” genes. Exactly 549 genes in STM1, 582 genes in STY1, and 437 genes in STY2 met these criteria. Only six of the 277 genes essential in ECO were not among these highly selected genes in STM1 (Table 2): three narrowly missed the threshold (*folK, yejM* and *trpS*) and three had average amounts of transposon insertions (*murBftsN* and *degS*) indicating that mutants were not under selection in LB in this isolate. Two other genes did not meet the “selected” threshold in our STY1 assay (*yrfF, gpsA*) and one gene, *folA*, was not found to be selected in the published STY2 data [3].

**Table 2:** Essential genes in *E. coli *that are not as strongly selected in Typhimurium or Typhi*

***S. *Typhimurium 14028 gene symbol**	**Gene name**	**Gene description**	**Best hit in *S. *Typhimurium LT2**	**Best hit in *S. *Typhi Ty2**^**a**^	**Best hit in ECO**^**a**^	**STM1 transposons**	**STM1 reads**	**STY1 transposons**	**STY1 reads**	**STY2 transposons**^**b**^	**STY2 reads**^**b**^	**STY2 transposons**^**c**^	**STY2 reads**^**c**^
						**Percentile rank**							
*STM14_0106*	*folA*	Dihydrofolate reductase	*STM0087*	*t0090*	*b0048*	4	3	8	8	**19**	**19**	**46**	**60**
*STM14_0217*	*folK*	2-amino-4-hydroxy-6-hydroxymethyldihyropteridine pyrophosphokinase	*STM0183*	*t0191*	*b0142*	12	**22**	**18**	**16**	**25**	**56**	**16**	**21**
*STM14_2754*	*yejM*	Putative hydrolase	*STM2228*	*t0626*	*b2188*	14	**16**	**30**	**25**	13	**19**	**17**	**20**
*STM14_4041*^*d*^	*degS*	Serine endoprotease	*STM3349*	*t3265*	*b3235*	**50**	**34**	14	14	6	7	4	4
*STM14_4193*	*trpS*	Tryptophanyl-tRNA synthetase	*STM3481*	*t4024*	*b3384*	**18**	**16**	14	14	**78**	**64**	12	12
*STM14_4208*	*yrfF*	Intracellular growth attenuator protein	*STM3495*	*t4011*	*b3398*	1	4	**19**	**19**	**70**	**42**	12	**27**
*STM14_4460*	*gpsA*	NAD(P)H-dependent glycerol-3-phosphate dehydrogenase	*STM3700*	*t3819*	*b3608*	10	10	14	**17**	**45**	**46**	8	11
*STM14_4921*	*ftsN*	E cell division protein	*STM4093*	*t3525*	*b3933*	**42**	**38**	14	15	**20**	**27**	15	15
*STM14_4971.J*	*murB*	UDP-*N*- acetylenolpyruvoylglucosamine reductase	*STM4136*	*t3489*	*b3972*	**42**	**48**	**26**	**22**	8	**24**	13	**21**

* Details for all genes can be found in Additional file 5: Table S2. Genes were ranked by the number of transposon insertions and number of independent reads per gene. Ranks above the 15^th^ percentile are shown in bold.

^a^ Best hits are listed only if gene is syntenic with *S*. Typhimurium strain 14028 and has at least 95% sequence identity.

^b^ Growth on L-agar with “aro mix” (L-phe, L-trp, *p*-aminobenzoic acid and 2,3-dihydroxybenzoic acid) [3].

^c^ Growth after six passages in LB broth [3].

^d^ Considered essential in STY2 [3].

Visual inspection of Additional file 4: Figure S2 revealed that in three of the genes that are essential in ECO but seemingly not essential in STM1, part of the respective gene was, in fact, devoid of any transposon insertions: *yejM*, a putative hydrolase; *ftsN*, which encodes a cell division protein; and *murB*, a UDP-*N*-acetylenolpyruvoylglucosamine reductase. Figure 1 shows the *ftsN* gene as an example. These genes can apparently be disrupted in certain locations without losing their essential function. Thus, because of the high density of transposon integration data, we were able to reveal those cases where only part of the gene is essential.

The fourth of the six cases, *trpS,* encodes a tryptophanyl-tRNA synthetase. It may tolerate transposon insertions in STM1 and STY because of the presence of a distant paralog (*trpS2*), which does not exist in ECO. TrpS2 may substitute the TrpS function, although it is only 28% identical [12].

The fifth gene essential in ECO and not under strong selection in STM1 is *degS*, a serine endoprotease. This gene was under strong selection in STY2 [3]. That strain (but not our STY1) is an *htrA* mutant, a paralog of *degS *[13]. In our STY1 data, transposon insertion into *degS* was somewhat diminished, but not enough to qualify the gene for the “selected” category, suggesting at least some effect of the lack of a functional HtrA in STY2. However, in STM1 there is no evidence for any degree of transposon underrepresentation in *degS*, and it seems likely that another paralog, as yet undefined, can perform the proteolytic activity of DegS in this strain, if needed.

Finally, *folK* was somewhat underrepresented in transposon insertion frequency in STM1, but not to a degree that warranted inclusion in the “selected” category.

There are three other genes that are essential in ECO and STM1, but seemed not strongly selected in our STY1 survey and/or STY2. The *folA* gene, involved in folate biosynthesis, was strongly selected in STM1 and STY1 but did not show as much selection in STY2, especially after six passages in LB. Lastly, *yrfF* and *gpsA* are two genes essential in ECO that did not meet the “selected” threshold in our STY1 assay. Both these genes were under strong selection in STM1 and in STY2 after six passages in LB, indicating that mutations cannot be maintained for many passages in this medium. The *yrfF* (*igaA*) gene encodes an intracellular growth attenuator protein; and *gpsA* encodes a NAD(P)H-dependent glycerol-3-phosphate dehydrogenase. IgaA has been described as essential in *S*. Typhimurium unless there are additional mutations in the RcsCDB system, because it acts as a repressor of this system [14,15]. Expression of *igaA* is positively regulated by Lon and negatively modulated by Hnr (MviA) through the transcriptional regulator RpoS [16]. *S*. Typhi Ty2 carries a defect in the *rpoS* gene [17,18], which may explain the lesser degree of selection of *igaA* in STY.

### Genes that are not essential in *E. coli* but are under strong selection in Typhimurium and Typhi

The list of essential genes in ECO is stringent, generally including the inability to obtain a viable deletion mutant in rich medium. In contrast, the measure available from transposon integrations in STM1 and STY reveals genes that were under strong selection, but not necessarily essential. A group of 159 such genes that are under selection in all *Salmonella* (i.e. STM1, STY1 and STY2) but not essential in ECO were identified (Additional file 5: Table S2). This list included many of the genes that might be expected to be under selection, such as genes encoding parts of the ribosome and its accessory proteins, as well as some genes encoding replication components. However, there were at least 14 genes that still have an unknown or a poorly understood function (*ybaB*, *ybeD*, *ybeY*, *phoL*, *ycaR*, *ycdC*, *yciM*, *yciS*, *ygfZ*, *yhaL*, *yheM, yheN*, *wecF*, and *yigP)*. Given the conservation of these genes between *Salmonella* and *E. coli*, these are particularly interesting targets for future studies to determine their exact function.

The approximately 900 genes shared by STM1 and STY that have no synteny in ECO (*Salmonella*-specific genes) yielded only two strongly and consistently selected genes: *STM14_5442* and *STM14_2872,* which both encode putative cytoplasmic proteins. However, Santiviago *et al.* reported successful knockout mutations in both of these genes in this same strain, so they are likely not essential [4]. However, the genes may have an effect on growth, because mutants in these genes grow poorly in competitive assays (unpublished data).

### Genes under greater selection in Typhimurium than in Typhi

Genes that were under stronger selection in STM1 than in STY when the transposon libraries were grown in LB are depicted in Table 3A. The most dramatic difference was in *hns*, with an almost equal effect on the near adjacent gene *hnr*. Whereas *hns* is essential in *Salmonella* unless certain second site mutations are also present [19], no selection against insertion in this gene was found in STY in this study. The most likely explanation for this phenomenon is that *S*. Typhi Ty2 is known to contain a mutation in the *rpoS*gene [17,18]. Mutations in this gene permit second site mutations in *hns* to be viable [19], and *hnr* (*mviA*) is a response regulator which post-transcriptionally modulates RpoS levels [20]. Interestingly, *stpA*, which encodes a 53% identical paralog of *hns*, also showed a greater tolerance for transposons in STY than in STM1. Unlike *hns*, viable *hnr* mutants can be obtained in *S*. Typhimurium [21], even though this gene appears to be strongly selected in STM1. Hnr participates in RpoS stability by acting as an adaptor for degradation by the ClpXP protease [22]. Mutants in *hnr* show reduced growth rate because of an increased RpoS stability, which increases transcription of genes involved in growth arrest and resistance to a variety of stresses [20]. The selection against *hnr* mutations seen in STM1 might be due to the non-advantageous phenotype of slow cell division in these mutants when they are in competitive growth.

**Table 3:** Genes displaying prominent differences in selection between Typhimurium and Typhi*

***S. *Typhimurium 14028 gene symbol**	**Gene name**	**Gene description**	**Best hit in *S. *Typhimurium strain LT2**	**Best hit in *S. *Typhi Ty2**^**a**^	**Best hit in ECO**^**a**^	**STM1 transposons**	**STM1 reads**	**STY1 transposons**	**STY1 reads**	**STY2 transposons**^**b**^	**STY2 reads**^**b**^	**STY2 transposons**^**c**^	**STY2 reads**^**c**^
						**Percentile rank**							
**A. Examples of genes with stronger selection in STM1 than in STY1 and STY2**													
*STM14_0199.RJ*	*yacC*		*STM0167*	*t0172*	*b0122*	12	13	**57**	**70**	**53**	**51**	**59**	**51**
*STM14_1028.R*	*potF*	Putrescine ABC transporter putrescine-binding protein	*STM0877*	*t2019*	*b0854*	14	15	**47**	**63**	**35**	**24**	**38**	**32**
*STM14_1092*		Hypothetical protein		*t1967*		13	10	**61**	**59**	**35**	**42**	**75**	**66**
*STM14_1127*	*ycbL*	Putative metallo-beta- lactamase	*STM0997*	*t1937*	*b0927*	13	13	**30**	**40**	**27**	**34**	**23**	**36**
*STM14_1141*	*xisW*	Excisionase	*STM1006*	*t1928*		11	9	**72**	**62**	**53**	**30**	**43**	**24**
*STM14_1152*	*dnaC*	Putative replication protein	*STM1015*	*t1917*		16	16	**27**	**36**	**34**	**31**	**41**	**32**
*STM14_1548*	*yeaM*	Putative regulatory protein	*STM1279*	*t1159*	*b1790*	14	17	**50**	**59**	**36**	**25**	**44**	**38**
*STM14_1599*	*celG*	Hypothetical protein	*STM1317*	*t1196*	*b1733*	15	17	**67**	**75**	**54**	**46**	**50**	**48**
*STM14_1682*	*orf70*	Hypothetical protein	*STM1388*	*t1256*	*b1675*	12	11	**32**	**71**	**34**	**27**	**37**	**29**
*STM14_1877*^*d*^		Putative coiled-coil protein	*STM1554*	*t1468*		15	16	**39**	**37**	**79**	**81**	**78**	**81**
*STM14_1977*^*d*^		Putative periplasmic binding protein	*STM1633*	*t1536*		13	16	**34**	**27**	**86**	**85**	**86**	**83**
*STM14_1978*^*d*^		Putative ABC transporter permease component	*STM1634*	*t1537*		6	7	17	**44**	**87**	**91**	**85**	**90**
*STM14_2116*	*hns*	Global DNA-binding transcriptional dual regulator H-NS	*STM1751*	*t1662*	*b1237*	5	3	**32**	**29**	**91**	**89**	**35**	**91**
*STM14_2119*	*hnr*	Response regulator of RpoS	*STM1753*	*t1664*	*b1235*	1	1	**63**	**58**	**69**	**59**	**48**	**31**
*STM14_2120*	*ychK*	Hypothetical protein	*STM1754*	*t1665*	*b1234*	11	11	**64**	**81**	**64**	**61**	**62**	**46**
*STM14_2140*	*chaB*	Cation transport regulator	*STM1770*	*t1679*	*b1217*	12	11	**30**	**29**	**63**	**60**	**73**	**49**
*STM14_2176*	*ldcA*	L,D-carboxypeptidase A	*STM1800*	*t1077*	*b1192*	14	15	**43**	**37**	**28**	**28**	**39**	**39**
*STM14_2186*^*d*^	*gsnB*	Putative cytoplasmic protein	*STM1809*	*t1068*		18	14	**39**	**30**	**43**	**58**	**59**	**70**
*STM14_2278*		Hypothetical protein	*STM1873*	*t1004*	*b1839*	14	13	**37**	**57**	**48**	**28**	**54**	**38**
*STM14_2685*		Putative 1,2-dioxygenase	*STM2178*	*t0677*		15	18	**32**	**33**	**31**	**29**	**24**	**32**
*STM14_2708.RJ*		Putative DNA-binding protein	*STM2195*	*t0660*		17	15	**51**	**44**	**65**	**36**	**62**	**49**
*STM14_2745*		Bicyclomycin/multidrug efflux system		*t0634*		16	14	**65**	**47**	**66**	**43**	**73**	**68**
*STM14_3050*	*purC*	Phosphoribosylaminoimidazole- succinocarboxamide synthase	*STM2487*	*t0372*	*b2476*	11	11	**28**	**32**	**47**	**34**	**34**	**30**
*STM14_4596*^*d*^		Pseudogene	*STM3806*	*t0121*		14	12	**78**	**49**	**53**	**55**	**36**	**51**
*STM14_4883*	*cpxP*	Periplasmic repressor	*STM4060*	*t3559*	*b3913*	16	16	**27**	**46**	**38**	**41**	**43**	**34**
*STM14_5142*^*d*^		Putative cytoplasmic protein	*STM4276*	*t4182*		15	13	**46**	**28**	16	**49**	19	**47**
*STM14_0109*	*ksgA*	Dimethyladenosine transferase	*STM0090*	*t0093*	*b0051*	**37**	**28**	5	5	19	13	5	5
*STM14_2281*	*holE*	DNA polymerase III subunit theta	*STM1876*	*t1001*	*b1842*	**63**	**70**	11	11	9	8	25	15
*STM14_2498*		Hypothetical protein	*STM2011.1n*	*t0860*		**51**	**43**	13	12	8	10	14	17
*STM14_2665*^*e*^		Hypothetical protein	*STM2161*	*t0694*	*b2128*	**37**	**53**	14	13	8	7	14	11
*STM14_3017*	*eutA*	Reactivating factor for ethanolamine ammonia lyase	*STM2459*	*t0399*	*b2451*	**26**	**29**	16	16	10	11	16	16
*STM14_3267*^*e*^		Hypothetical protein	*STM2666*	*t2621*	*b2598*	**81**	**84**	17	14	9	7	19	13
*STM14_4041*^*e*^	*degS*	Serine endoprotease	*STM3349*	*t3265*	*b3235*	**50**	**34**	14	14	6	7	4	4
*STM14_4465*	*yibP*	Hypothetical protein	*STM3705*	*t3814*	*b3613*	**63**	**65**	19	18	11	11	10	11
*STM14_4747*	*yifL*	Putative outer membrane lipoprotein	*STM3946*	*t3351*	*b4558*	**77**	**65**	12	12	**22**	17	18	16
*STM14_5345*	*treC*	Trehalose-6-phosphate hydrolase	*STM4453*	*t4488*	*b4239*	**50**	**49**	13	13	10	8	5	9

* Details for all genes can be found in Additional file 5: Table S2. See Table 2 for notes a, b, and c. Ranks above the 20^th^ percentile are shown in bold.

^d^ Knockout mutant has been created in STM [4].

^e^ Considered essential in STY2 [3].

At least 26 additional genes appeared to be under strong selection in STM1 but not in either STY. This class includes, among others, the putrescine ABC transporter *potF*; an excisionase; a cation transport regulator *chaB*; the L,D-carboxypeptidase A *ldcA*; the phosphoribosylaminoimidazole-succinocarboxamide synthase *purC*; and *cpxP*, a periplasmic repressor of the envelope stress response pathway. Viable mutants were obtained in this same Typhimurium strain for six of these genes [4] (listed in Table 3). It is not yet known if the remaining 20 genes can be deleted but it is likely that most, if not all, are not essential.

### Genes under greater selection in Typhi than in Typhimurium

Genes that were under greater selection in STY1 and STY2 compared to STM1 are listed in Table 3B. Among the 10 genes under consistent selection in STY2 (both after passage in LB and after growth on aro-mix agar [3]) and our own STY1 data, but not in STM1, were four genes that encode hypothetical proteins and the previously mentioned *degS*. Other genes in this class include *eutA*, involved in the ethanolamine utilization pathway, the dimethyladenosine transferase *ksgA* and *treC,* a trehalose-6-phosphate hydrolase. The gene *holE* is an interesting example that is more strongly selected in STY than in STM1. This gene encodes the theta subunit of DNA polymerase III. The STM14_5586 protein encoded on the virulence plasmid in STM1, which is not present in STY, is a paralog that may partially substitute for *holE *[23].

### Pseudogenes

Integrations in genes that are thought to be pseudogenes in STY and intact in STM1, or vice versa, were inspected. Of approximately 60 putative pseudogenes annotated in the *S*. Typhimurium 14028 genome, four showed strong selection in STM1 and are annotated as intact in *S*. Typhi Ty2: *STM14_1358*, *STM14_1498.L*, *STM14_1778*, and *STM14_4596*. Only one of them, *STM14_1358*, has an ortholog in ECO (*yceQ*) and, interestingly, has been reported as essential in this species. In *STM14_1358* and *STM14_1498.L*, levels of selection in STM1 were similar to the levels in both STY. In the other two cases, there was no selection in at least one of the two STY isolates.

Of approximately 200 pseudogenes in *S*. Typhi Ty2, four were strongly selected in at least one of the STY: *eda*, a keto-hydroxyglutarate-aldolase/keto-deoxy-phosphogluconate aldolase; *astA*, an arginine succinyltransferase; *t2152* (*STM14_0843*), a putative glycosyltransferase involved in cell wall biogenesis; and *t3548* (*STM14_4894*), a putative cytoplasmic protein. The *eda* gene is the only case showing a strong selection in both STY1 and STY2.

In *S*. Typhi Ty2, an RNA-seq analysis of the transcriptome was recently published [24] and concluded that the vast majority of pseudogenes had low or undetected transcription. Only nine pseudogenes showed high levels of transcription, none of which correspond to our four strongly selected pseudogenes. A region annotated as a pseudogene and showing strong selection in any environmental condition suggests that a function is encoded in this region, whether it is a partial protein or a regulatory region.

### Differences between transposon mutant libraries of two strains of Typhi

In our study, 53,556 transposon integration sites for STY1 were determined. In a previous work, 370,000 insertion sites were identified in STY2. Over 100 genes showed a difference in fitness between these two strains. Some of these differences may be attributable to the growth conditions used in the two studies. Our STY1 data are from a single LB growth passage whereas the STY2 data were from L-agar supplemented with aromatic compounds as well as from six passages in LB. Furthermore, STY2 is an attenuated strain, CVD908-*htrA *[3], which differs from STY1 in that it carries additional mutations in *aroCaroD*, and *htrA *[25]. Deletions in the *aro* genes, which encode enzymes involved in the shikimate biosynthesis pathway, render bacteria auxotrophic for the aromatic amino acids *p*-aminobenzoate (pABA) and 2,3-dihydroxybenzoate [26]. These *aro* mutations also result in the inability to produce ubiquinone and menaquinone, leading to respiration defects [27], and in defects in some components of the cell envelope [28], unless aromatic precursors are added to the medium. HtrA is a serine protease involved in the degradation of aberrant periplasmic proteins. An *htrA* mutant presents more susceptibility to oxidative stress than the wild type [29].

We found 17 transposons in *htrA* and one each in *aroC* and *aroD* in STY1. The number of transposons in *htrA* was close to the average random transposon insertion frequency (15.9) whereas *aroC* and *aroD* showed selection in both STY1 and STY2. The apparent selection of *aroC* and *aroD* in STY2 is explained by the fact that these genes were knocked out in this strain, resulting in a much smaller gene remnant as transposon target area. The *htrA* mutation may explain at least some differences between the two strains of STY, such as the selection of the periplasmic protein HlpA and the strong selection of DsbA after six passages in LB in STY2, but not in our STY1 study. In *E. coli*, the *skp* (*hlpA* in *Salmonella*) *degP* (*htrA* in STM) double mutant is lethal [30] and the *dsbA degP* double mutant shows reduced growth [31].

STY1 showed selection in some *Salmonella* Pathogenicity Island 2 (SPI-2) genes [32,33]. Some of these were also selected in STM1 (*ssaIssaHssaRssaTsifB*) but not in STY2. Some other SPI genes were interesting from a regulatory point of view. The *hilC* and *rtsA* SPI-1 regulators were selected in STY1, but not in STY2 [34]. RtsA is encoded in an operon that also includes the similarly selected *rtsB*, whose product represses the master regulator of the flagellar regulon, *flhDC *[35].

### Flagellar genes

Flagellar genes show different patterns of selection comparing growth in LB broth under aeration (STM1, STY1 and STY2) versus on LB agar (STY2). Flagella and motility are highly regulated in *S.* Typhimurium and involve over 60 genes integrated in a hierarchy of controlled transcription [36]. The flagellar structure consists of three components: the basal body, the hook and the filament. The basal body consists of three rings and a rod which transverses the periplasmic space. These three components are expressed coordinately, first the hook-basal body (HBB) and later the filament, and assembled via a flagellar type III secretion apparatus [37]. The *flgM* gene encoding the anti-sigma 28 negative regulator of the synthesis of the flagellar filament, was selected in broth media, but not on LB agar. FlgM binds to the sigma 28 factor to prevent transcription of late flagellar genes before the completion of HBB structure [38]. In contrast, *flgCflgJfliIfliK*, and *fliO* were selected only when bacteria were grown on an agar surface. FlgC is one of the structural rod components and FlgJ is the capping protein of the rod which also possesses muramidase activity [39]. FliI is an ATPase that forms part of the flagellar type III export apparatus, although it is not essential [40]. FliK regulates the length of the hook by switching the secretion specificity from rod-hook type substrates to filament-type substrates [41]. FliO is one of the integral membrane proteins of the flagellar secretion system which seems to have a role in stabilizing another protein of this system, FliP [42]. These five proteins are components of the basal body or the flagellar export apparatus, or interact with these structures. Furthermore, *fliT*, which encodes a protein that acts as a repressor of flagella biosynthesis [43], was selected after six passages in LB in STY2 [3]. The flagellar gene *flhE* showed selection on agar growth and after six passages in LB in STY2 [3]. Although it is known that the lack of FlhE does not affect flagella biogenesis or swimming motility, these mutants are defective in swarming motility [44]. In agreement with these results, Wang *et al.* reported that flagellar genes were regulated in a surface-specific manner [45]. Overall, our data indicates selection for genes encoding inhibitors of flagellar biogenesis when bacteria are grown in LB broth, such as *flgM* and *fliT*; and selection for genes necessary for flagellar biosynthesis when bacteria are grown on agar.

### Comparison of fitness data with previous efforts to identify essential genes in *Salmonella*

We compared our data (Additional file 5: Table S2) to previous experiments that had sought to identify essential and non-essential genes in Typhimurium (Table 1). We previously reported 1,023 genes that give viable mutants in rich media in the same strain of Typhimurium as used here [4]. At least 38 of these genes are “selected” in our study (among the 15% with the lowest density of transposon integrations and shear events). The differences may be attributable to measuring essentiality, which is absolute, versus fitness selection, which is relative. In another study, 257 genes were identified as potentially essential genes for *in vitro* growth in LB using an insertion-duplication mutagenesis (IDM) strategy based on a temperature-sensitive integration plasmid [5]. Of these 257 genes, only 104 were under strong selection in STM1 in our data. It was previously suggested that some of these putative essential genes might be non-essential [46]. It appears that IDM may give a high rate of false positives for essential genes. In another study, a prediction using metabolic reconstruction identified 144 genes that could be essential for growth of *S*. Typhimurium LT2 in LB, of which 71 were already known to be essential in *E. coli *[6]. Of the remaining 74 potentially novel essential phenotypes, 19 were under strong selection in STM1. Additionally, 57 genes were predicted to be essential only in minimal media. Six of these genes were under strong selection in rich media in STM1. Given the considerable discrepancy in the observed and predicted genes under selection, fitness data have the great potential to improve systems biology models in the future.

### An extended motif for transposon integration

**Figure 2:**
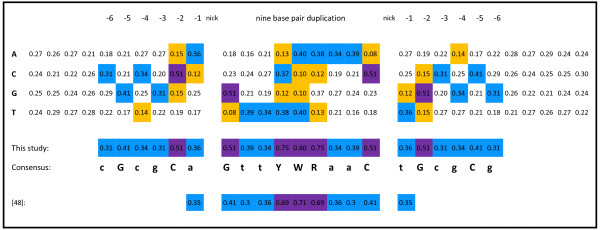
**An extended motif for transposon integration hot spots.** Integration sites that occurred in the same location and were overrepresented in more than one transposon library were aligned and a consensus was obtained. Base frequencies over 50% are purple, between 30 and 50% light blue, and below 16%, orange.

Tn5 transposition can occur into almost any sequence. Indeed, we were unable to find any bias in integration targeting when we used all the transposon integration data we had available and a variety of motif-finding programs (data not shown), indicating that the vast bulk of integration sites were largely randomly distributed. However, hotspots, where Tn5 integration is preferred, have been reported [47]. Goryshin *et al*. sequenced 198 integration sites in a plasmid and identified a short degenerate consensus palindromic motif where integrations were more frequent: a-GntYWRanC-t [48]. The dash indicates the site of cleavage of the target that is then duplicated on either side of the transposon integration. To examine whether hotspots for integration in our data might further refine this motif we filtered over 300,000 integration sites for those sites that occurred in more than one of our independent transposon libraries. Then we filtered for integration sites represented by more than the average number of total shear events. This strategy yielded 654 sites that were among the most highly preferred targets for integration. By aligning the sequences surrounding these sites, we were able to refine the originally reported motif and further extend the motif by five bases on either side, including a highly conserved C and G located two and five bases upstream of the nick in the target DNA: cGcgCa-GttYWRaaC-tGcgCg (Figure 2). The opportunity for preferred interactions along the length of this 21-base target motif might stabilize a transposase-DNA pre-cleavage or cleavage complex for co-crystalization or other imaging studies.

## Conclusions

We have identified differences in the ability to tolerate transposon integrations between *Salmonella* Typhimurium strain 14028 and two strains of *S.* Typhi Ty2. We also found potential differences in essentiality of homologous genes between *Salmonella* and *E. coli* (summarized in Tables 2 and 3).

Sometimes, these differences in selection can be explained by the presence of an identifiable paralog, present in one genome but not another, which can take over some or all of the functions of the mutated gene. Examples include *trpS* and *holE*. In other cases, a difference in a function encoded elsewhere in the genome differentially impacts the role of orthologs. The best example of this phenomenon in the present study is *hns* which is essential in STM1 but not in either STY dataset. It is known that *rpoS* mutants permit mutations in *hns* in *Salmonella* in some circumstances [19], and STY1 and STY2 are *rpoS* mutants [17,18]. Similarly, *hnr* also showed a profound selection only in STM1. Although this gene is not essential in *Salmonella*, the presence of a functional copy of *rpoS* may also be the reason for this selection, because Hnr acts as a stability moderator for RpoS [22].

For some genes, such as *yejM*, *ftsN* and *murB,* we identified ORF segments that are essential whereas other regions of the gene can be freely interrupted by transposon insertions. Likely, these non-essential ORF regions exclude protein domains that are involved in critical functional modules of those genes.

Our experiments here involve comparisons among three *Salmonella* strains grown in rich media. However, the study of fitness profiles in hundreds of different strains in multiple growth conditions has the potential to reveal differences in life strategy not evident from the genome sequences alone and to contribute to understanding natural diversity. Advances in DNA sequencing and the ability to incorporate any number of different barcodes for comparison of multiple samples at the same time mean that comparative analysis of fitness among many different natural strains with different phenotypes has become practical. We speculate that as systems biology models become more refined, the fitness profiles of genomes may become useful for constraining these models.

## Methods

### Strains and growth conditions

The strains used in this study were *Salmonella enterica* serovar Typhimurium ATCC 14028 (STM1) and *S.* Typhi Ty2 strain JSG624 (STY1) provided by Ferric Fang (University of Washington, Seattle, WA). Bacterial cells were grown in LB medium containing 1% Bacto tryptone (Difco), 0.5% Bacto yeast extract (Becton, Dickinson and Company), and 1% NaCl, supplemented with kanamycin at 50 μg/ml, when necessary.

### Construction of transposon integrations in *S.* Typhimurium 14028 and *S.* Typhi Ty2

*Salmonella* cells were made competent by standard methodology. Briefly, cells were grown in LB with shaking at 37°C to logarithmic phase, then washed three times with cold 10% glycerol and concentrated 250 fold in 10% glycerol. Transposome mixtures were prepared mixing 2 μl glycerol, 2 μl EZ-Tn5 < T7/KAN-2 > transposon, and 4 μl EZ-Tn5 transposase. After 3 h of incubation, 1 μl of this mixture was mixed with 50 μl of competent cells and 1 μl TypeOne restriction inhibitor. Transformation was performed at 2.5 kV using 0.2 cm electrode gap cuvettes and a Bio-Rad MicroPulser at EC2 setting. Transformed cells in each cuvette were resuspended in 1 ml of LB and incubated for 1 h at 37°C. After incubation, reaction aliquots were joined, complexity was determined by cell counts of various dilutions on LB agar containing kanamycin, and the remainder was grown overnight at 37°C in LB broth supplemented with kanamycin. Stocks of the different transposon mutant libraries containing 20% glycerol were prepared from the overnight cultures.

### High-throughput sequencing of transposon insertion sites

The entire procedure is illustrated in Additional file 3: Figure S1. In brief, DNA was sheared, poly(A) tailed and PCR amplified using a pair of primers, one located in the transposon and one appended to the poly(A) tail, in a manner similar to that described in Santiviago *et al. *[4]. Subsequently, Illumina sequencing primers were added by PCR and sequencing performed on a Genome Analyzer GAII.

### Mapping of transposons to the genome

The beginning of each read primer contained a code that defined the particular transposon library used. The codes are listed in Additional file 1: Table S1. The reads were sorted into seven libraries. The sequencing primers were positioned such that the first two bases beyond the primers used for PCR would be the last two bases of the transposon. Reads that included this AG sequence were retained. Most transposons were represented by many shear events. Transposons that were represented by only one or two shear events were distributed throughout the genome, including in known essential regions. Thus, these rare reads were artifactual samples of the whole genome. These sites were eliminated from further consideration.

### Mapping of genes between genomes

Best hits between Typhimurium 14028, Typhimurium LT2, Typhi CT18, Typhi Ty2 and *E. coli* K-12 were identified by Blast searches of annotated genes against each other in the different genomes. Synteny was determined based on at least one of the two adjacent genes also being a best hit and being adjacent in other genomes. The assigned unique gene numbers differ for all the genome annotations, so all assigned gene numbers and gene symbols in these five genomes are reported in the Additional file 5: Table S2 for the convenience of the reader.

### Data processing for genome comparisons

Because the distribution and number of transposons is not identical between STM1 and STY1, the data were processed to express the ratio of the number of transposon integrations in each gene and intergenic regions versus the number of integrations in that region that would be expected if all the integrations were randomly distributed. The ratio of observed divided by expected numbers were then expressed as a log_2_. The published STY2 data [3] were recalculated to allow direct comparison.

To identify those genes that had large differences in fitness among STM1, STY1 and STY2, all log_2_ ratios (observed number of insertion events/expected number of insertion events) among all 3,907 genes shared between these genomes were ranked from those with the lowest density of transposons to those with the highest density. Ranks in STM1 and both STYs were subsequently subtracted to identify those genes with the biggest difference in rank order of transposon density.

The percentile rank of the number of transposons per base and the number of reads per base (shear events) were calculated for each putative ortholog in each experiment in *Salmonella*. We arbitrarily defined “selected” genes as those having a sum of the two percentile ranks less than 30% (i.e., averaging less than the 15^th^ percentile).

## Competing interests

The authors declare that they have no competing interests.

## Authors' contributions

RC constructed the transposon libraries, and developed the high-throughput sequencing strategy. She helped to write the manuscript and prepare the tables and figures. CF performed the sample preparation for sequencing. SWC coordinated and directed the sequencing project. XQX implemented the trimming and mapping strategy for counting transposons and shear events. He implemented the plots of transposon and shear events across the STM1 genome. BA provided four of the transposon libraries used for screening. HLAP advised on some steps of the project. SP was involved in Typhimurium annotation and manuscript writing. MM devised the sequencing and mapping strategies, helped to analyze the data, and wrote the manuscript. All authors read and approved the final manuscript.

## Supplementary Material

Additional file 1 **Table S1.** Transposon libraries (EZ-Tn5 < T7/Kan-2>) assayed. Click here for file

Additional file 2 **Supplemental Methods.** Detailed explanation of protocols. Click here for file

Additional file 3 **Figure S1.** Diagram of sequencing protocol. Click here for file

Additional file 4 **Figure S2.** Transposon insertion frequency across the *S.* Typhimurium 14028 s genome. Click here for file

Additional file 5 **Table S2.** Essentiality surveys and transposon data. Click here for file

Additional file 6 **Table S3.** STY1 complete transposon data. Click here for file

## References

[B1] Badarinarayana V Estep PW Shendure J Edwards J Tavazoie S Lam F Church GM Selection analyses of insertional mutants using subgenic-resolution arrays Nat Biotechnol 2001 19 11 1060 1065 10.1038/nbt1101-1060 11689852

[B2] Sassetti CM Boyd DH Rubin EJ Comprehensive identification of conditionally essential genes in mycobacteria Proc Natl Acad Sci U S A 2001 98 22 12712 12717 10.1073/pnas.231275498 11606763 PMC60119

[B3] Langridge GC Phan MD Turner DJ Perkins TT Parts L Haase J Charles I Maskell DJ Peters SE Dougan G Simultaneous assay of every *Salmonella* Typhi gene using one million transposon mutants Genome Res 2009 19 12 2308 2316 10.1101/gr.097097.109 19826075 PMC2792183

[B4] Santiviago CA Reynolds MM Porwollik S Choi SH Long F Andrews-Polymenis HL McClelland M Analysis of pools of targeted *Salmonella* deletion mutants identifies novel genes affecting fitness during competitive infection in mice PLoS Pathog 2009 5 7 e1000477 10.1371/journal.ppat.1000477 19578432 PMC2698986

[B5] Knuth K Niesalla H Hueck CJ Fuchs TM Large-scale identification of essential *Salmonella* genes by trapping lethal insertions Mol Microbiol 2004 51 6 1729 1744 10.1046/j.1365-2958.2003.03944.x 15009898

[B6] Thiele I Hyduke DR Steeb B Fankam G Allen DK Bazzani S Charusanti P Chen FC Fleming RM Hsiung CA A community effort towards a knowledge-base and mathematical model of the human pathogen *Salmonella* Typhimurium LT2 BMC Syst Biol 2011 5 8 10.1186/1752-0509-5-8 21244678 PMC3032673

[B7] Baba T Ara T Hasegawa M Takai Y Okumura Y Baba M Datsenko KA Tomita M Wanner BL Mori H Construction of *Escherichia coli* K-12 in-frame, single-gene knockout mutants: the Keio collection Mol Syst Biol 2006 2 2006 2008 10.1038/msb4100050 PMC1681482 16738554

[B8] Yamamoto N Nakahigashi K Nakamichi T Yoshino M Takai Y Touda Y Furubayashi A Kinjyo S Dose H Hasegawa M Update on the Keio collection of *Escherichia coli* single-gene deletion mutants Mol Syst Biol 2009 5 335 20029369 10.1038/msb.2009.92 PMC2824493

[B9] Hashimoto M Ichimura T Mizoguchi H Tanaka K Fujimitsu K Keyamura K Ote T Yamakawa T Yamazaki Y Mori H Cell size and nucleoid organization of engineered *Escherichia coli* cells with a reduced genome Mol Microbiol 2005 55 1 137 149 15612923 10.1111/j.1365-2958.2004.04386.x

[B10] Kato J Hashimoto M Construction of consecutive deletions of the *Escherichia coli* chromosome Mol Syst Biol 2007 3 132.11 17700540 10.1038/msb4100174 PMC1964801

[B11] McClelland M Sanderson KE Spieth J Clifton SW Latreille P Courtney L Porwollik S Ali J Dante M Du F Complete genome sequence of *Salmonella* enterica serovar Typhimurium LT2 Nature 2001 413 6858 852 856 10.1038/35101614 11677609

[B12] Hamilton S Bongaerts RJ Mulholland F Cochrane B Porter J Lucchini S Lappin-Scott HM Hinton JC The transcriptional programme of *Salmonella enterica* serovar Typhimurium reveals a key role for tryptophan metabolism in biofilms BMC Genomics 2009 10 599 10.1186/1471-2164-10-599 20003355 PMC2805695

[B13] Baek KT Vegge CS Skorko-Glonek J Brondsted L Different contributions of HtrA protease and chaperone activities to *Campylobacter jejuni* stress tolerance and physiology Appl Environ Microbiol 2011 77 1 57 66 10.1128/AEM.01603-10 21075890 PMC3019702

[B14] Cano DA Dominguez-Bernal G Tierrez A Garcia-Del Portillo F Casadesus J Regulation of capsule synthesis and cell motility in *Salmonella enterica* by the essential gene igaA Genetics 2002 162 4 1513 1523 12524328 10.1093/genetics/162.4.1513 PMC1462382

[B15] Costa CS Pettinari MJ Mendez BS Anton DN Null mutations in the essential gene *yrfF* (mucM) are not lethal in *rcsB*, yojN or *rcsC* strains of *Salmonella enterica* serovar Typhimurium FEMS Microbiol Lett 2003 222 1 25 32 10.1016/S0378-1097(03)00221-0 12757942

[B16] Garcia-Calderon CB Casadesus J Ramos-Morales F Regulation of igaA and the Rcs system by the MviA response regulator in *Salmonella enterica* J Bacteriol 2009 191 8 2743 2752 10.1128/JB.01519-08 19218385 PMC2668386

[B17] Robbe-Saule V Norel F The rpoS mutant allele of *Salmonella typhi* Ty2 is identical to that of the live typhoid vaccine Ty21a FEMS Microbiol Lett 1999 170 1 141 143 10.1111/j.1574-6968.1999.tb13366.x 9919662

[B18] Deng W Liou SR Plunkett G Mayhew GF Rose DJ Burland V Kodoyianni V Schwartz DC Blattner FR Comparative genomics of *Salmonella enterica* serovar Typhi strains Ty2 and CT18 J Bacteriol 2003 185 7 2330 2337 10.1128/JB.185.7.2330-2337.2003 12644504 PMC151493

[B19] Navarre WW Porwollik S Wang Y McClelland M Rosen H Libby SJ Fang FC Selective silencing of foreign DNA with low GC content by the H-NS protein in *Salmonella* Science 2006 313 5784 236 238 10.1126/science.1128794 16763111

[B20] Bearson SM Benjamin WH Swords WE Foster JW Acid shock induction of RpoS is mediated by the mouse virulence gene mviA of *Salmonella typhimurium* J Bacteriol 1996 178 9 2572 2579 8626324 10.1128/jb.178.9.2572-2579.1996 PMC177981

[B21] Benjamin WH Yother J Hall P Briles DE The Salmonella typhimurium locus *mviA* regulates virulence in Itys but not Ityr mice: functional *mviA* results in avirulence; mutant (nonfunctional) mviA results in virulence J Exp Med 1991 174 5 1073 1083 10.1084/jem.174.5.1073 1940789 PMC2119002

[B22] Zhou Y Gottesman S Hoskins JR Maurizi MR Wickner S The RssB response regulator directly targets sigma(S) for degradation by ClpXP Genes Dev 2001 15 5 627 637 10.1101/gad.864401 11238382 PMC312640

[B23] Chikova AK Schaaper RM The bacteriophage P1 hot gene product can substitute for the *Escherichia coli* DNA polymerase III theta subunit J Bacteriol 2005 187 16 5528 5536 10.1128/JB.187.16.5528-5536.2005 16077097 PMC1196078

[B24] Perkins TT Kingsley RA Fookes MC Gardner PP James KD Yu L Assefa SA He M Croucher NJ Pickard DJ Maskell DJ Parkhill J Choudhary J Thomson NR Dougan G A strand-specific RNA-Seq analysis of the transcriptome of the typhoid bacillus *Salmonella typhi* PLoS Genet 2009 5 7 e1000569 10.1371/journal.pgen.1000569 19609351 PMC2704369

[B25] Tacket CO Sztein MB Losonsky GA Wasserman SS Nataro JP Edelman R Pickard D Dougan G Chatfield SN Levine MM Safety of live oral *Salmonella typhi* vaccine strains with deletions in *htrA* and *aroC aroD* and immune response in humans Infect Immun 1997 65 2 452 456 9009296 10.1128/iai.65.2.452-456.1997 PMC174616

[B26] Hoiseth SK Stocker BA Aromatic-dependent *Salmonella typhimurium* are non-virulent and effective as live vaccines Nature 1981 291 5812 238 239 10.1038/291238a0 7015147

[B27] Hone DM Harris AM Chatfield S Dougan G Levine MM Construction of genetically defined double *aro* mutants of *Salmonella typhi* Vaccine 1991 9 11 810 816 10.1016/0264-410X(91)90218-U 1759503

[B28] Sebkova A Karasova D Crhanova M Budinska E Rychlik I aro mutations in *Salmonella enterica* cause defects in cell wall and outer membrane integrity J Bacteriol 2008 190 9 3155 3160 10.1128/JB.00053-08 18310348 PMC2347392

[B29] Johnson K Charles I Dougan G Pickard D O’Gaora P Costa G Ali T Miller I Hormaeche C The role of a stress-response protein in *Salmonella typhimurium* virulence Mol Microbiol 1991 5 2 401 407 10.1111/j.1365-2958.1991.tb02122.x 1645840

[B30] Rizzitello AE Harper JR Silhavy TJ Genetic evidence for parallel pathways of chaperone activity in the periplasm of *Escherichia coli* J Bacteriol 2001 183 23 6794 6800 10.1128/JB.183.23.6794-6800.2001 11698367 PMC95519

[B31] Skorko-Glonek J Sobiecka-Szkatula A Narkiewicz J Lipinska B The proteolytic activity of the HtrA (DegP) protein from *Escherichia coli* at low temperatures Microbiology 2008 154 Pt 12 3649 3658 19047732 10.1099/mic.0.2008/020487-0

[B32] Kuhle V Hensel M Cellular microbiology of intracellular *Salmonella enterica*: functions of the type III secretion system encoded by *Salmonella* pathogenicity island 2 Cell Mol Life Sci 2004 61 22 2812 2826 10.1007/s00018-004-4248-z 15558211 PMC11924503

[B33] Fass E Groisman EA Control of *Salmonella* pathogenicity island-2 gene expression Curr Opin Microbiol 2009 12 2 199 204 10.1016/j.mib.2009.01.004 19264535 PMC2805070

[B34] Ellermeier CD Ellermeier JR Slauch JM HilD, HilC and RtsA constitute a feed forward loop that controls expression of the SPI1 type three secretion system regulator *hilA* in *Salmonella enterica* serovar Typhimurium Mol Microbiol 2005 57 3 691 705 10.1111/j.1365-2958.2005.04737.x 16045614

[B35] Ellermeier CD Slauch JM RtsA and RtsB coordinately regulate expression of the invasion and flagellar genes in *Salmonella enterica* serovar Typhimurium J Bacteriol 2003 185 17 5096 5108 10.1128/JB.185.17.5096-5108.2003 12923082 PMC181000

[B36] Macnab RM How bacteria assemble Annu Rev Microbiol 2003 57 77 100 10.1146/annurev.micro.57.030502.090832 12730325

[B37] Macnab RM Type III flagellar protein export and flagellar assembly Biochim Biophys Acta 2004 1694 1–3 207 217 15546667 10.1016/j.bbamcr.2004.04.005

[B38] Karlinsey JE Tanaka S Bettenworth V Yamaguchi S Boos W Aizawa SI Hughes KT Completion of the hook-basal body of the *Salmonella typhimurium* flagellum is coupled to FlgM secretion and fliC transcription Mol Microbiol 2000 37 5 1220 1231 10.1046/j.1365-2958.2000.02081.x 10972838

[B39] Hirano T Minamino T Namba K Macnab RM Substrate specificity classes and the recognition signal for *Salmonella* type III flagellar export J Bacteriol 2003 185 8 2485 2492 10.1128/JB.185.8.2485-2492.2003 12670972 PMC152621

[B40] Paul K Erhardt M Hirano T Blair DF Hughes KT Energy source of flagellar type III secretion Nature 2008 451 7177 489 492 10.1038/nature06497 18216859

[B41] Minamino T Ferris HU Moriya N Kihara M Namba K Two parts of the T3S4 domain of the hook-length control protein FliK are essential for the substrate specificity switching of the flagellar type III export apparatus J Mol Biol 2006 362 5 1148 1158 10.1016/j.jmb.2006.08.004 16949608

[B42] Barker CS Meshcheryakova IV Kostyukova AS Samatey FA FliO regulation of FliP in the formation of the *Salmonella enterica* flagellum PLoS Genet 2010 6 9 pii:e1001143 10.1371/journal.pgen.1001143 PMC2947984 20941389

[B43] Aldridge C Poonchareon K Saini S Ewen T Soloyva A Rao CV Imada K Minamino T Aldridge PD The interaction dynamics of a negative feedback loop regulates flagellar number in *Salmonella enterica* serovar Typhimurium Mol Microbiol 2010 78 6 1416 1430 10.1111/j.1365-2958.2010.07415.x 21143315

[B44] Stafford GP Hughes C *Salmonella typhimurium flhE*, a conserved flagellar regulon gene required for swarming Microbiology 2007 153 Pt 2 541 547 17259626 10.1099/mic.0.2006/002576-0 PMC2528295

[B45] Wang Q Frye JG McClelland M Harshey RM Gene expression patterns during swarming in *Salmonella typhimurium*: genes specific to surface growth and putative new motility and pathogenicity genes Mol Microbiol 2004 52 1 169 187 10.1111/j.1365-2958.2003.03977.x 15049819

[B46] Becker D Selbach M Rollenhagen C Ballmaier M Meyer TF Mann M Bumann D Robust *Salmonella* metabolism limits possibilities for new antimicrobials Nature 2006 440 7082 303 307 10.1038/nature04616 16541065

[B47] Lodge JK Weston-Hafer K Berg DE Transposon Tn5 target specificity: preference for insertion at G/C pairs Genetics 1988 120 3 645 650 2852135 10.1093/genetics/120.3.645 PMC1203542

[B48] Goryshin IY Miller JA Kil YV Lanzov VA Reznikoff WS Tn5/IS50 target recognition Proc Natl Acad Sci U S A 1998 95 18 10716 10721 10.1073/pnas.95.18.10716 9724770 PMC27961

