# Supplementary material for: High-throughput comparison of gene fitness among related bacteria
Source: BMC Genomics. 2012 May 30;13:212. doi: 10.1186/1471-2164-13-212 (PMC3487940; doi:10.1186/1471-2164-13-212)
Supplement: Additional file 1 — Table S1. Transposon libraries (EZ-Tn5 < T7/Kan-2>) assayed. [file 1471-2164-13-212-S1.docx]

**Supplemental Table S1.** Transposon libraries (EZ-Tn5 <T7/Kan-2>) assayed.

| **Library** | **Species** | **Strain** | **Complexity** | **Primer name^a^** | **Primer sequence^a^** |
| --- | --- | --- | --- | --- | --- |
| L01 | *S*. Typhimurium | 14028^b^ | 39,000 | 2EZGTCA-2 | 5’*ACACTCTTTCCCTACACGACGCTCTTCCGATCT*GTCAAG**ATGTGTATAAGAGAC** |
| L02 | *S*. Typhimurium | 14028^b^ | 197,400 | 2EZGCCG-2 | 5’*ACACTCTTTCCCTACACGACGCTCTTCCGATCT*GCCGAG**ATGTGTATAAGAGAC** |
| L03 | *S*. Typhimurium | 14028 mutant in a neutral position (chloramphenicol resistant)^b^ | 90,000 | 2EZACTG-2 | 5’*ACACTCTTTCCCTACACGACGCTCTTCCGATCT*ACTGAG**ATGTGTATAAGAGAC** |
| L06 | *S*. Typhimurium | 14028 *invA*- mutant (chloramphenicol resistant)^b^ | 70,000 | 2EZATTA-2 | 5’*ACACTCTTTCCCTACACGACGCTCTTCCGATCT*ATTAAG**ATGTGTATAAGAGAC** |
| L10 | *S*. Typhimurium | 14028^b^ | 60,000 | 2EZCAGA-2 | 5’*ACACTCTTTCCCTACACGACGCTCTTCCGATCT*GTCAGA**ATGTGTATAAGAGAC** |
| L08 | *S*. Typhi | Ty2^c^ | 30,000 | 2EZGCTA-2 | 5’*ACACTCTTTCCCTACACGACGCTCTTCCGATCT*GCTAAG**ATGTGTATAAGAGAC** |
| L09 | *S*. Typhi | Ty2^c^ | 16,000 | 2EZATCG-2 | 5’*ACACTCTTTCCCTACACGACGCTCTTCCGATCT*ATCGAG**ATGTGTATAAGAGAC** |

^a^ Forward indexing primers used in the third PCR of the protocol for the high-throughput screening of the transposon libraries. The Illumina sequencing region is shown in italics, the N_6_ code is underlined and the transposon sequence is in bold.

^b^ All these strains are abbreviated as STM1 to show the global results for the five libraries.

^c^ These two strains are abbreviated as STY1.
