# Supplementary material for: High-throughput comparison of gene fitness among related bacteria
Source: BMC Genomics. 2012 May 30;13:212. doi: 10.1186/1471-2164-13-212 (PMC3487940; doi:10.1186/1471-2164-13-212)
Supplement: Additional file 2 — Supplemental Methods. Detailed explanation of protocols. [file 1471-2164-13-212-S2.docx]

**Supplemental Methods**

**1. DNA purification, fragmentation, and poly(A)-tailing**

The seven *Salmonella* transposon libraries were grown separately in LB supplemented with kanamycin overnight at 37 ^o^C with shaking. Genomic DNA of each library was isolated using the GenElute Bacterial Genomic DNA kit (Sigma-Aldrich, St Louis, MO) and 9 µg were fragmented by sonication performing five-second pulses in a Branson sonifier (model S-150D; Branson Ultrasonics Corporation, Danbury, CT). After denaturation at 95 ^o^C for 2 min, 1.6 µg of the sheared DNA were poly-A tailed using 40 U of terminal transferase (TdT, New England BioLabs) in 1x TdT buffer, 0.25 mM CoCl_2_, 0.4 mM dATP and incubation for 30 min at 37 ^o^C. The terminal tranferase was heat inactivated at 70 ^o^C for 10 min and the polyadenylated products were purified using the QIAquick PCR purification kit (Qiagen).

**2. PCR amplification, indexing, and size selection**

To amplify the *Salmonella* fragments adjacent to the transposons, a nested PCR was performed. In a first PCR reaction, 500 ng of the purified polyA-tailed DNA were used as a template, adding a transposon-specific forward primer, DOPR2 (CAACGCAGACCGTTCCGTGGCA) and a polyT reverse primer (CCT_24_VN). The PCR reaction (50 µl) was performed in 1x buffer (including 2 mM MgCl_2_), 0.2 mM dNTP, 0.05 U Taq polymerase (Monserate Biotechnology Group, San Diego, CA), and 0.2 mM of each primer. Cycling conditions consisted of an initial denaturation step at 94 ^o^C for 1 min, 30 cycles of denaturation at 94 ^o^C for 10 s, annealing at 50 ^o^C for 10 s, and extension at 72 ^o^C for 5 s, followed by a final extension for 3 min at 72 ^o^C. In a second PCR, 2 µl of the amplified product from the initial PCR were used as a template following the same PCR reaction conditions and using an internal transposon-specific forward primer, KAN2FP1-B (GTCCACCTACAACAAAGCTCTCATCAACC) and the CCT_24_VN primer. Successful amplification of products was verified by eletrophoresis in ethidium bromide stained 1 % agarose gels.

A third PCR reaction introduced a specific code to each library and the Illumina adapter sequences. 2 µl of the previous nested PCR were used, with the reverse primer 1EZTTTT (CTCGGCATTCCTGCTGAACCGCTCTTCCGATCT_23_V), designed to anneal to the polyA-tail. Forward indexing primers were designed to anneal to 15 bases at the 3´end of the transposon, not including the last two bases (AG). A specific N_6_ code was also introduced for each library (Additional file 1 Table S1). The PCR conditions and mixture were the same as in the previous PCR. The QIAquick PCR purification kit (Qiagen) was used to purify the PCR products and the indexed libraries were pooled in equimolar ratios (10 nM).

To introduce the Illumina engraftment sequences, a fourth PCR was performed for ten cycles using the custom Illumina primers PE PCR Primer 1.0 and PE PCR Primer 2.0 according to the manufacturer’s instructions. Excess primers were removed via AMPure magnetic bead cleanup (Agencourt Bioscience Corporation) according to manufacturer’s instructions, and the products were size-selected using polyacrylamide gel electrophoresis (PAGE), selecting products in the size range of 200-450 bp to be excised and purified from the gel using the QIAquick gel extraction kit (Qiagen).

**3. Library sequencing**

*Salmonella* libraries were sequenced using a single lane, paired-end reads and 101 cycles, of an Illumina Genome Analyzer II (GAII) sequencing system and the custom Illumina PE Read 1 and Read 2 Sequencing Primers. Paired-end reads were only performed because of availability of that particular lane in a sequencer run, to avoid further delay. Sequencing was performed at the Genome Institute at Washington University, St. Louis, MO.
