# Supplementary figures and images for: High-throughput comparison of gene fitness among related bacteria
Source: BMC Genomics. 2012 May 30;13:212. doi: 10.1186/1471-2164-13-212 (PMC3487940; doi:10.1186/1471-2164-13-212)

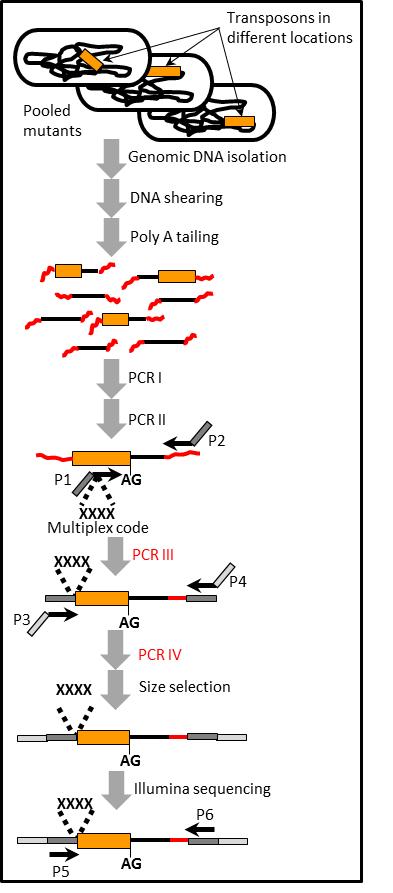


**Supplemental Figure S1.** High-throughput screening of transposon libraries.

Supplement: Additional file 3 — Figure S1. Diagram of sequencing protocol. [file 1471-2164-13-212-S3.docx]

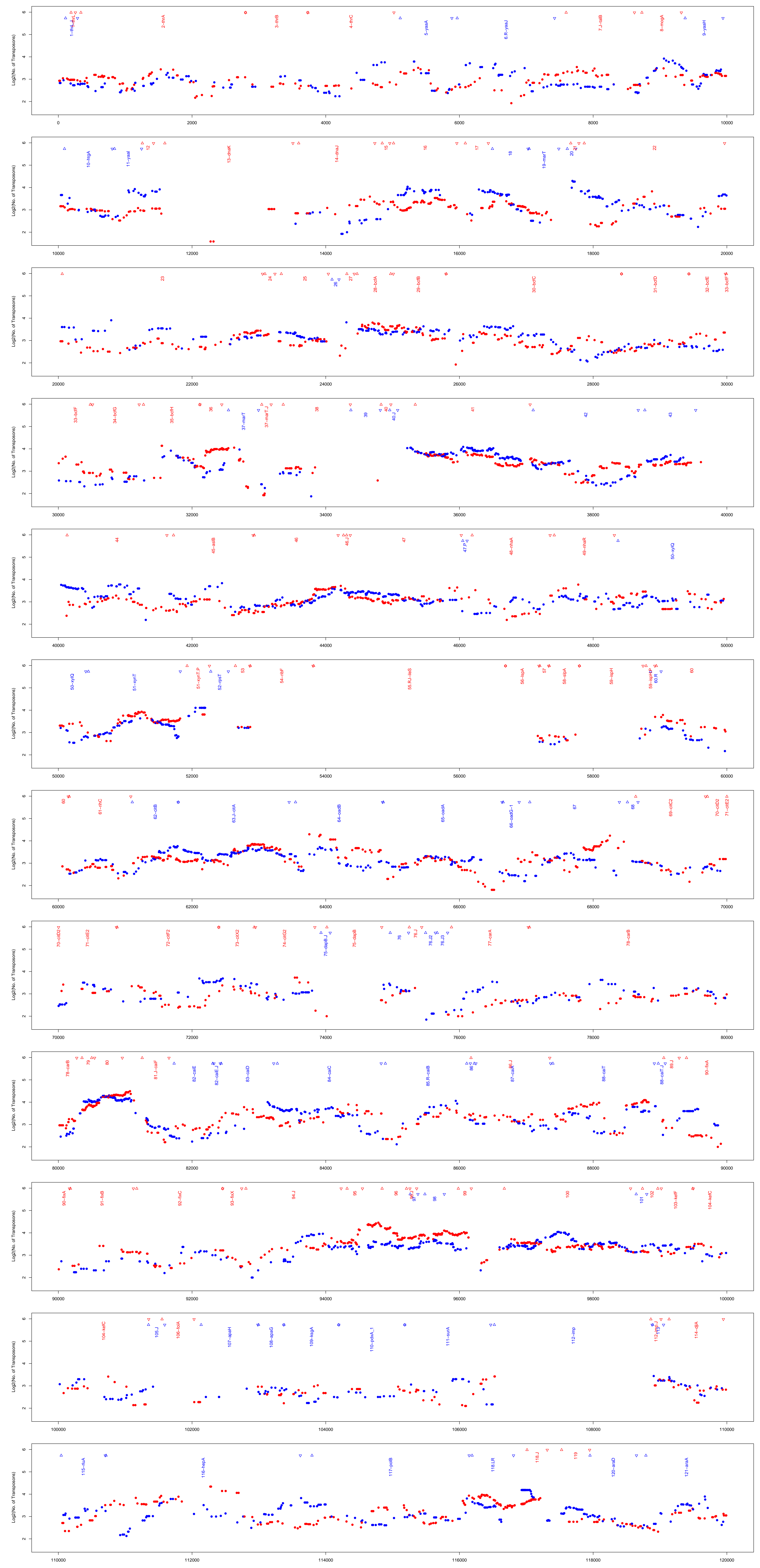

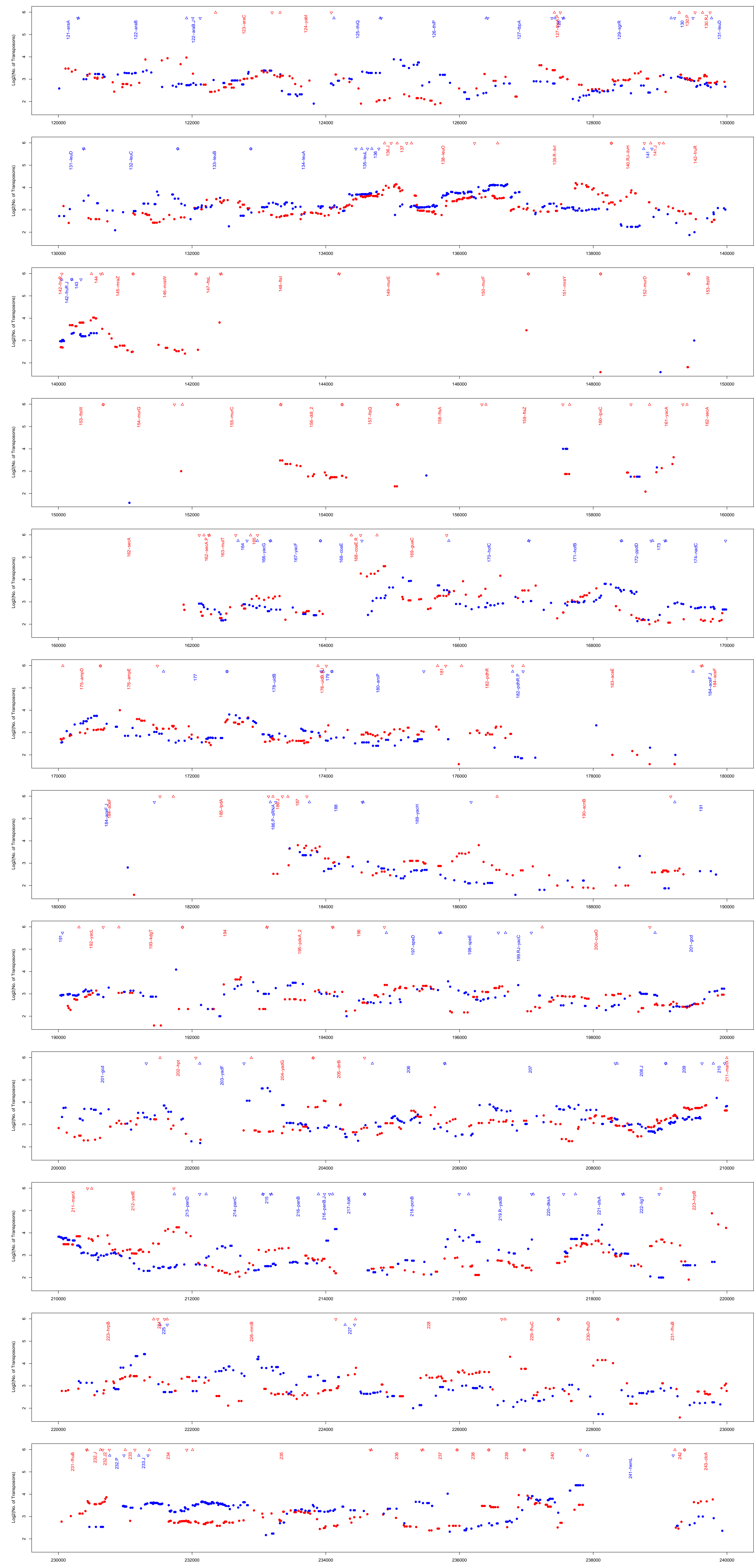

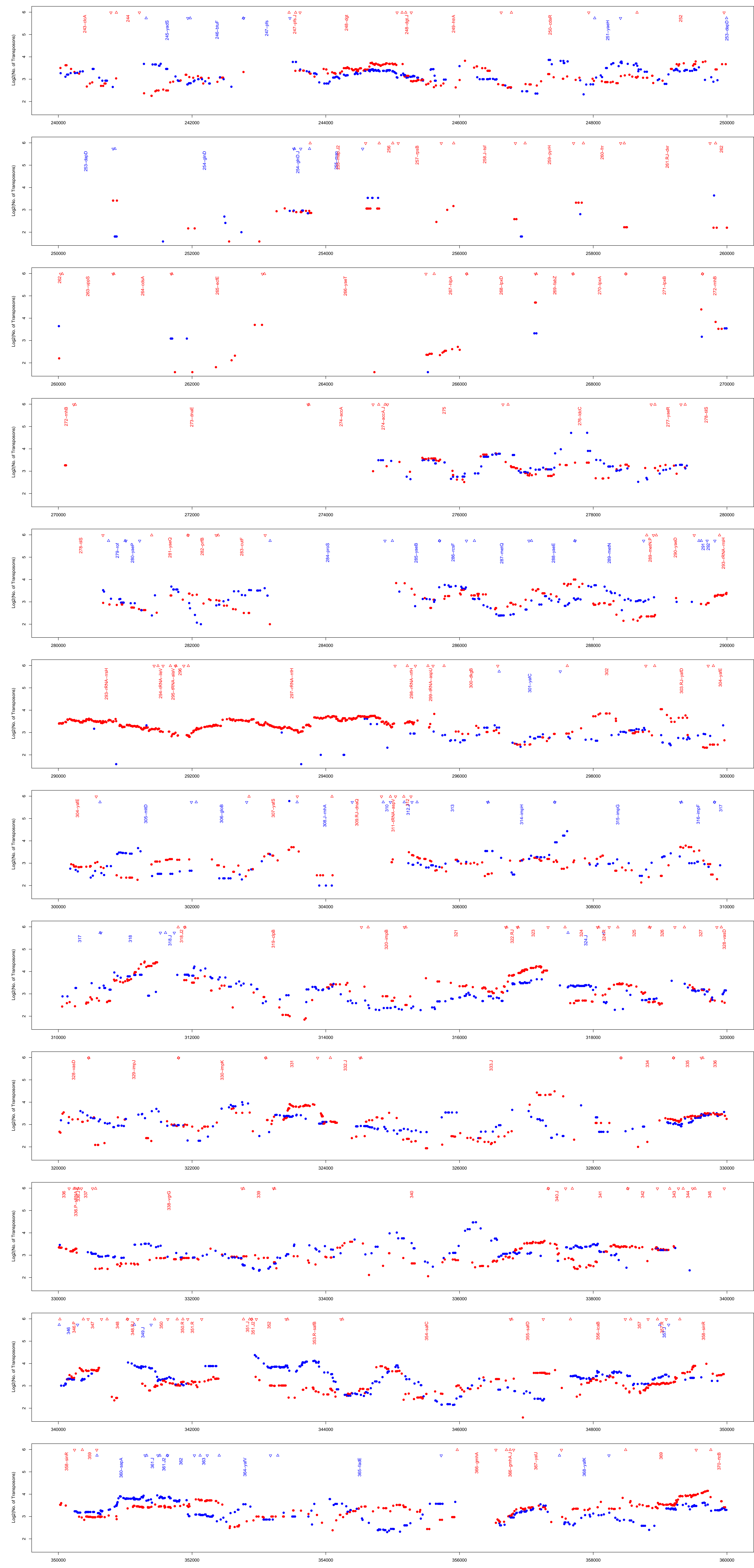

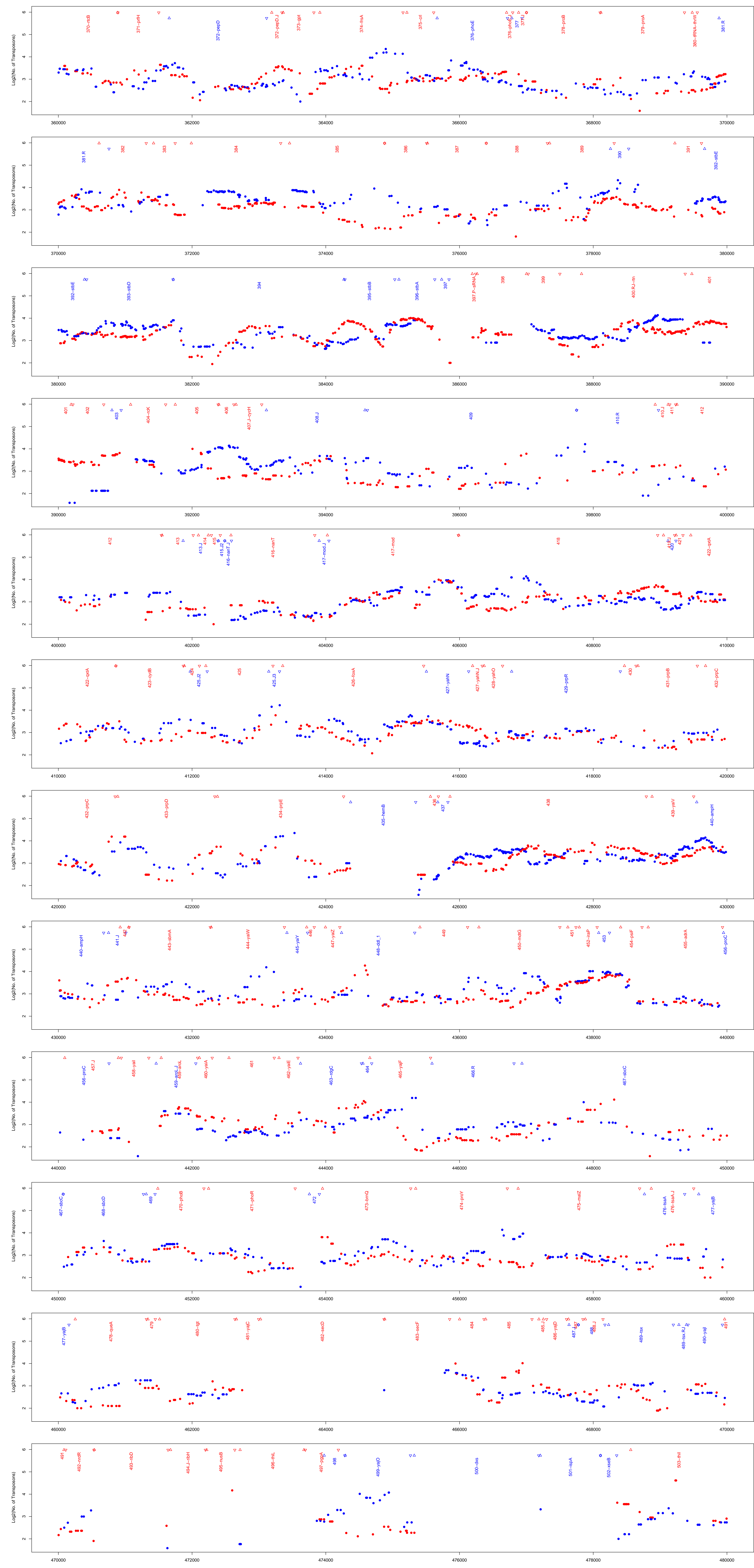

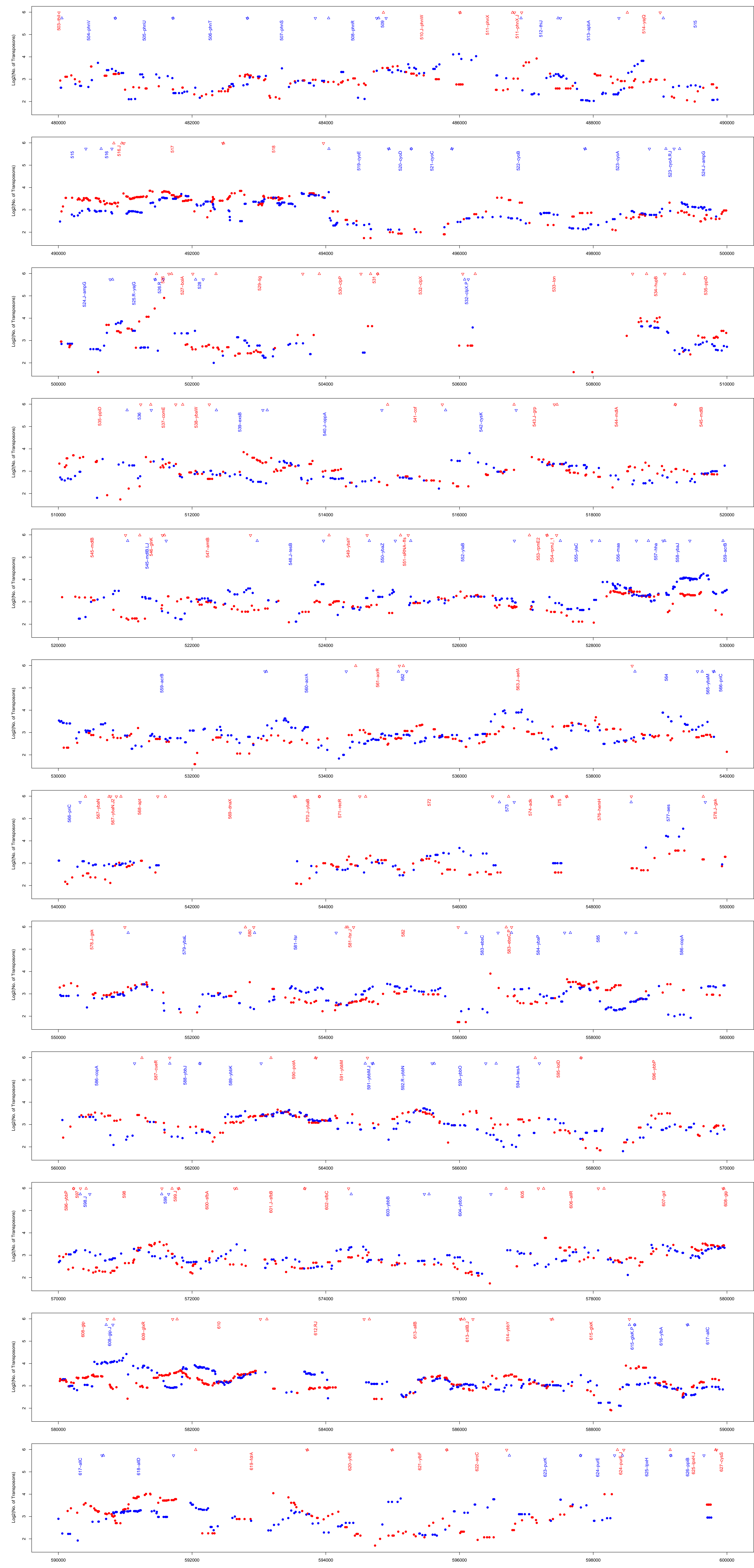

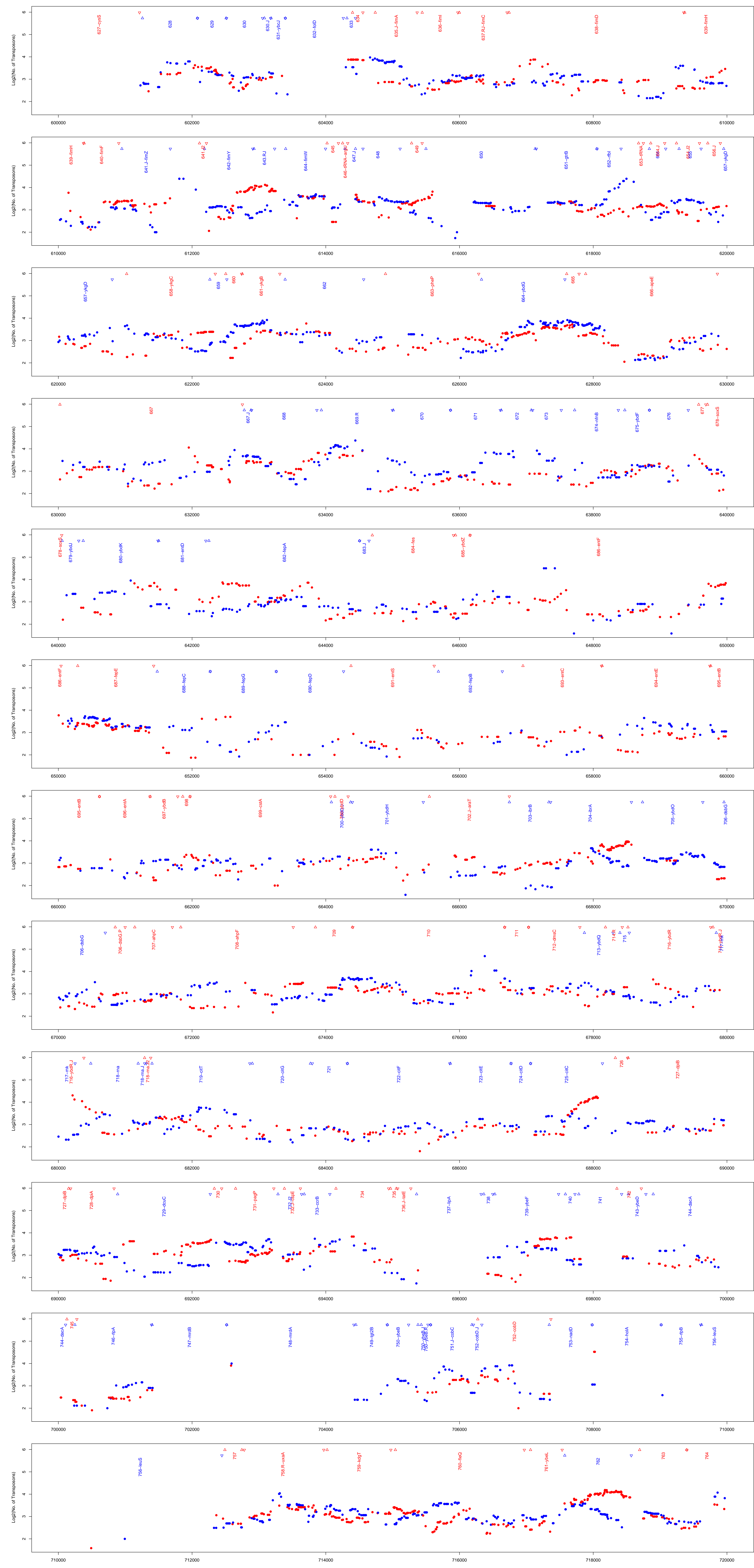

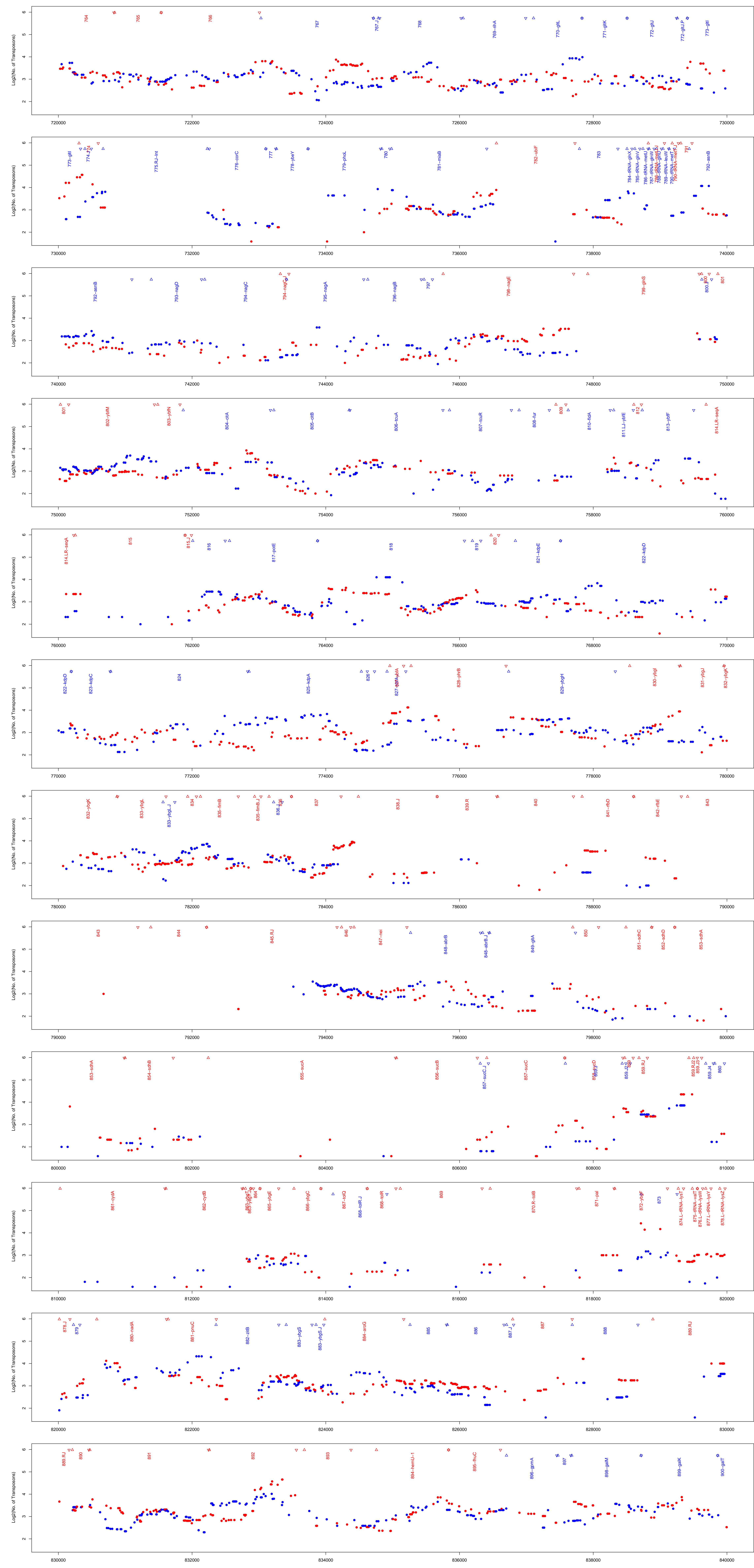

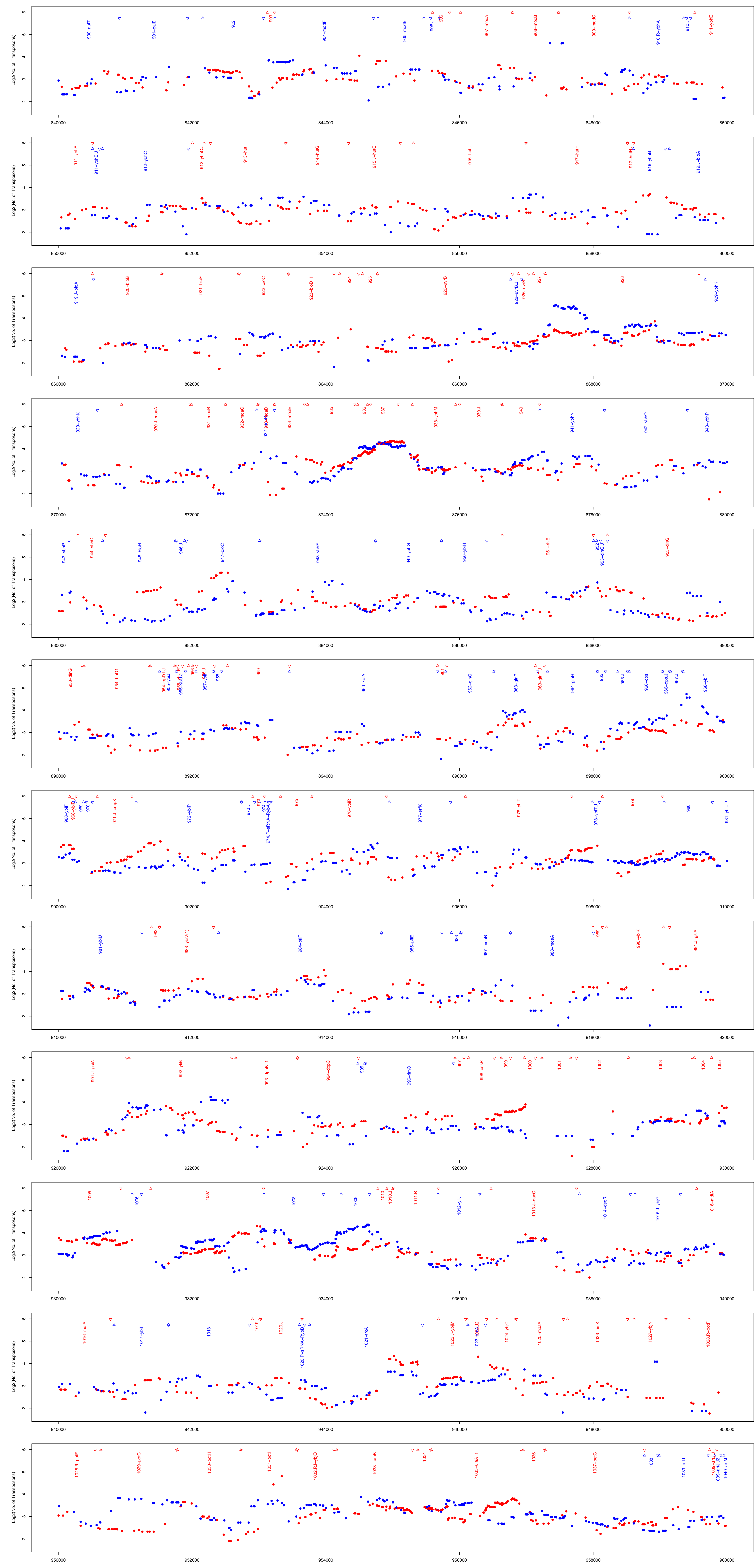

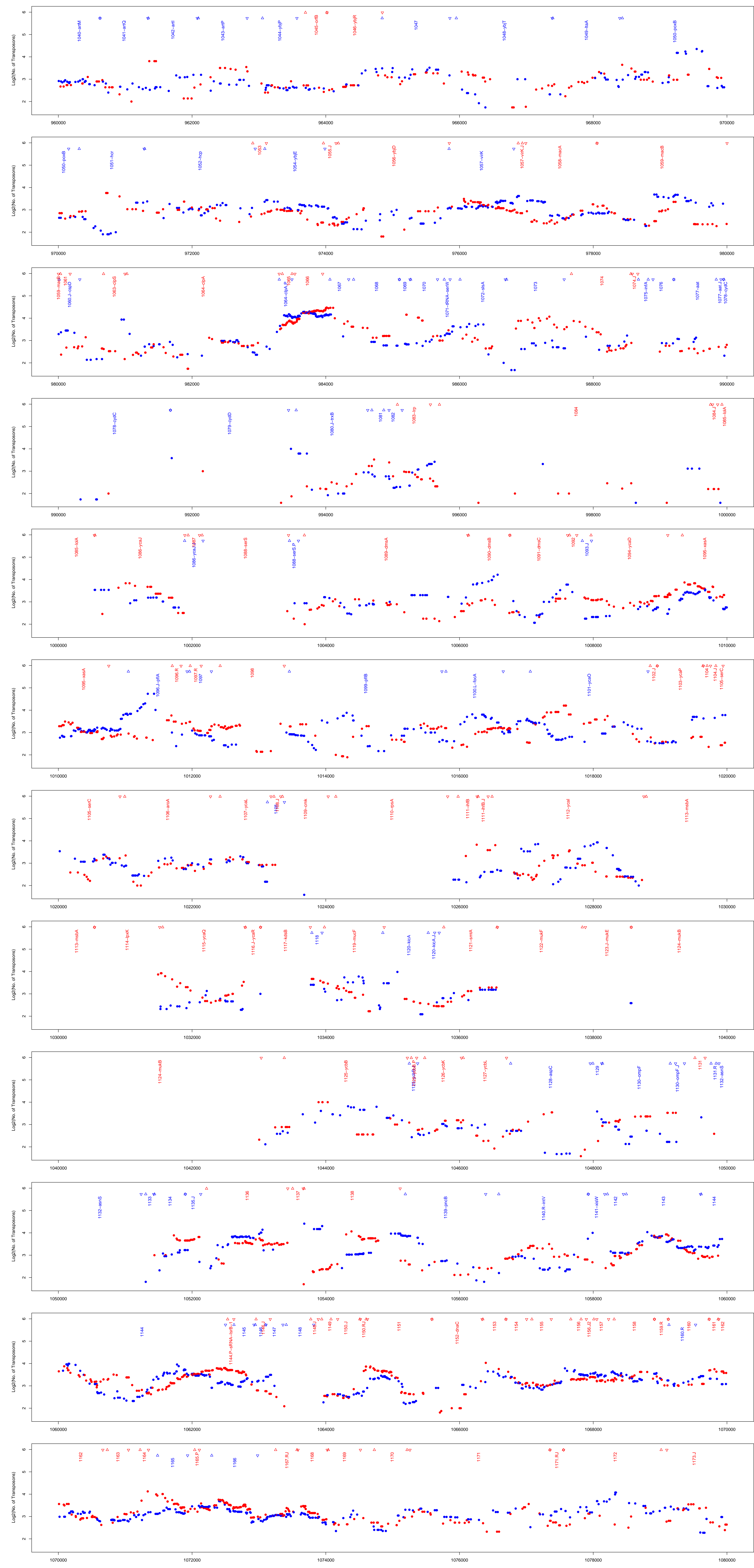

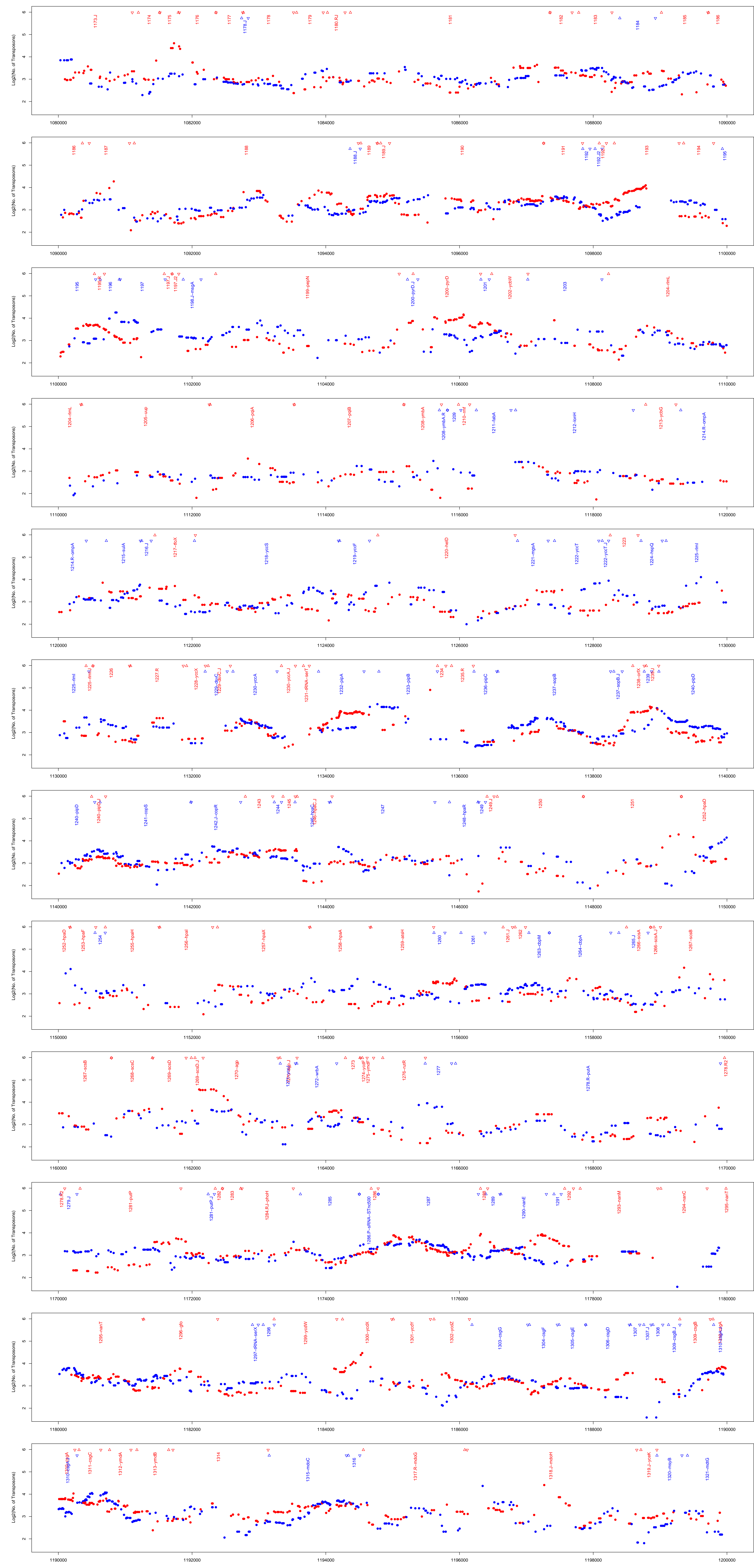

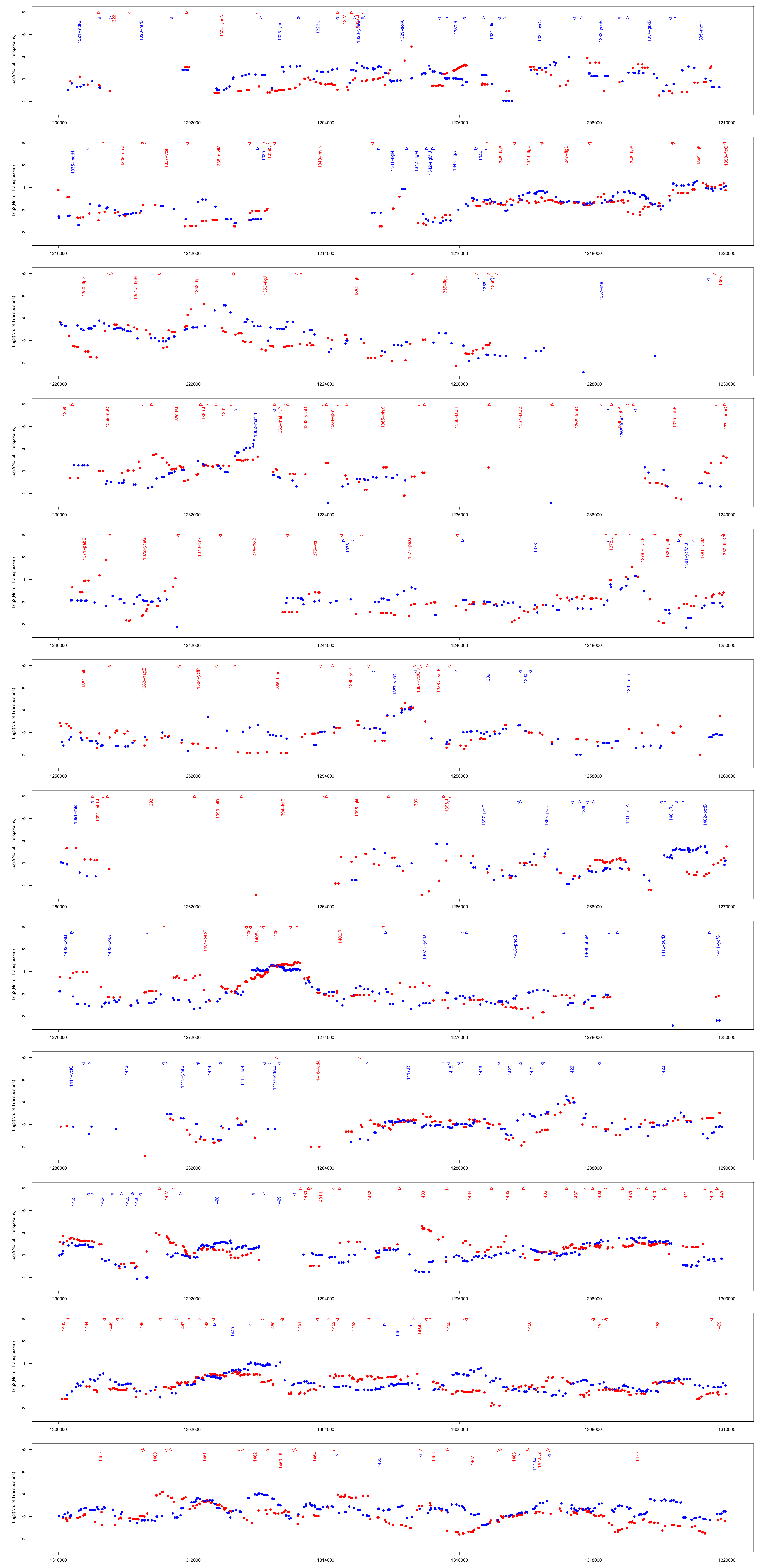

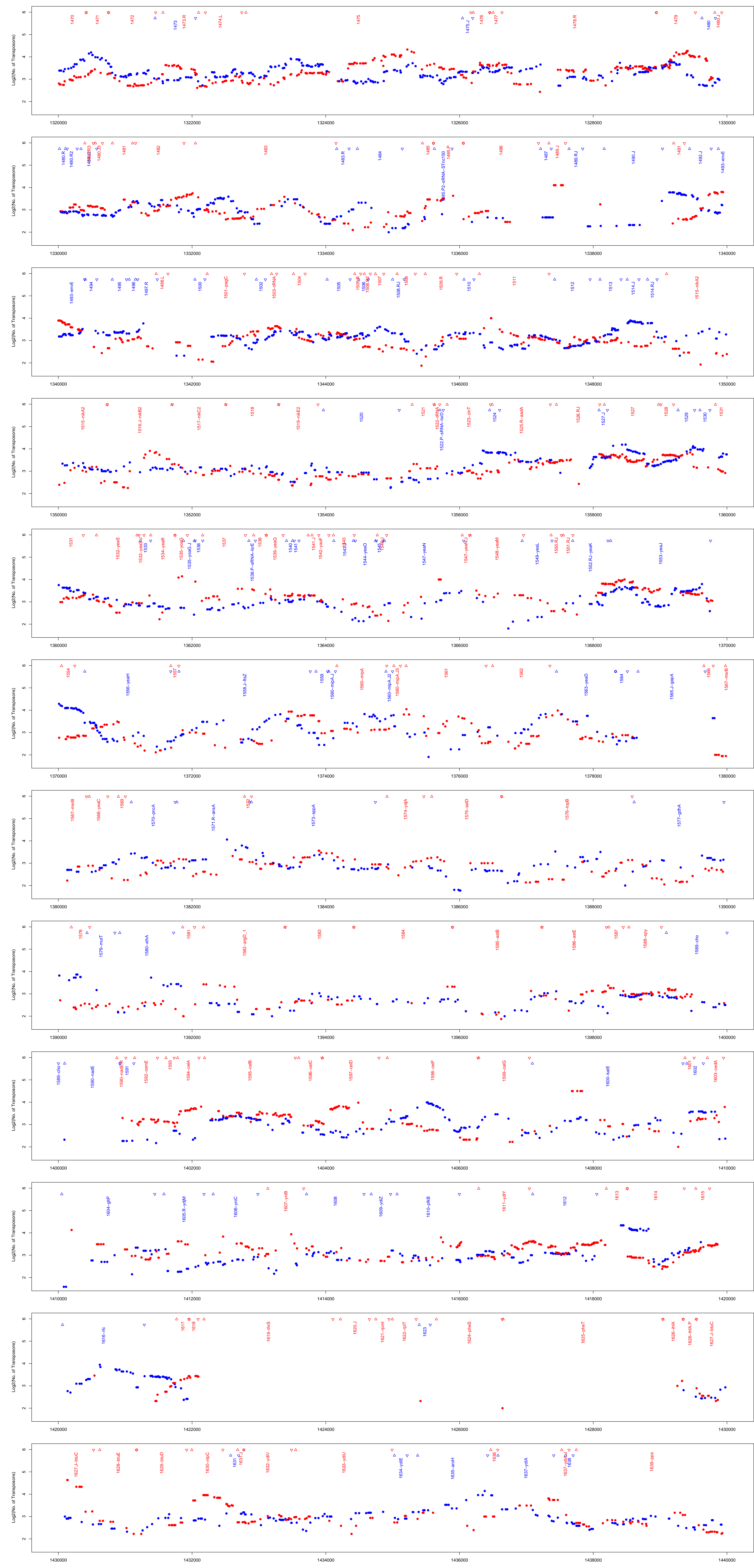

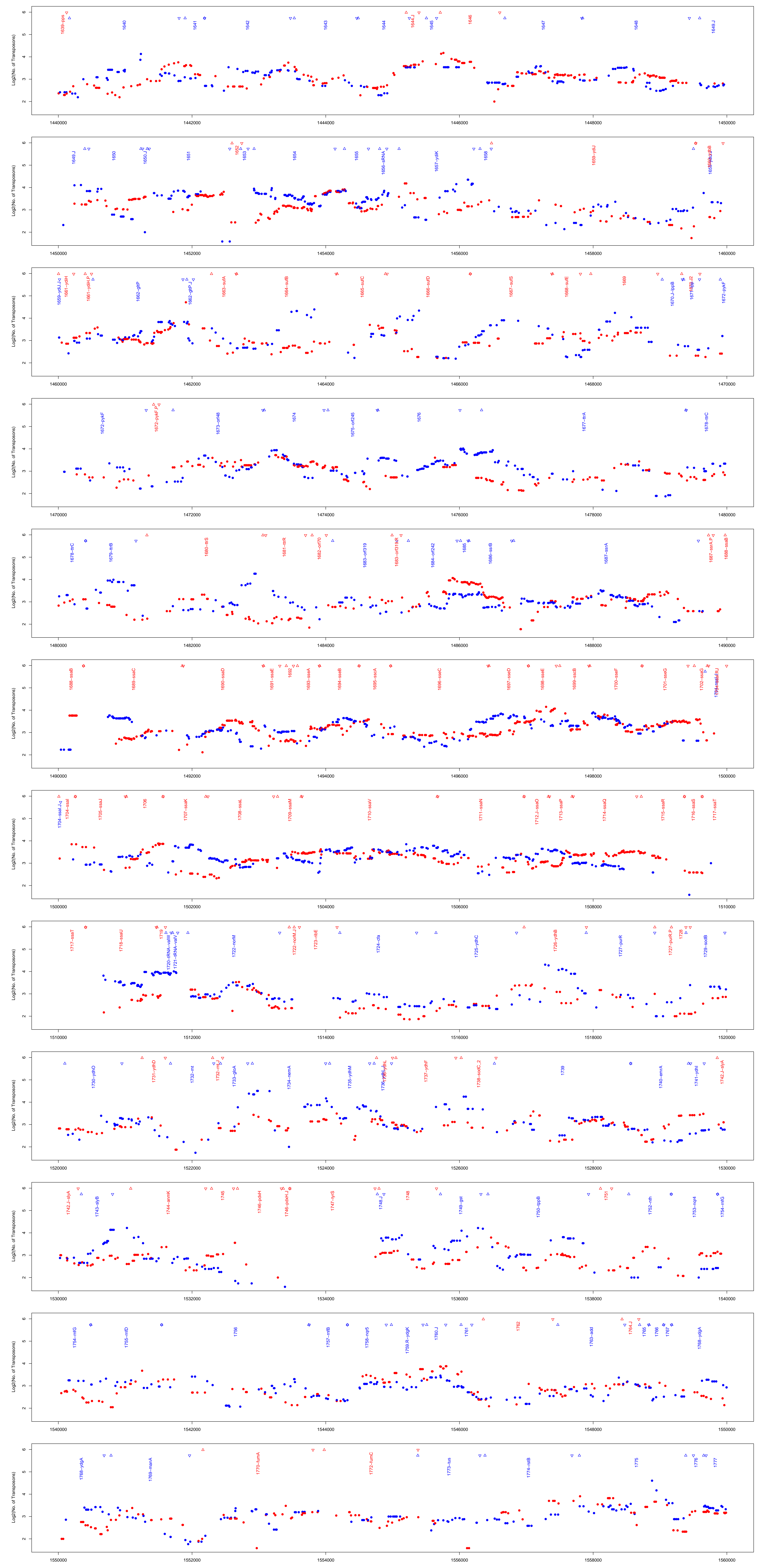

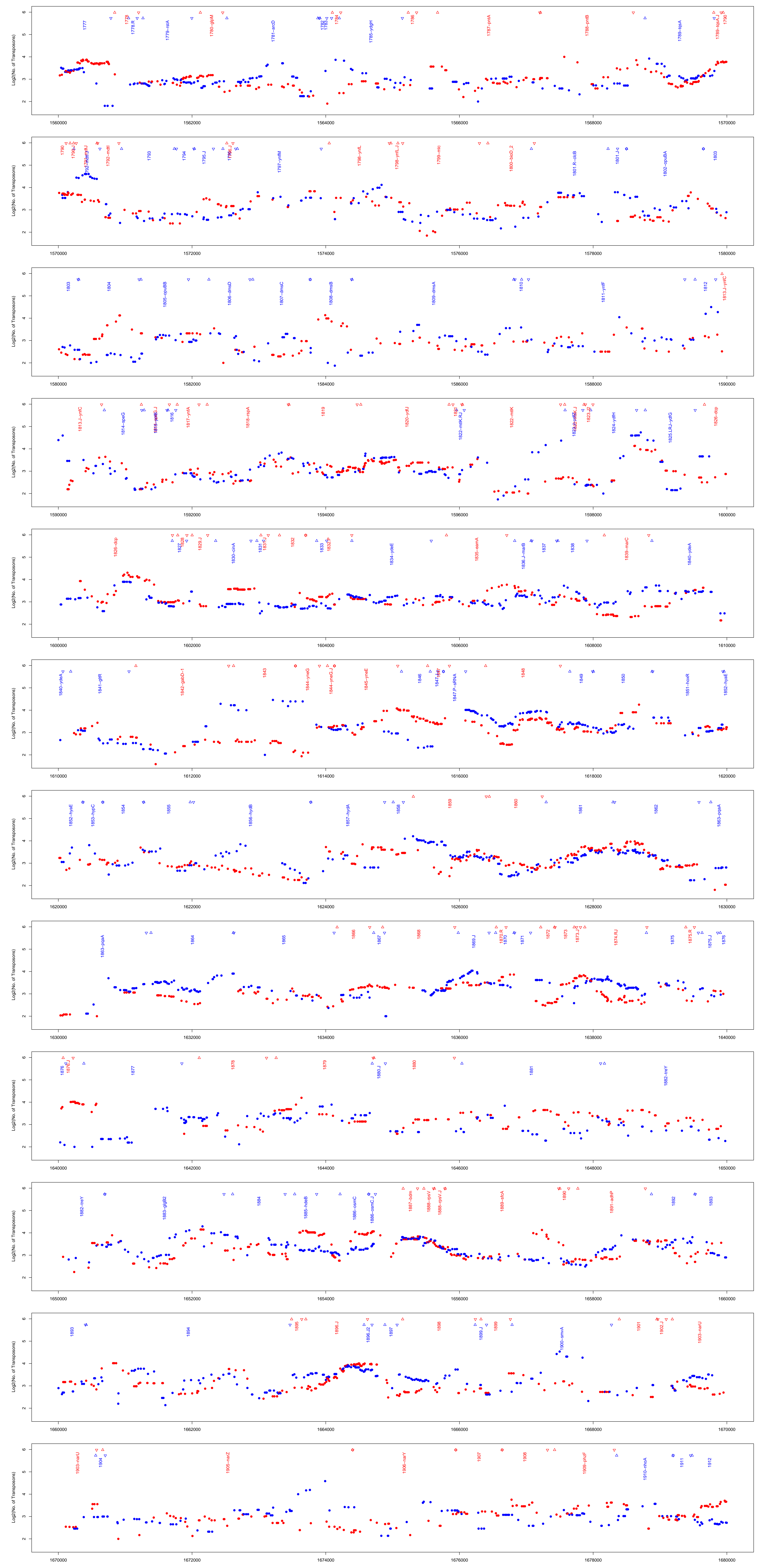

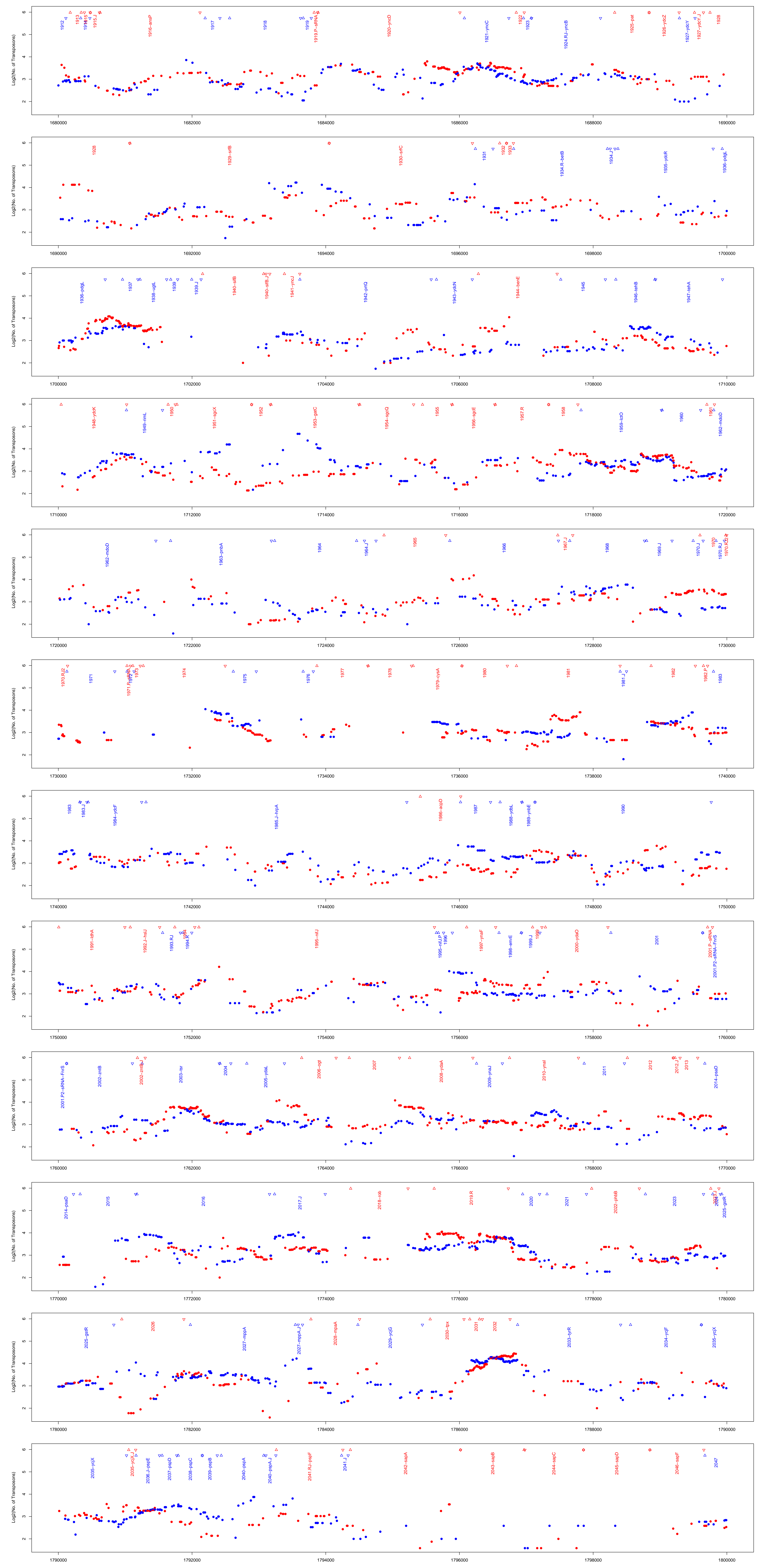

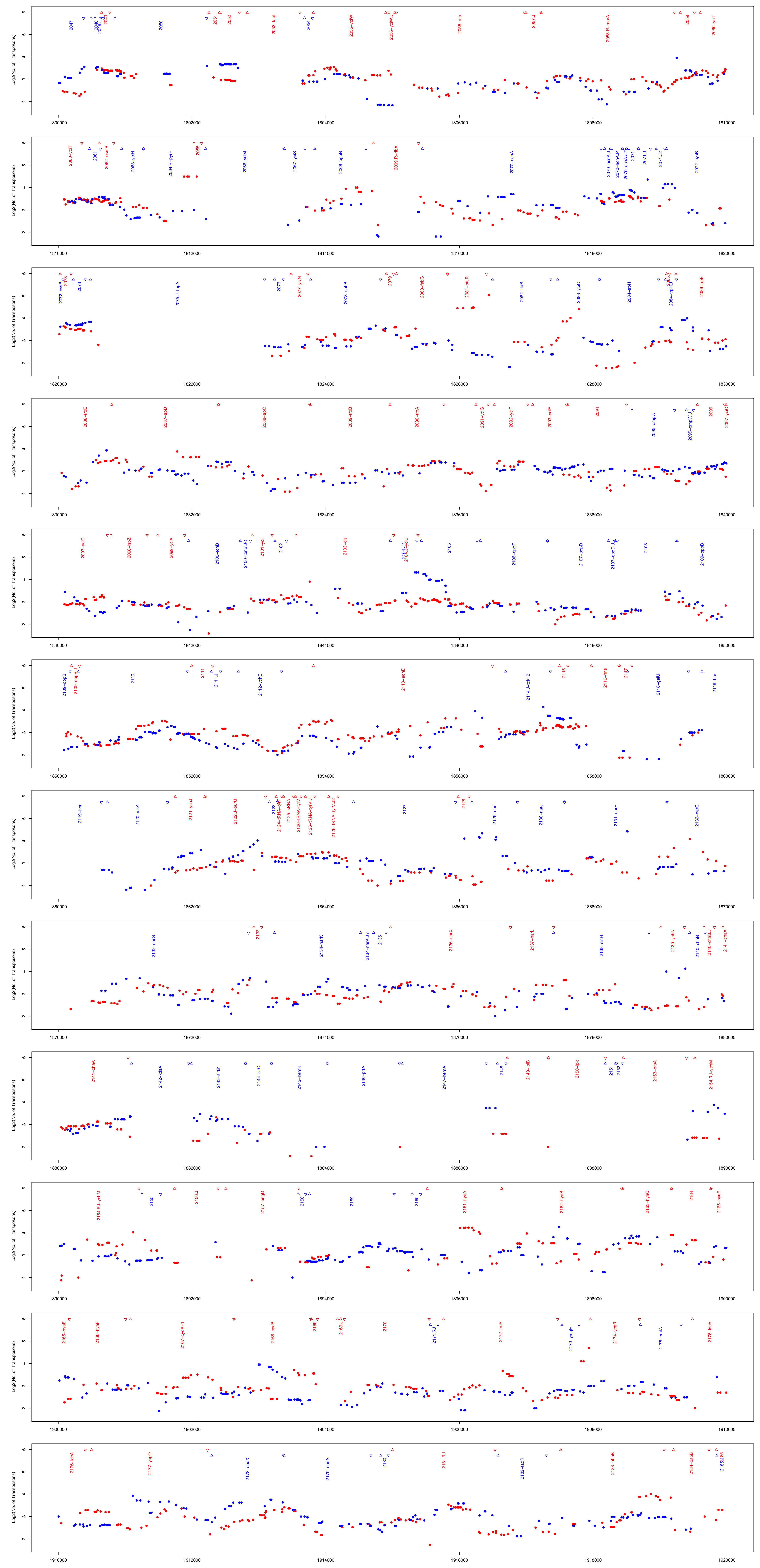

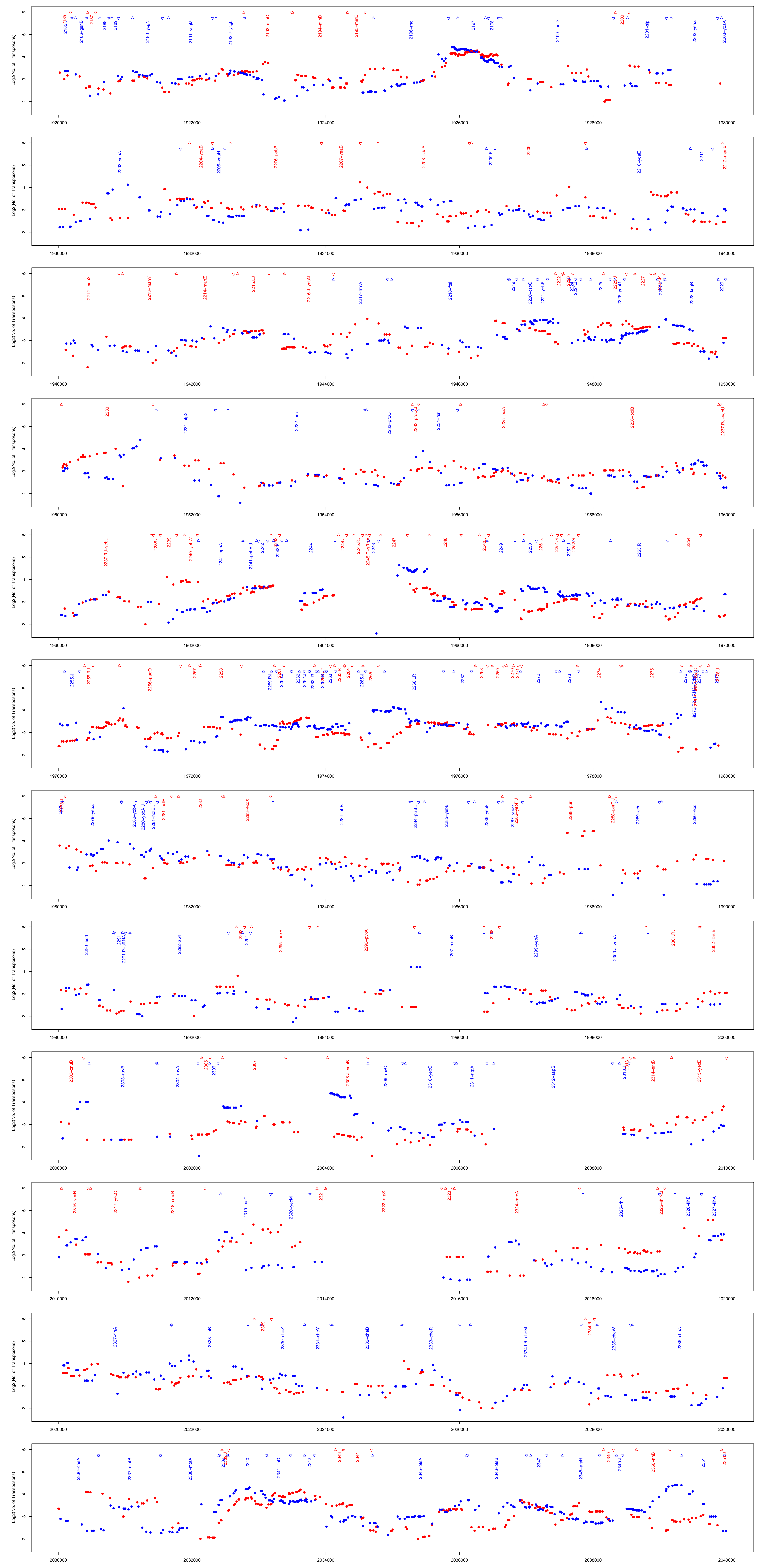

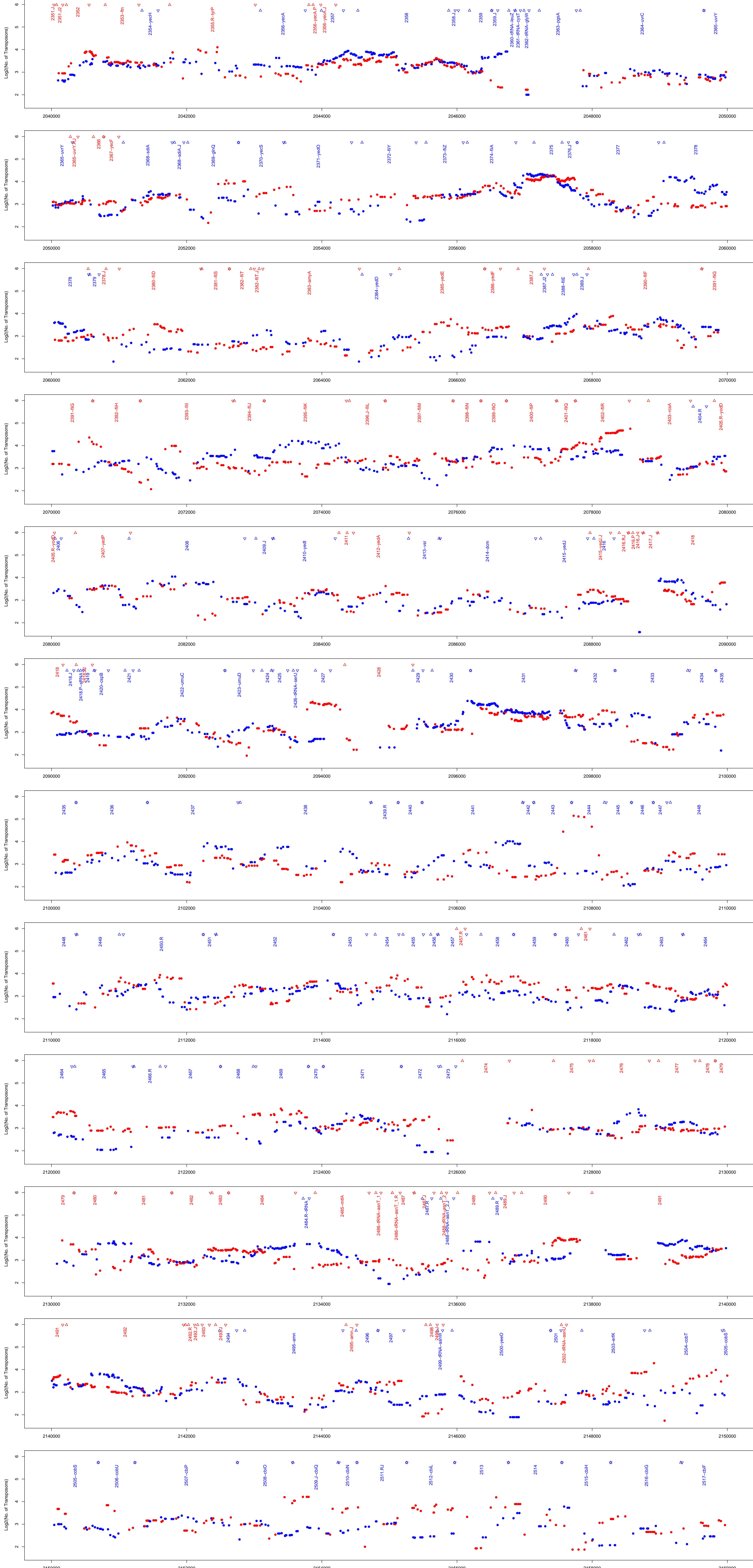

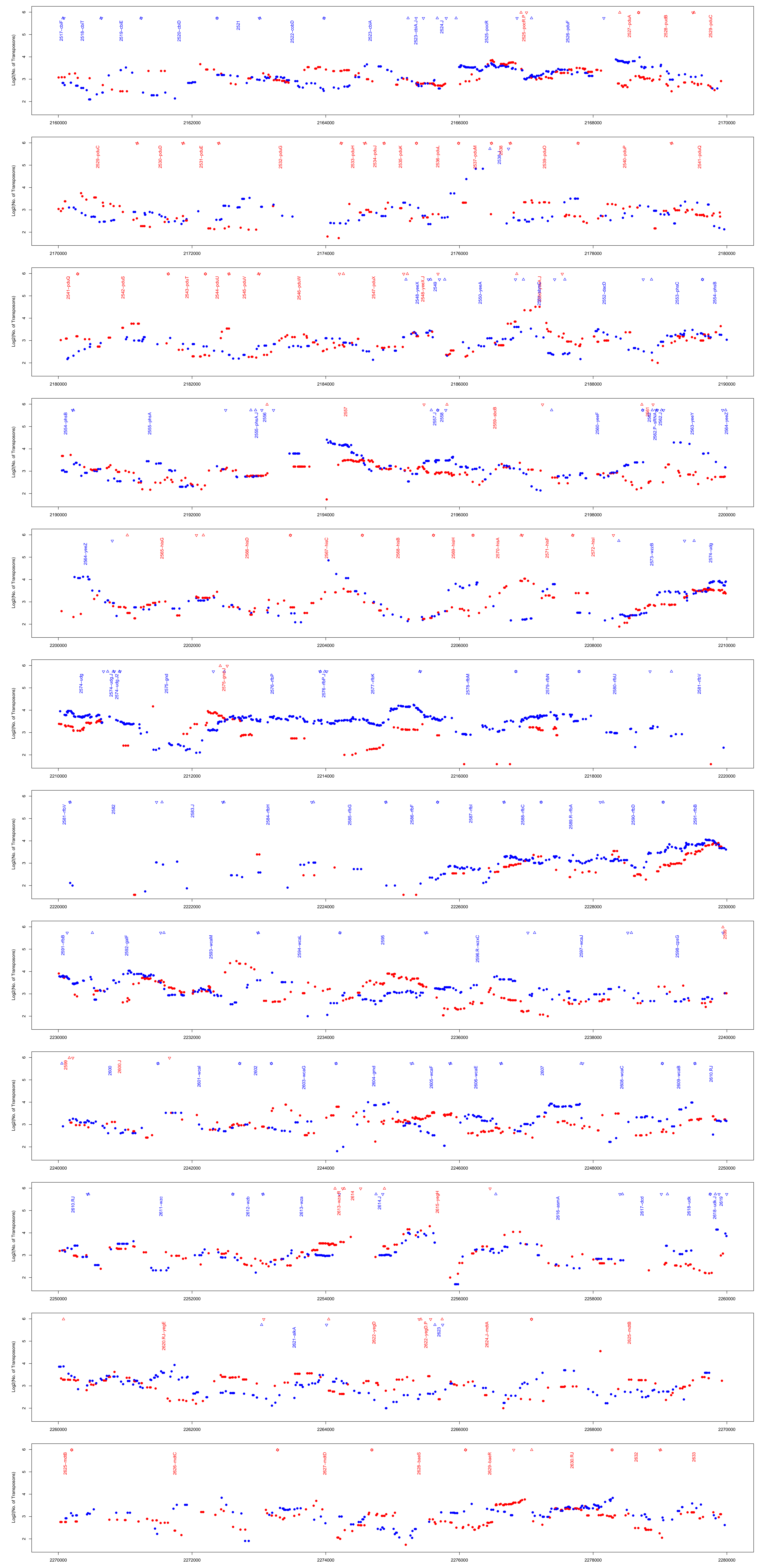

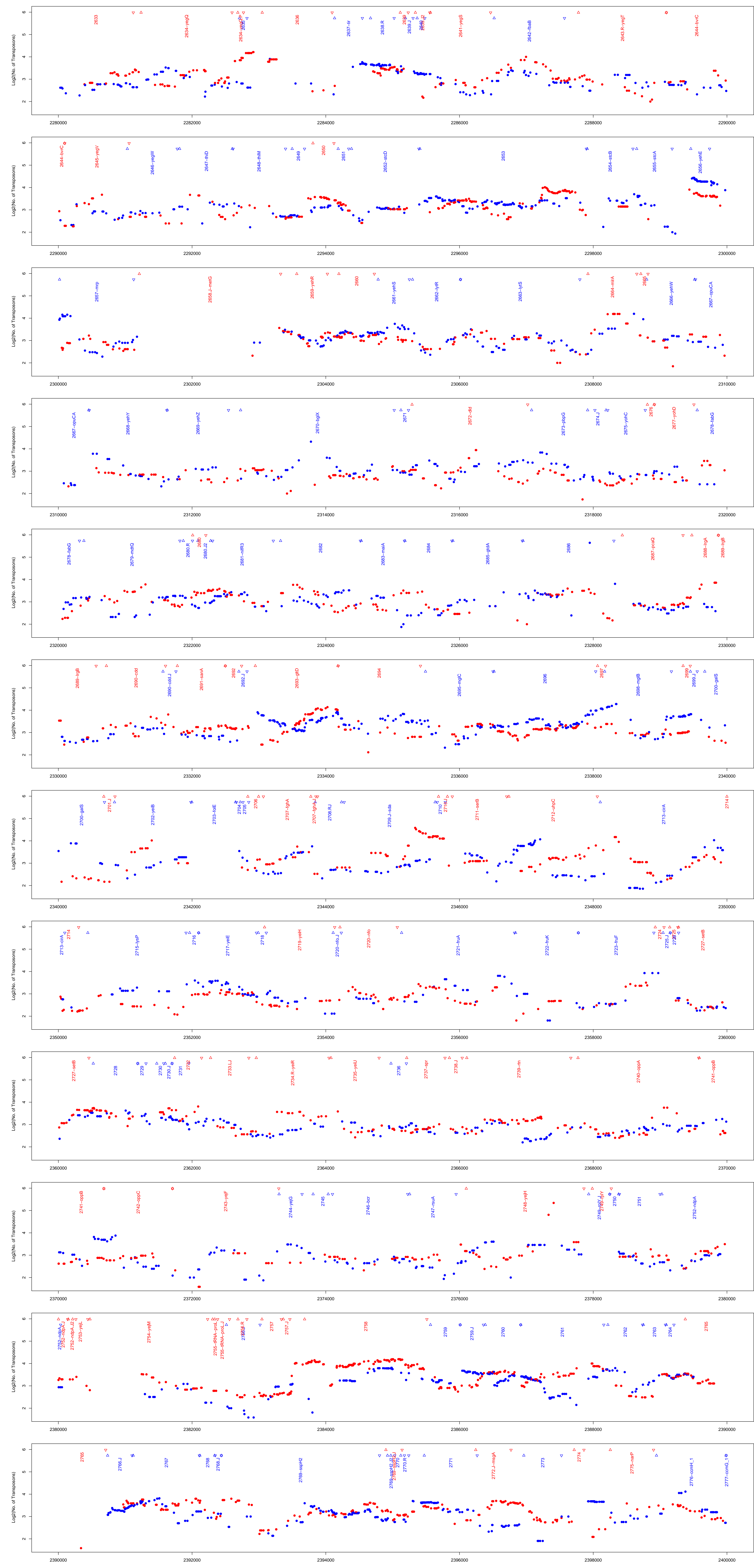

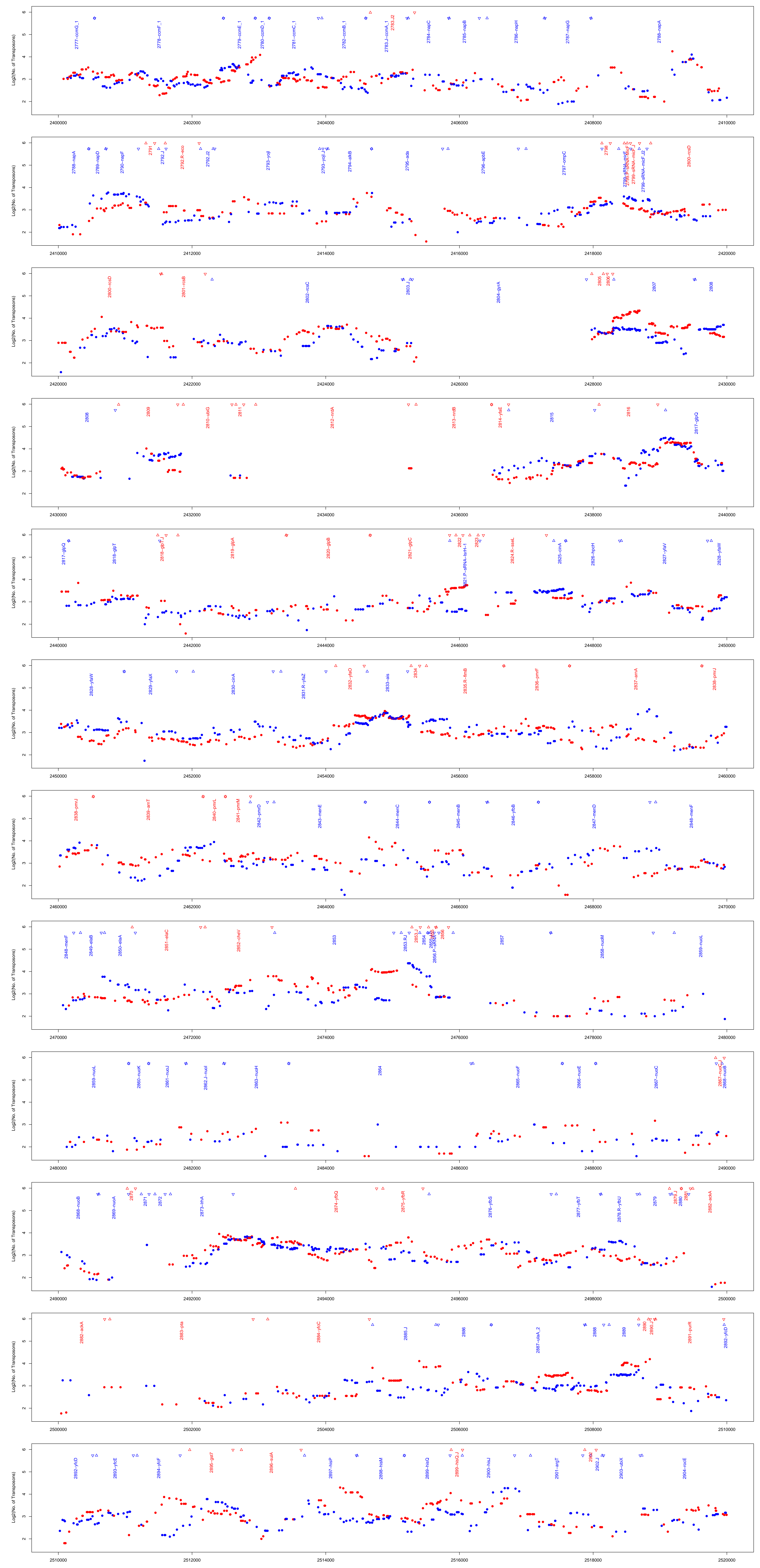

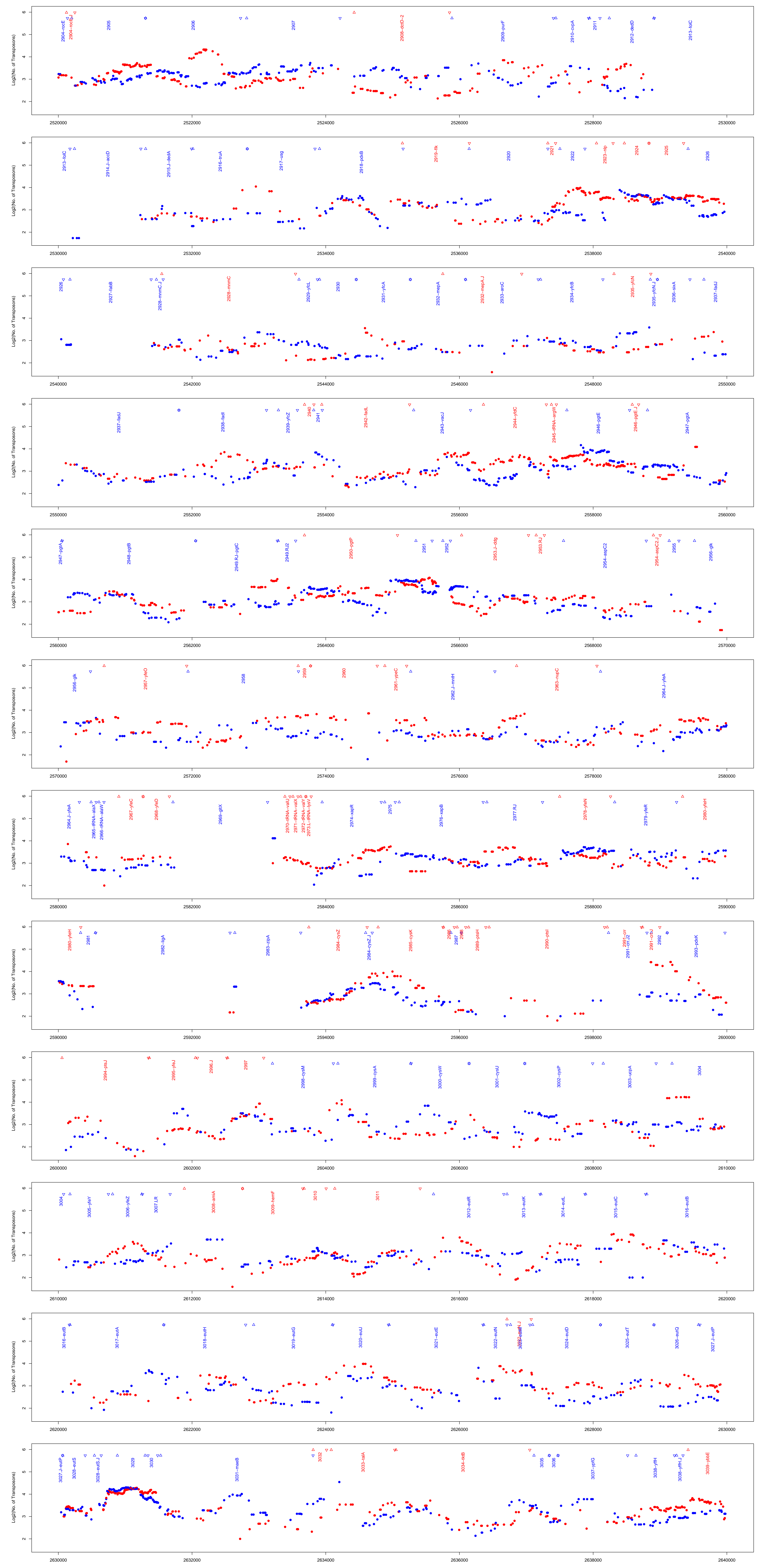

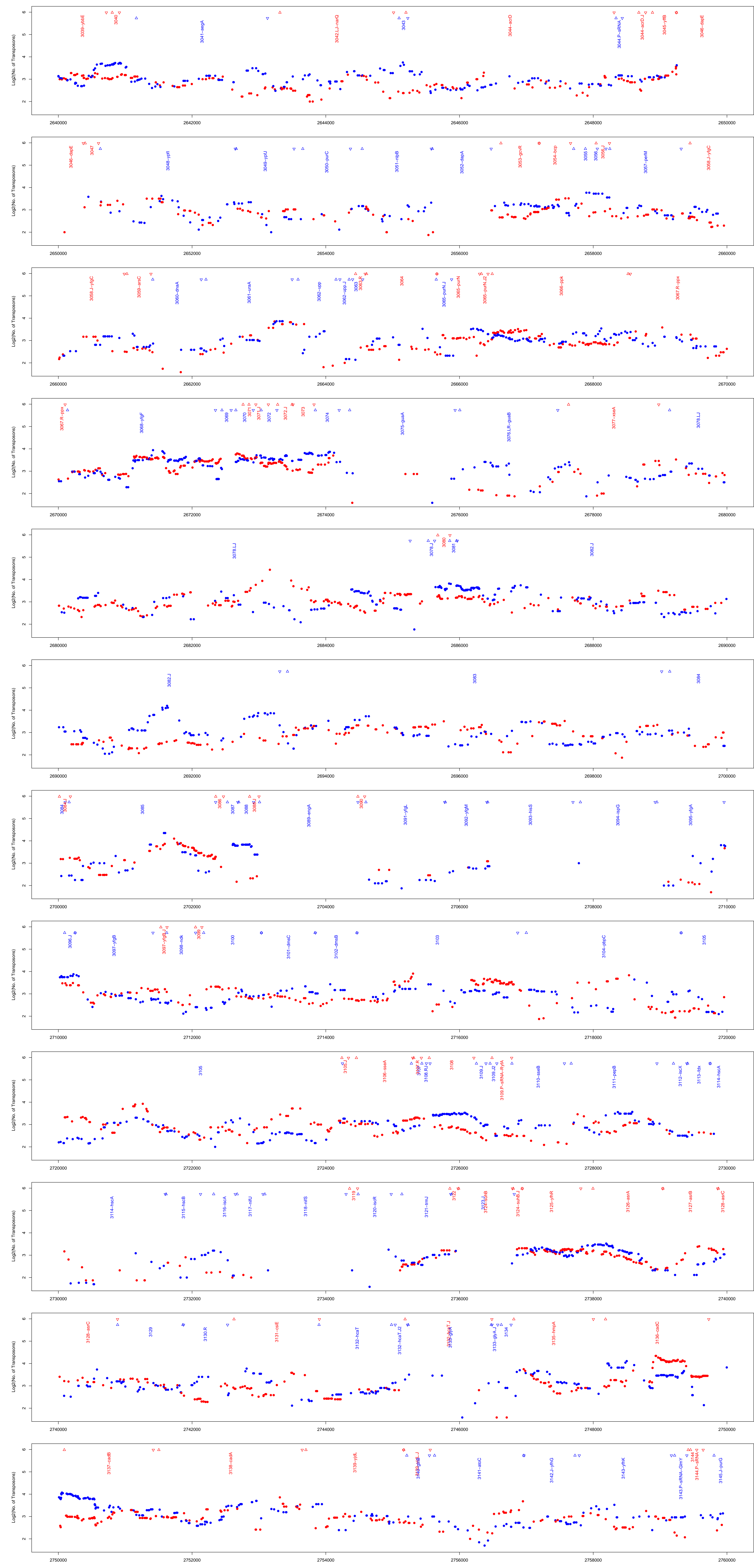

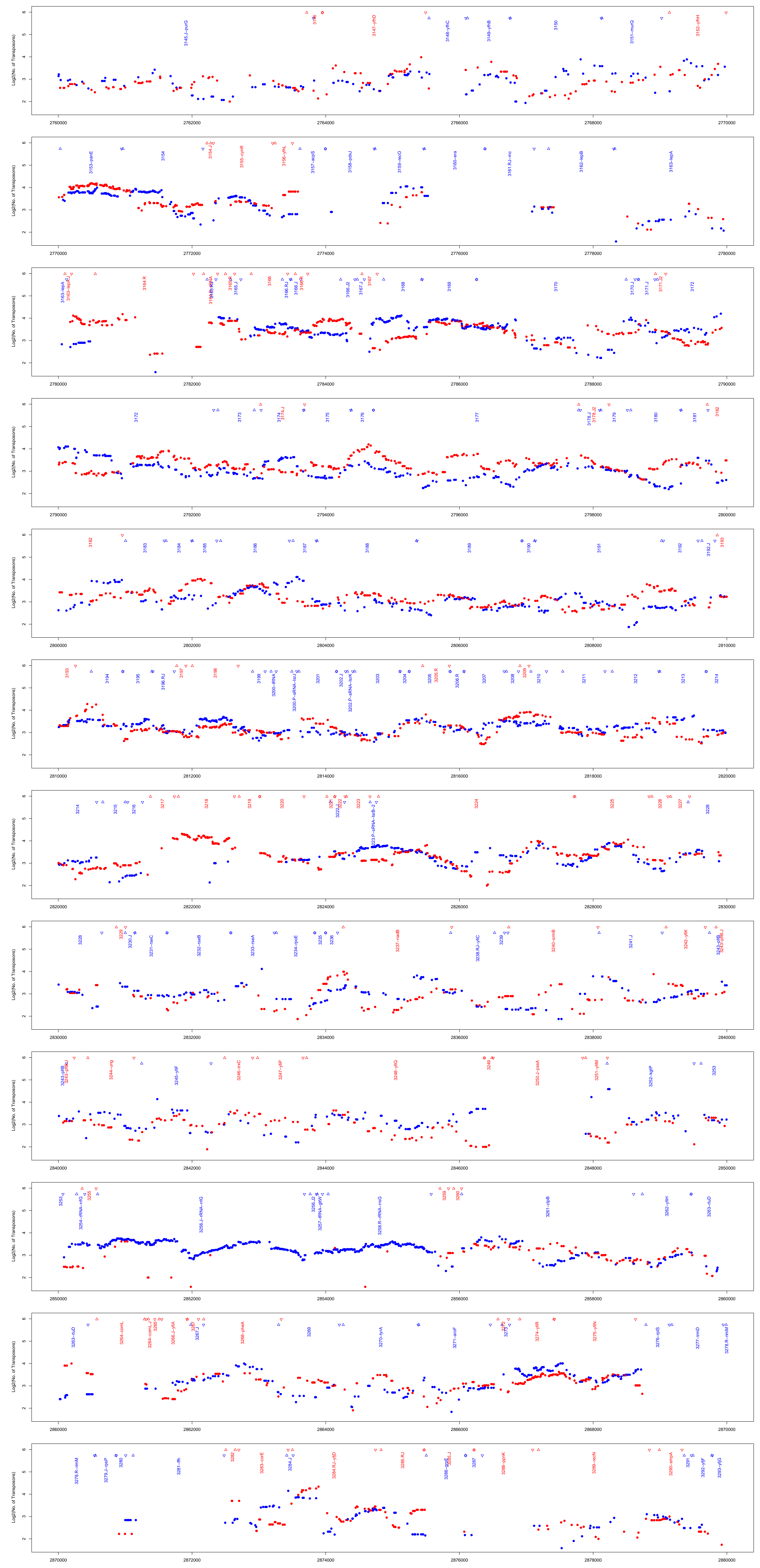

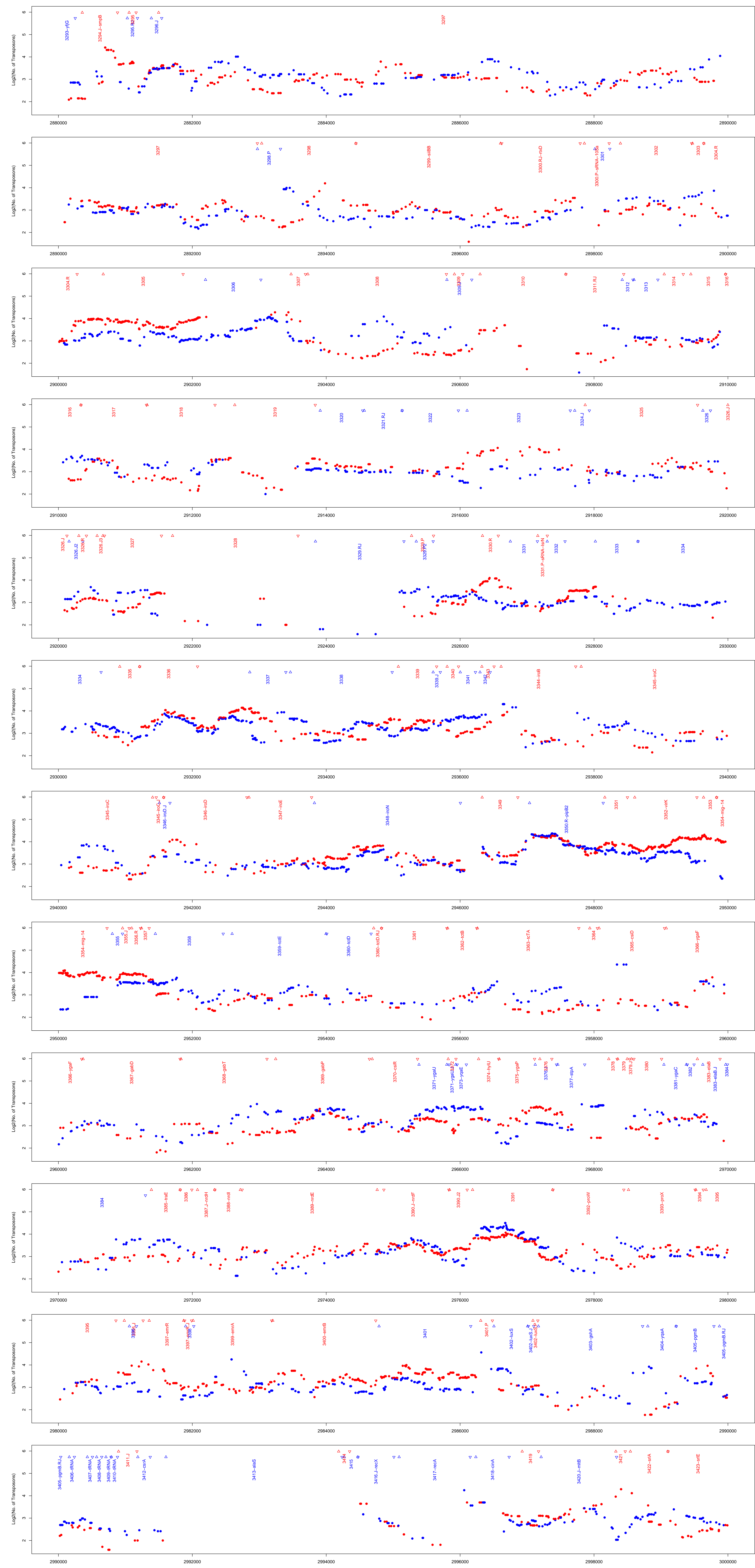

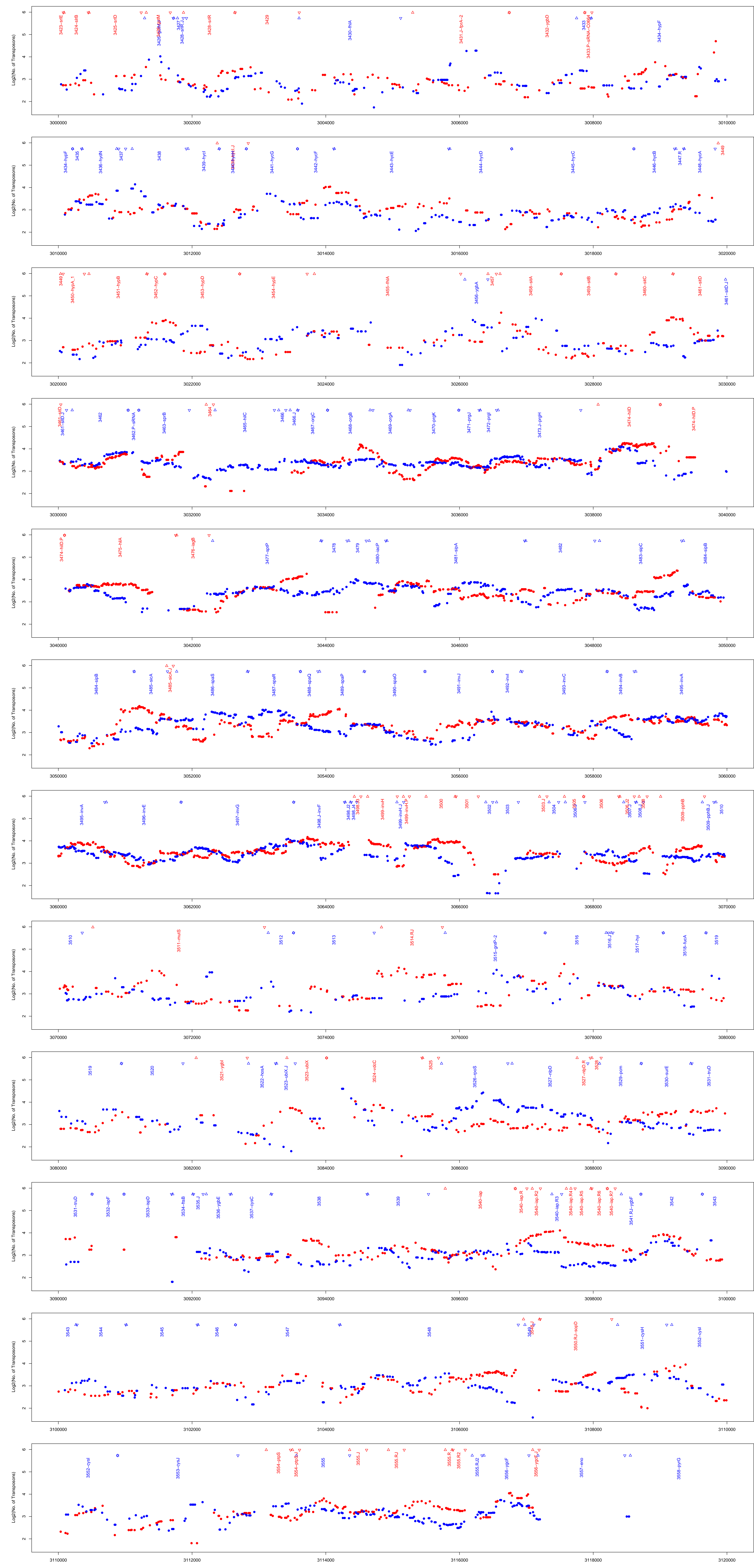

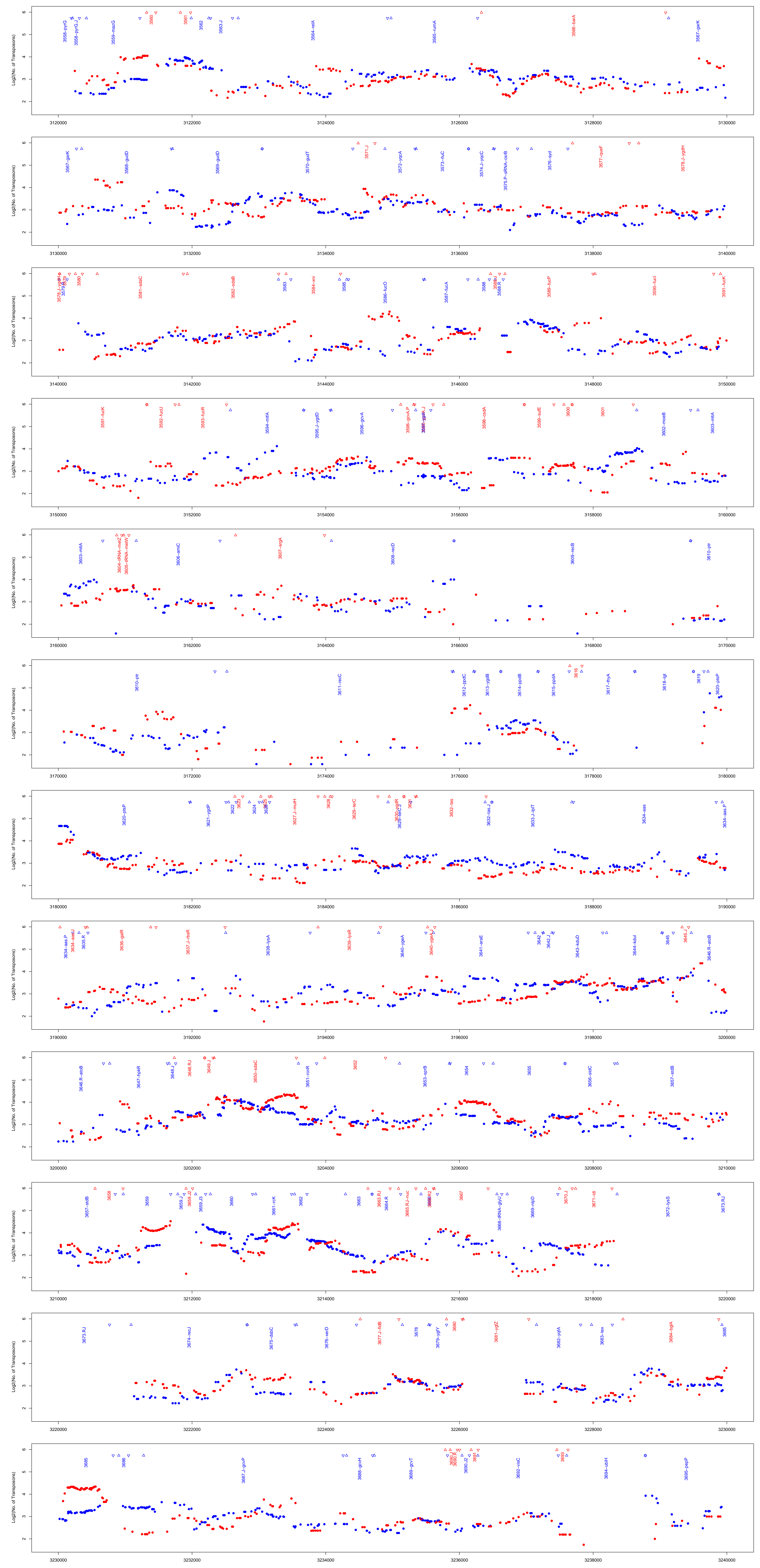

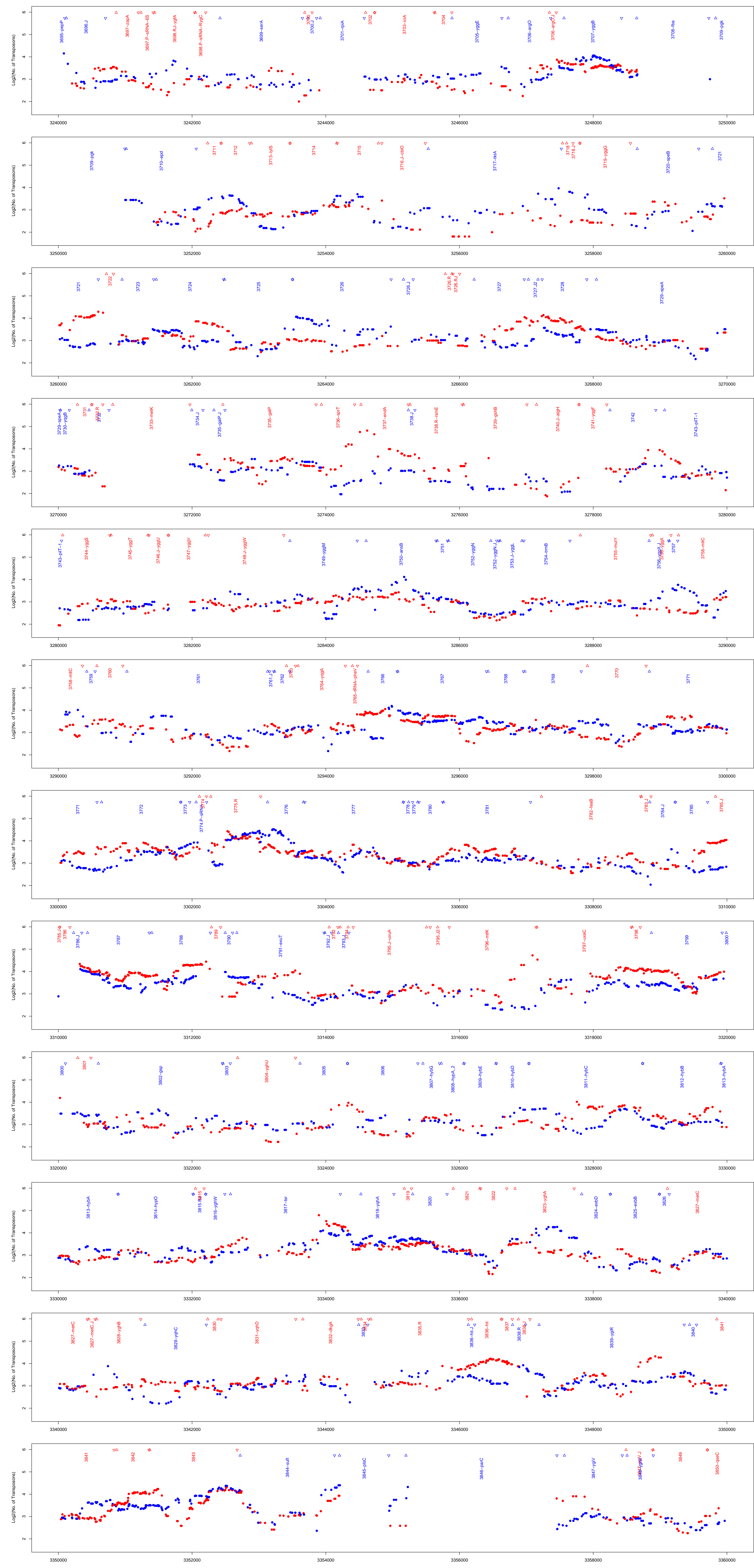

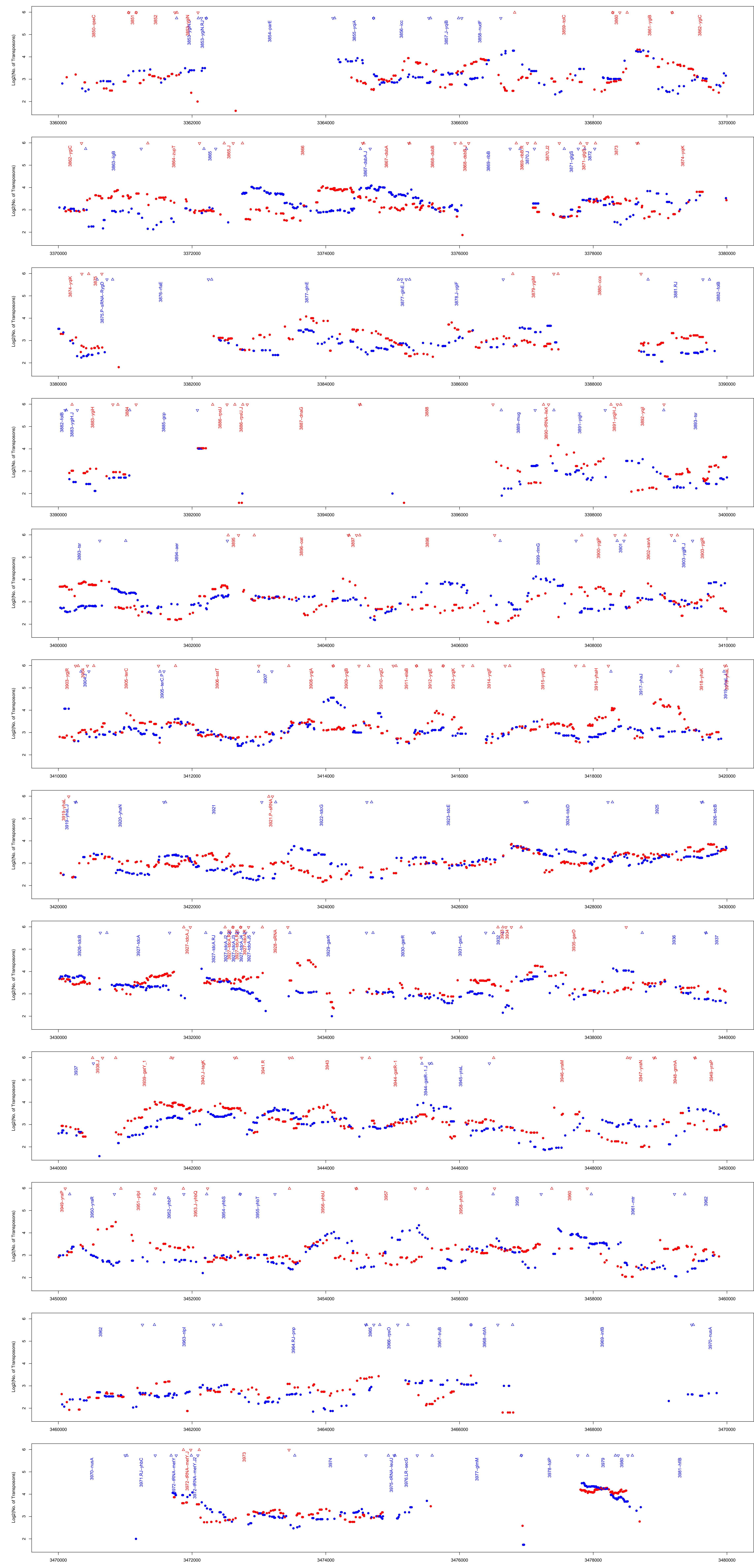

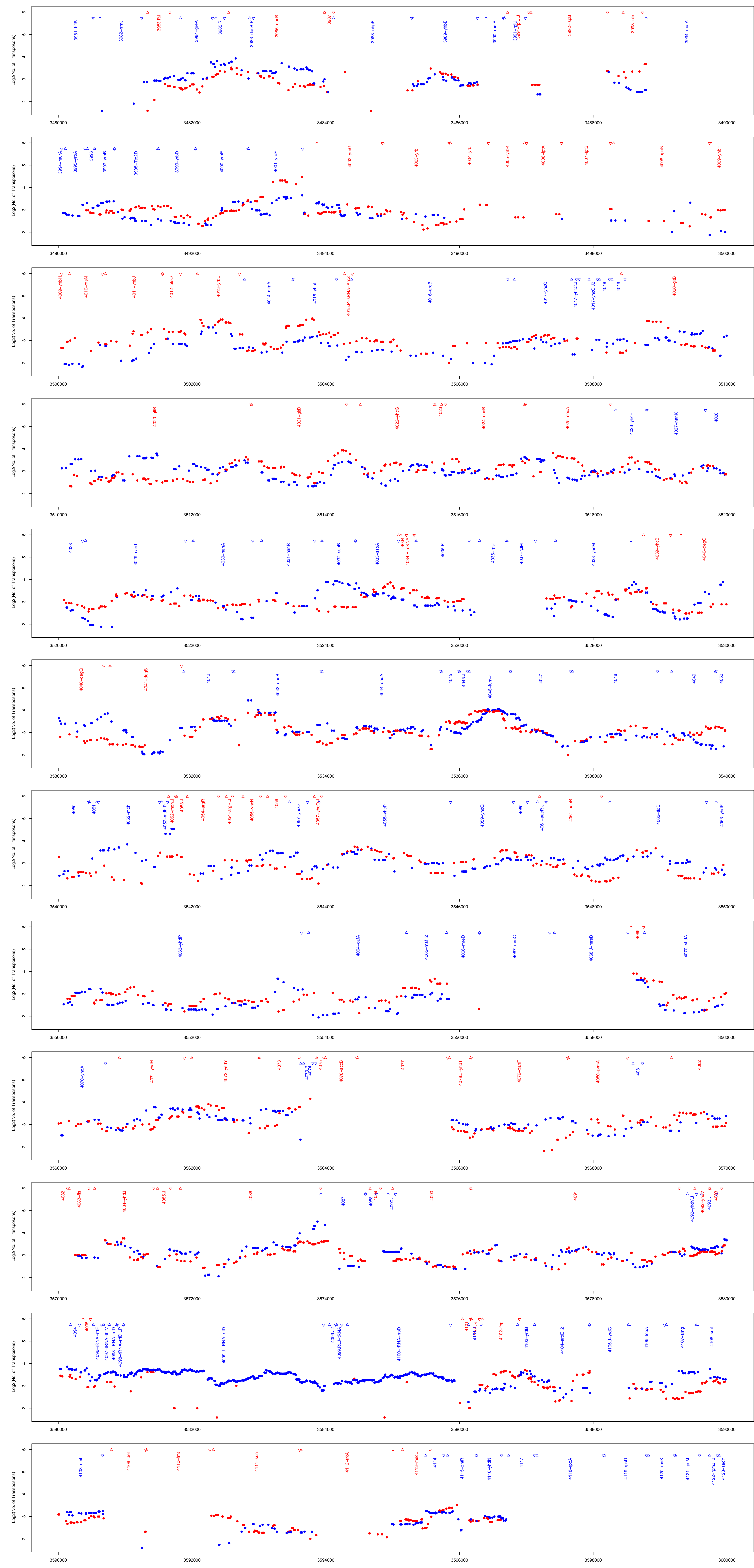

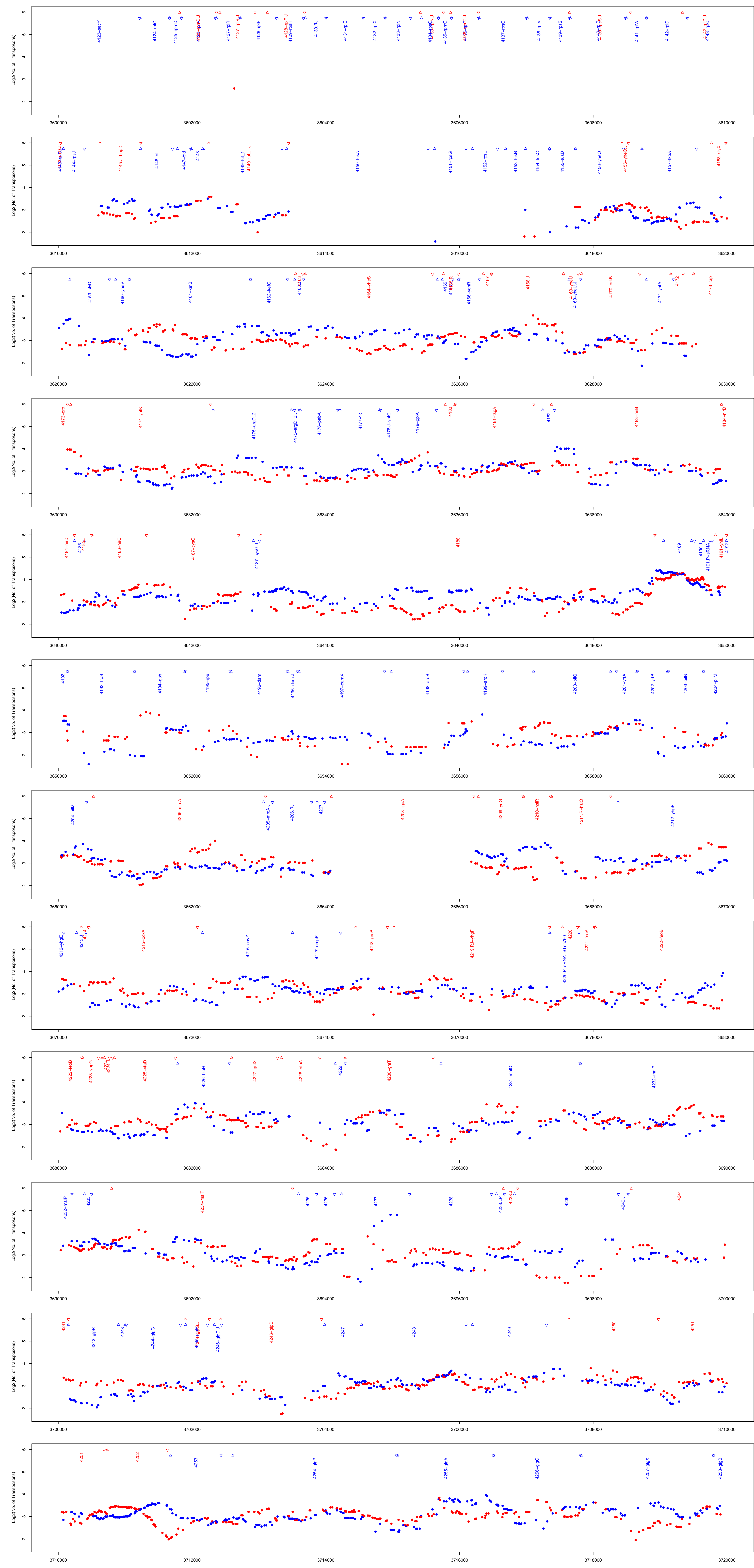

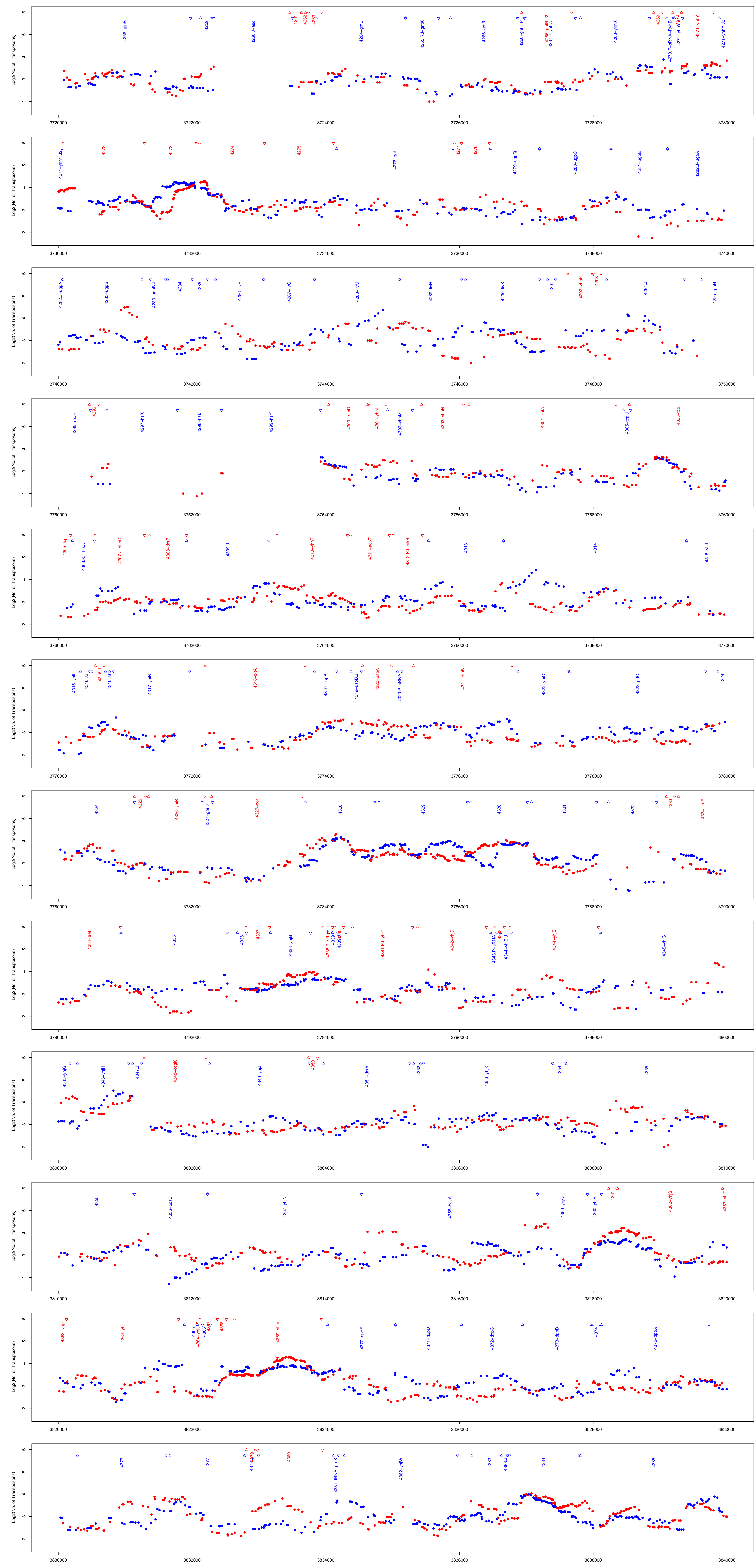

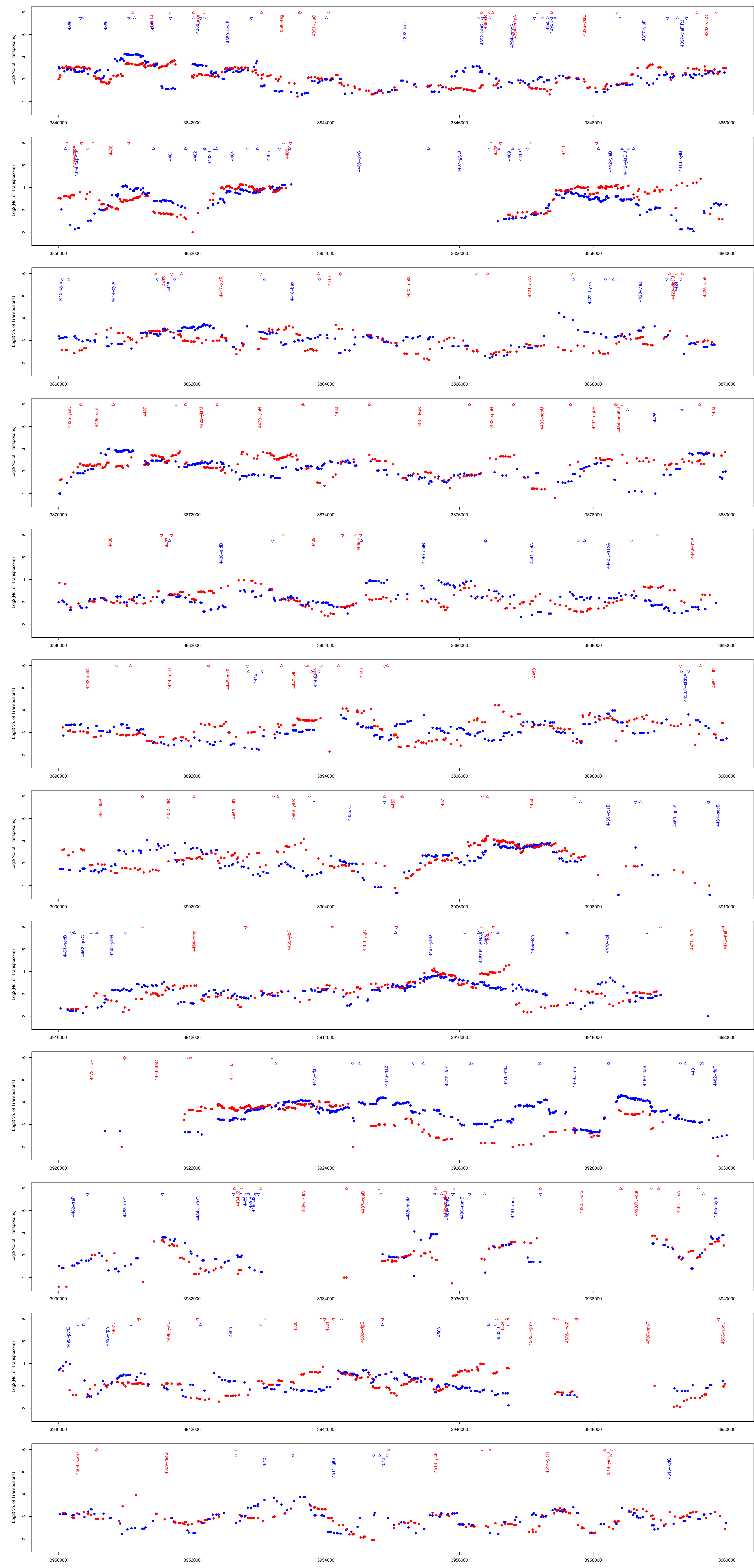

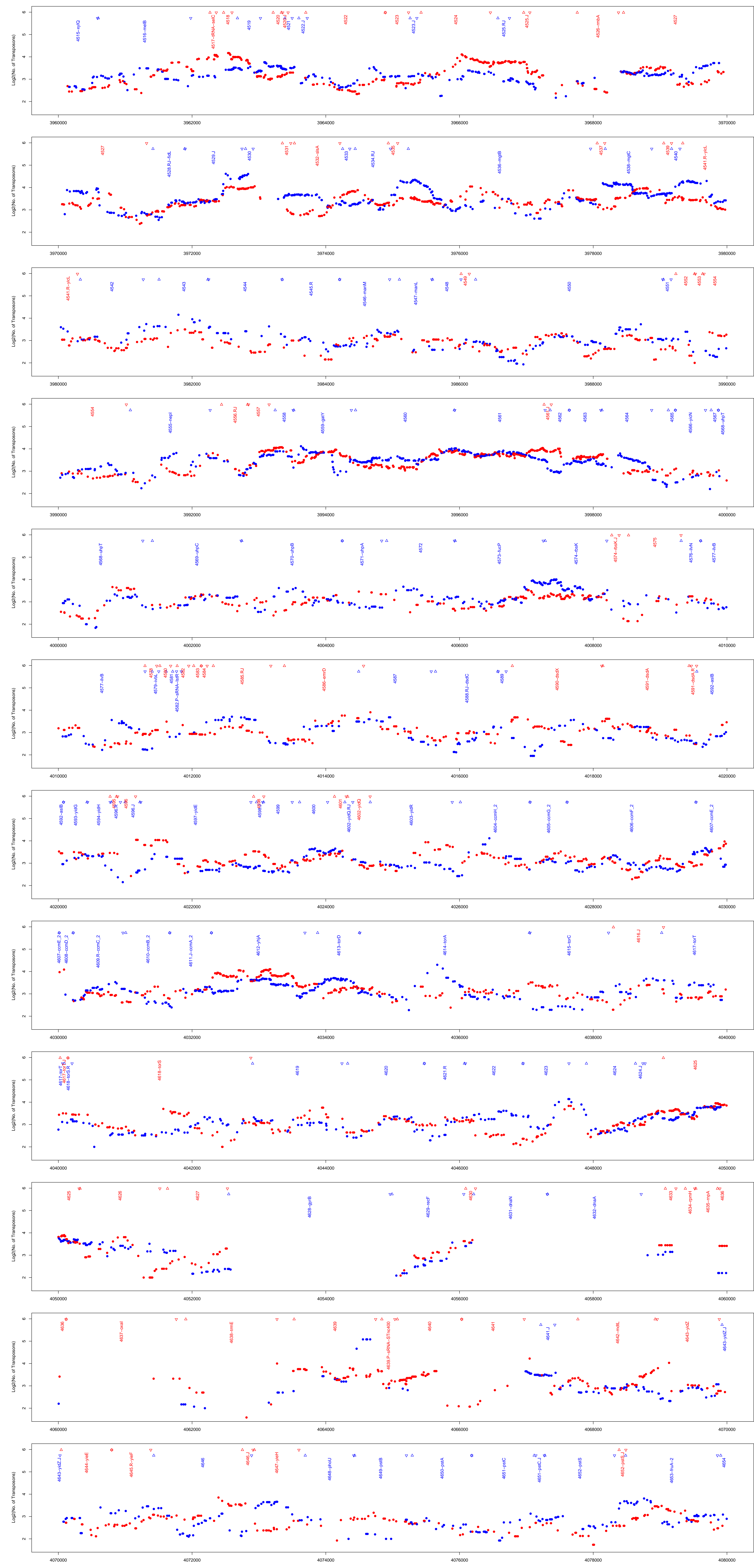

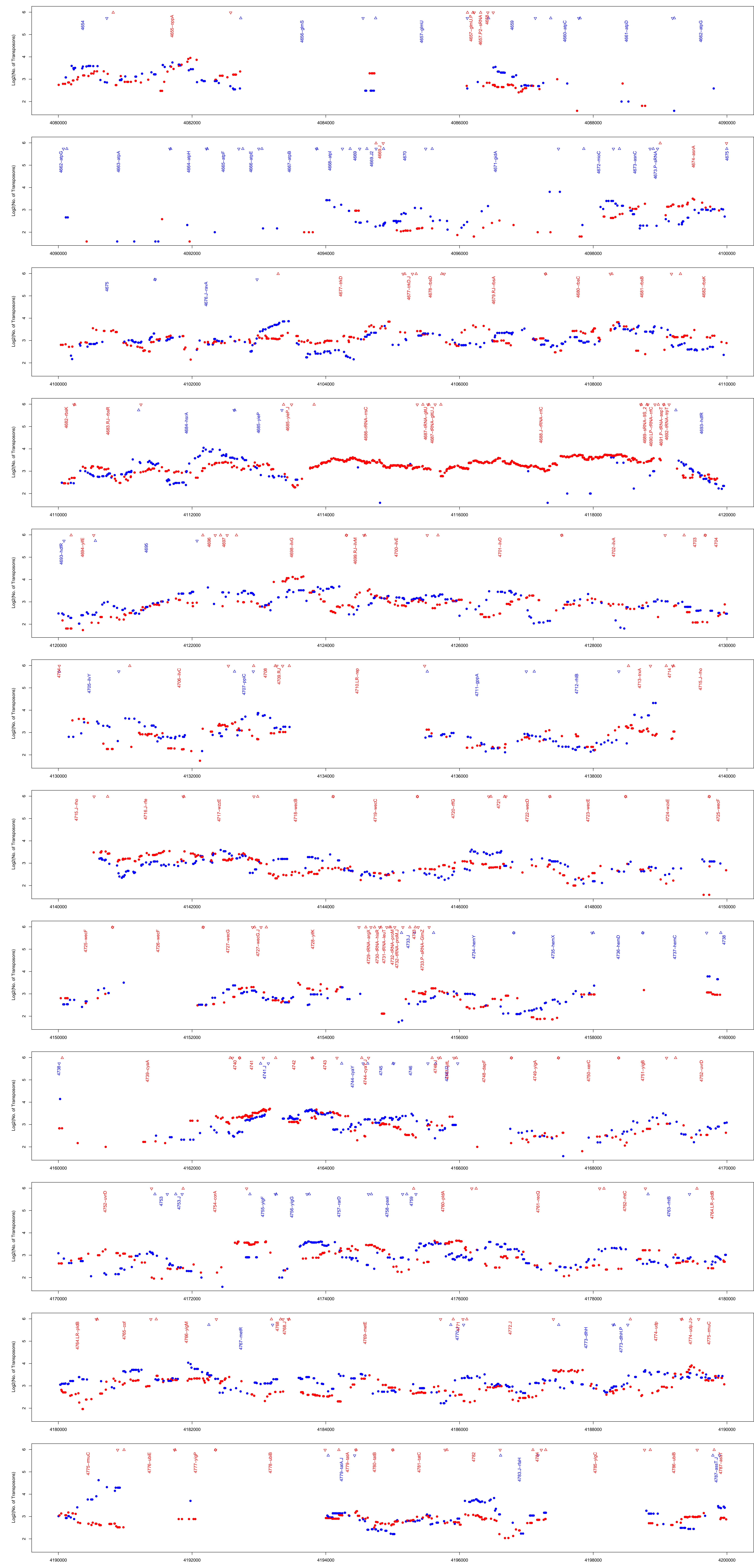

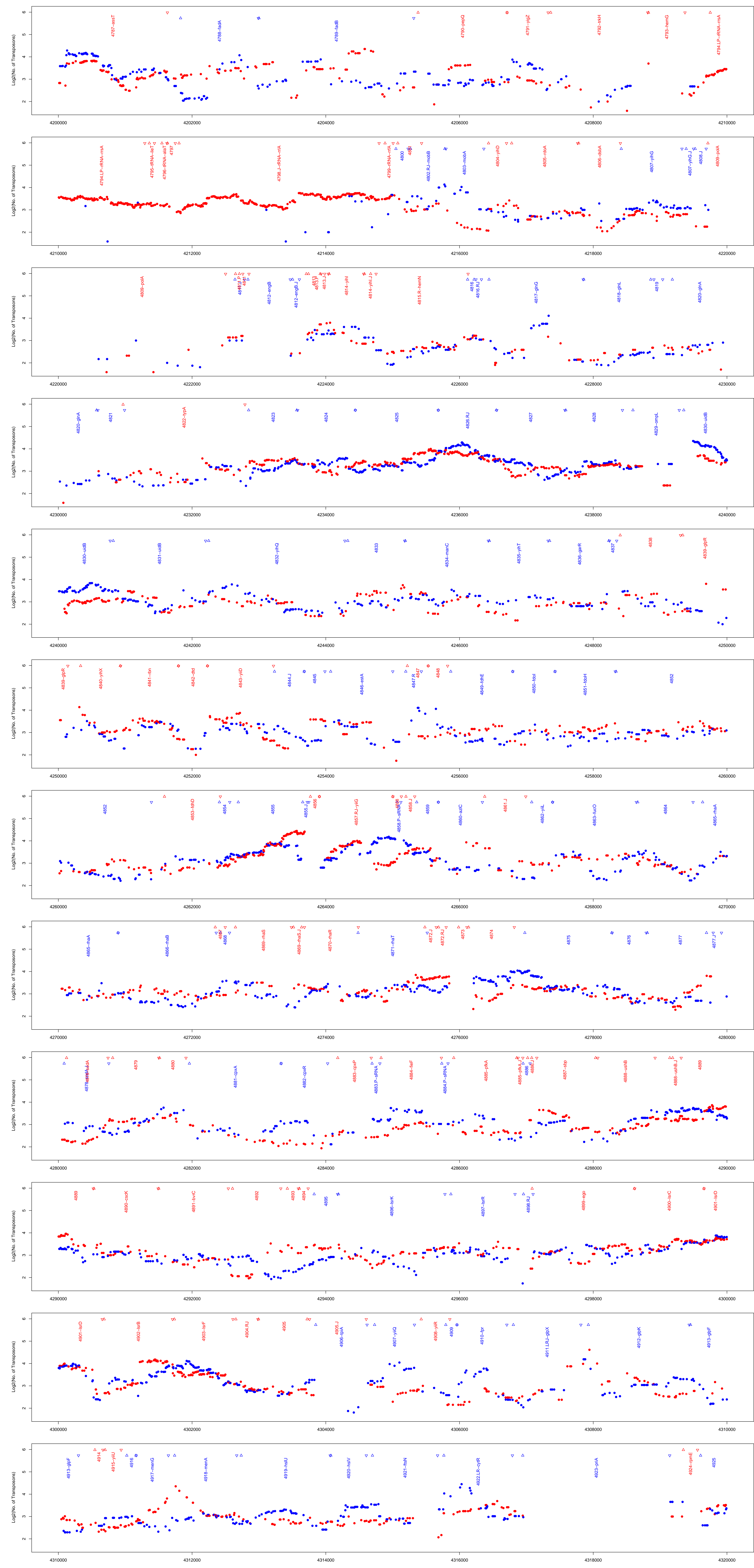

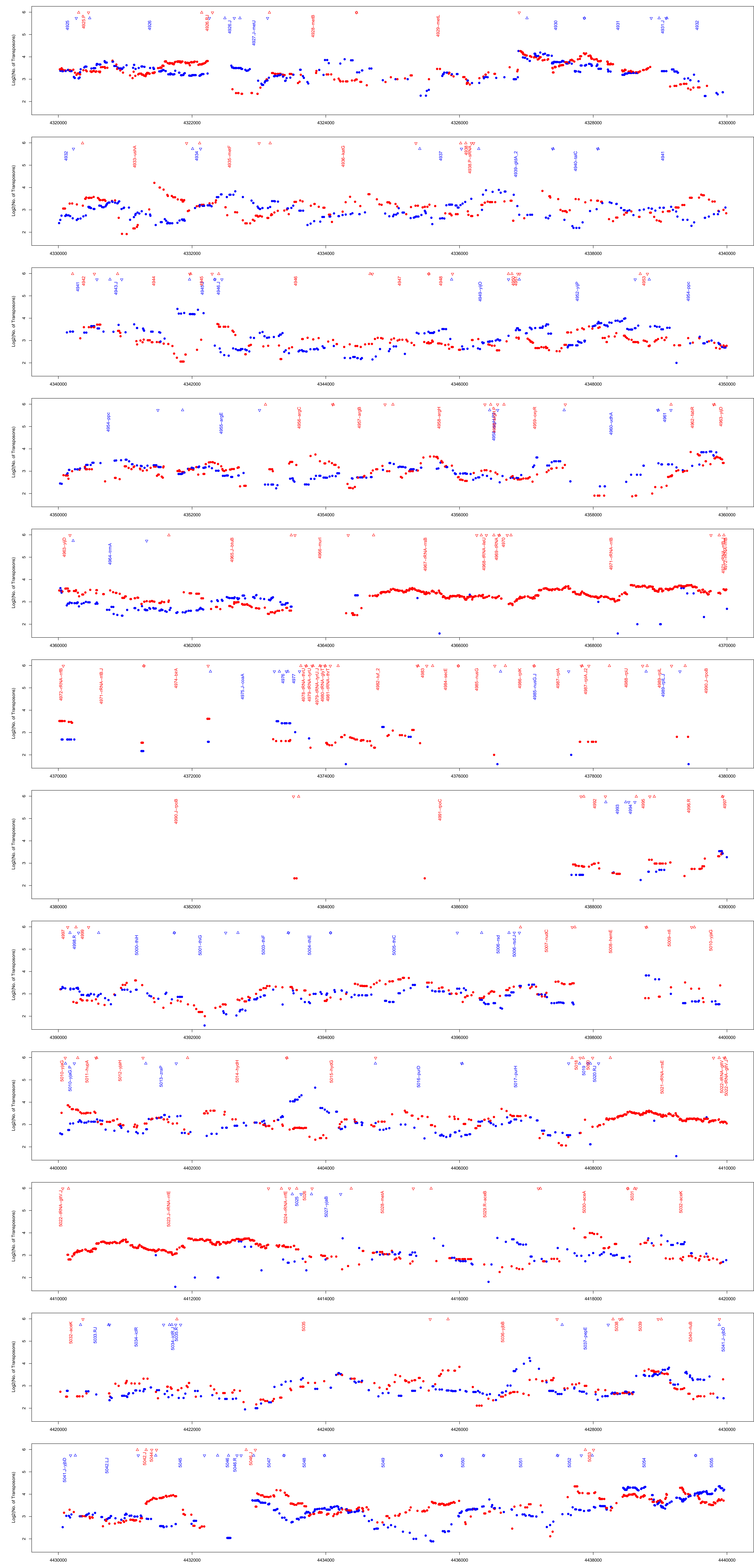

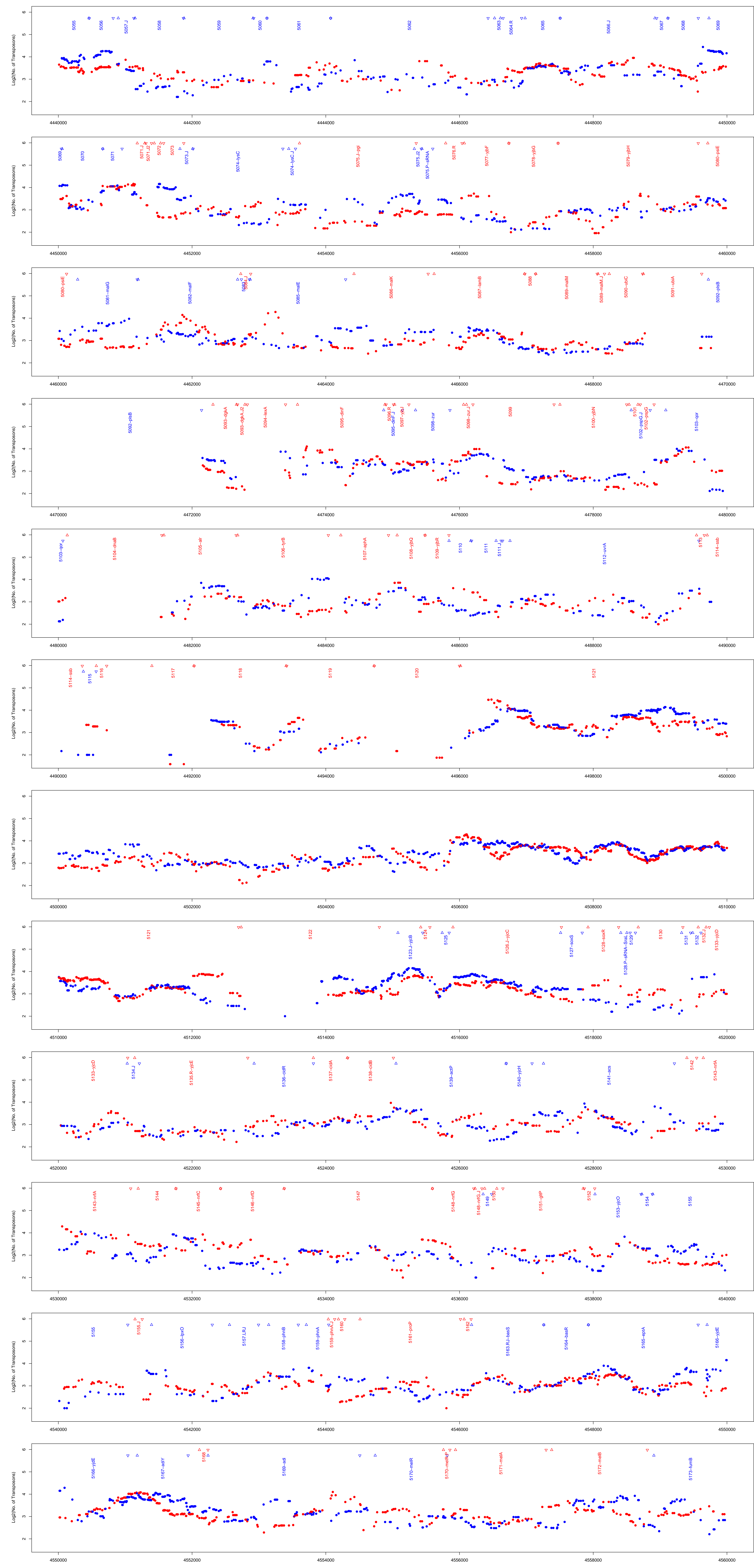

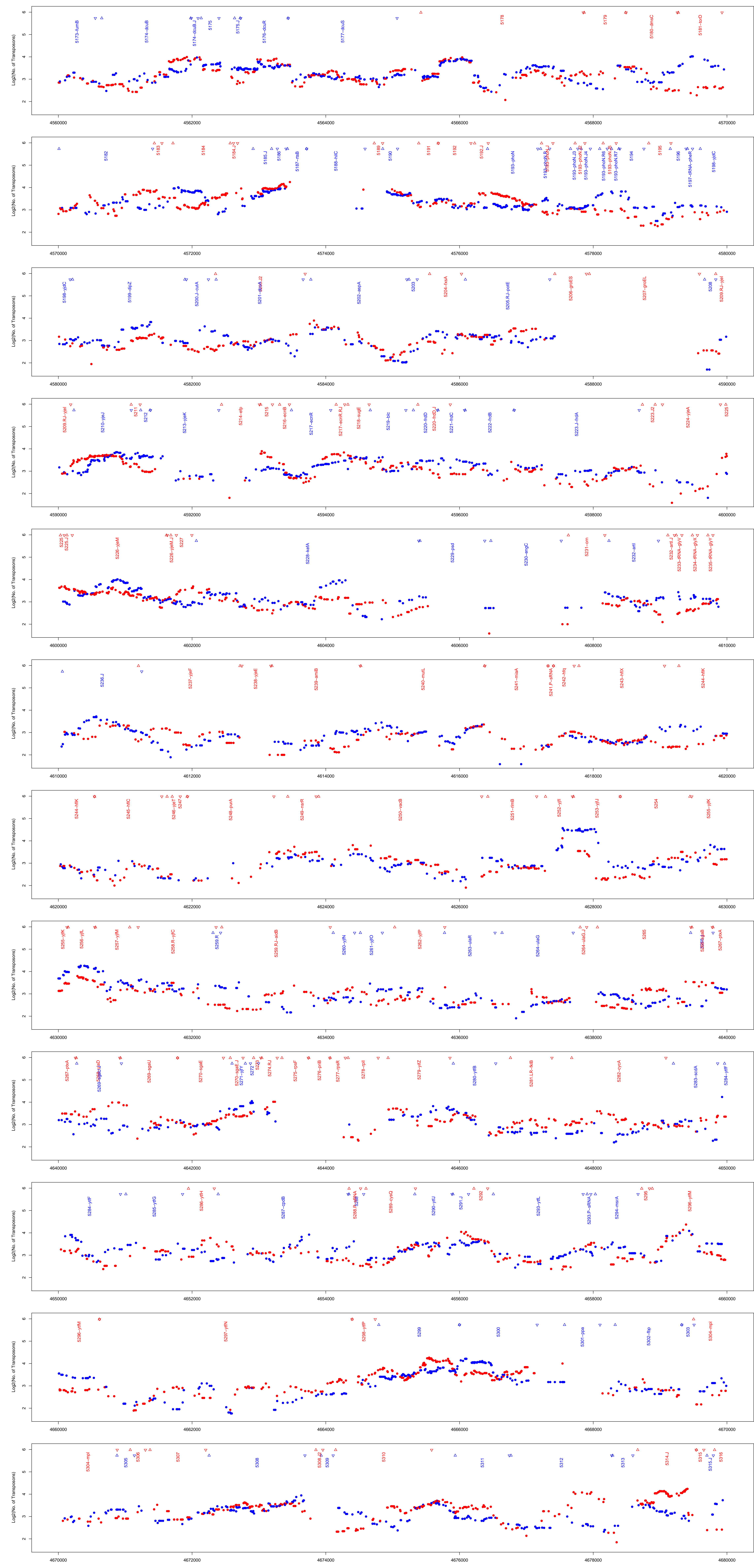

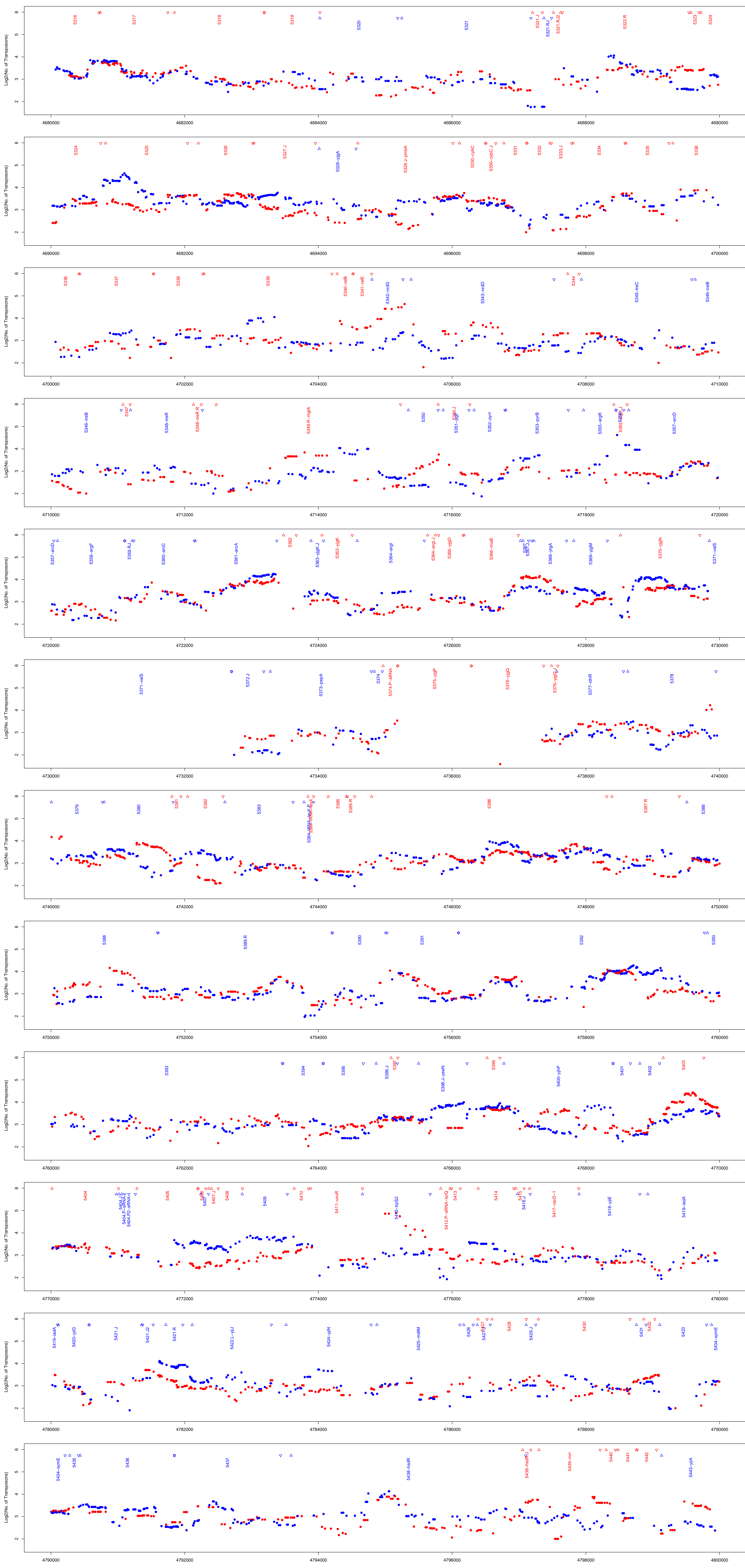

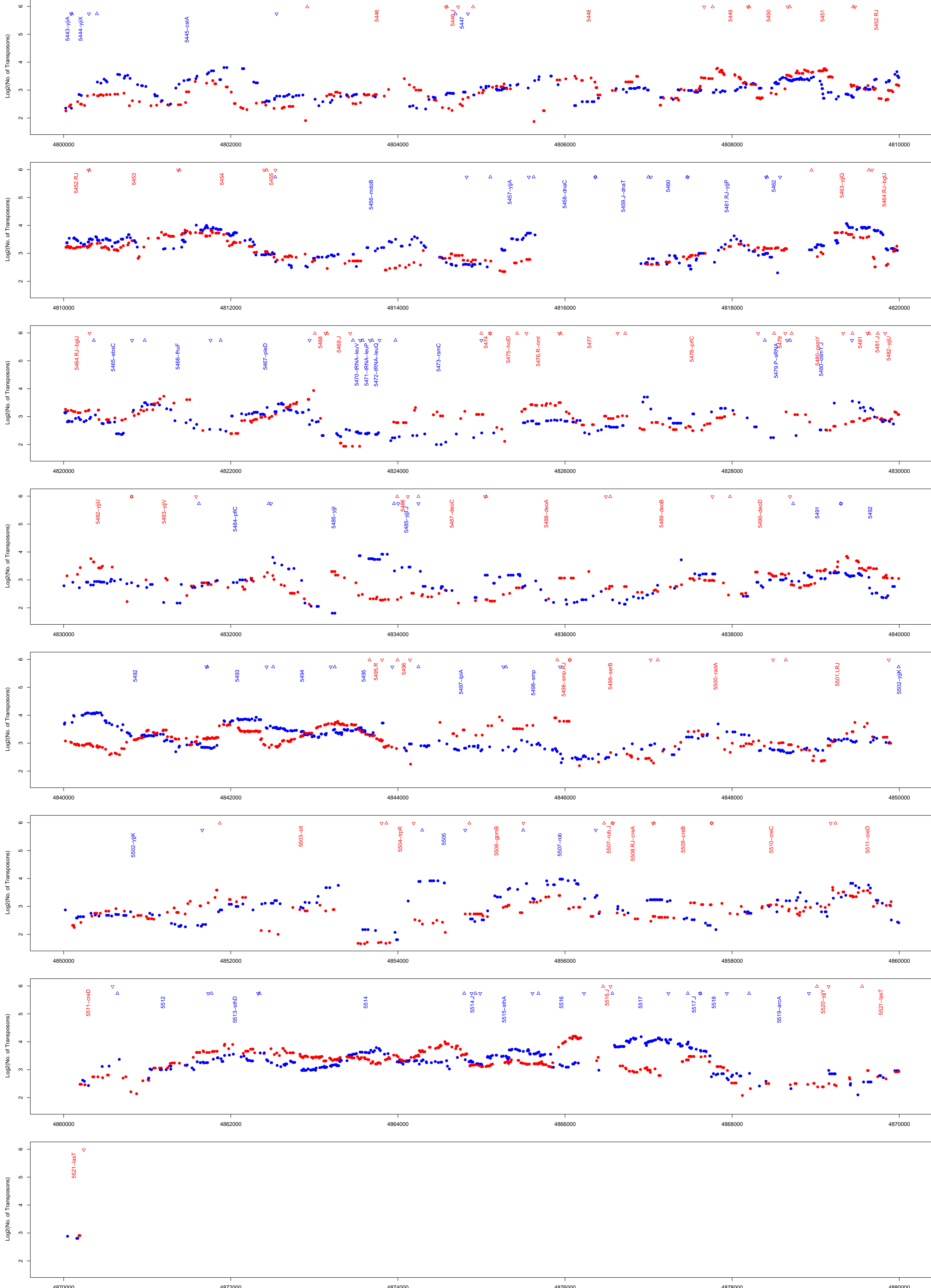

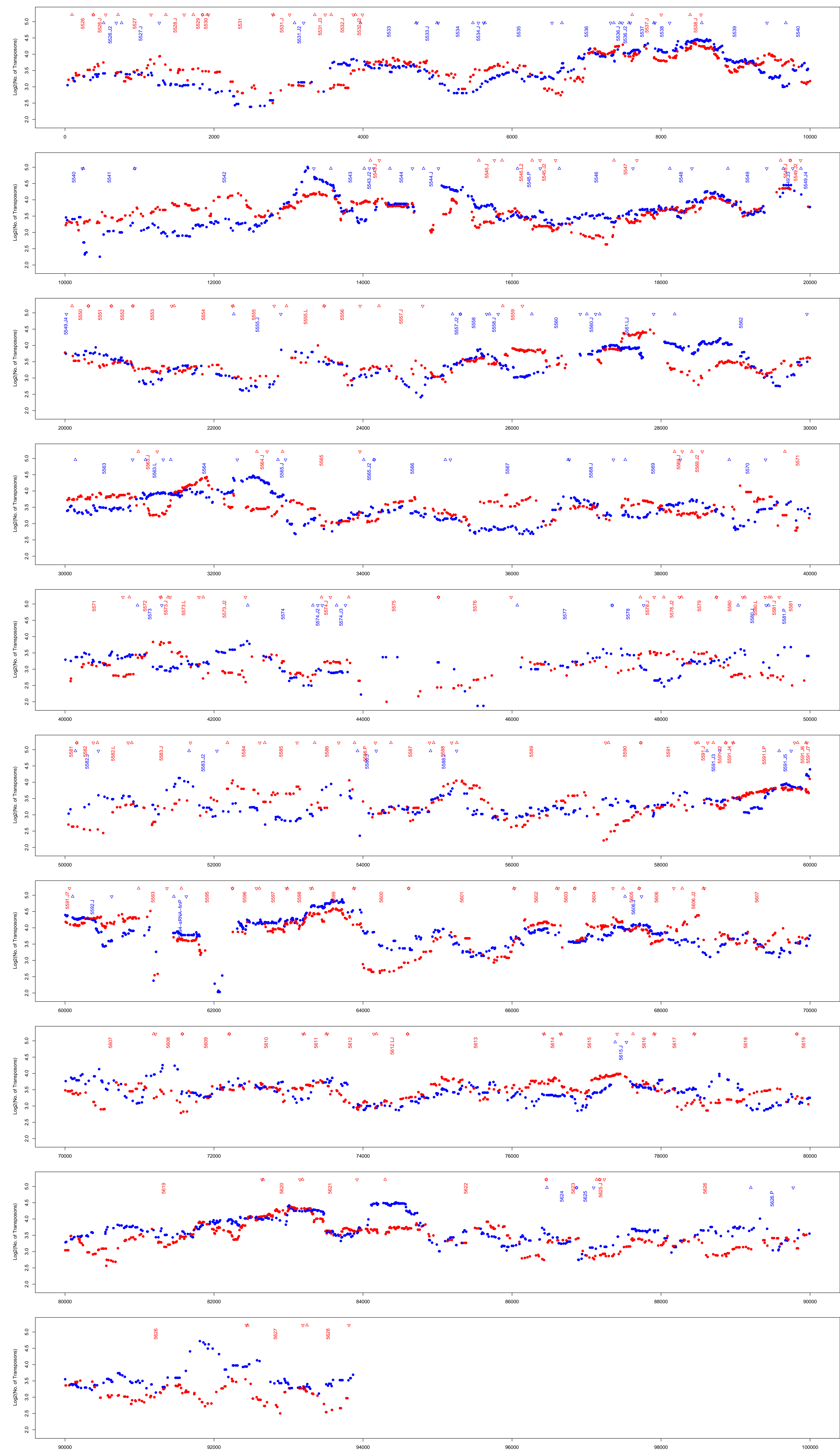

Supplement: Additional file 4 — Figure S2. Transposon insertion frequency across the S. Typhimurium 14028 s genome. [file 1471-2164-13-212-S4.pdf]
